# Isolation and Structural Elucidation of New Amphidinol Analogues from *Amphidinium carterae* Cultivated in a Pilot-Scale Photobioreactor

**DOI:** 10.3390/md19080432

**Published:** 2021-07-29

**Authors:** Adrián Morales-Amador, Alejandro Molina-Miras, Lorenzo López-Rosales, Asterio Sánchez-Mirón, Francisco García-Camacho, María L. Souto, José J. Fernández

**Affiliations:** 1 Instituto Universitario de Bio-Orgánica Antonio González (IUBO AG), Universidad de La Laguna (ULL), Avda. Astrofísico F. Sánchez 2, 38206 La Laguna, Spain; amoralea@ull.edu.es; 2 Departamento de Química Orgánica, Universidad de La Laguna (ULL), Avda. Astrofísico F. Sánchez 2, 38206 La Laguna, Spain; 3 Chemical Engineering Department, University of Almería, 04120 Almería, Spain; amm657@ual.es (A.M.-M.); llr288@ual.es (L.L.-R.); asmiron@ual.es (A.S.-M.); fgarcia@ual.es (F.G.-C.); 4 Research Center CIAIMBITAL, University of Almería, 04120 Almería, Spain

**Keywords:** amphidinol, *Amphidinium carterae*, dinoflagellate microalgae, photobioreactor, hemolysis

## Abstract

The demand for valuable products from dinoflagellate biotechnology has increased remarkably in recent years due to their many prospective applications. However, there remain many challenges that need to be addressed in order to make dinoflagellate bioactives a commercial reality. In this article, we describe the technical feasibility of producing and recovering amphidinol analogues (AMs) excreted into a culture broth of *Amphidinium carterae* ACRN03, successfully cultured in an LED-illuminated pilot-scale (80 L) bubble column photobioreactor operated in fed-batch mode with a pulse feeding strategy. We report on the isolation of new structurally related AMs, amphidinol 24 (**1**, AM24), amphidinol 25 (**2**, AM25) and amphidinol 26 (**3**, AM26), from a singular fraction resulting from the downstream processing. Their planar structures were elucidated by extensive NMR and HRMS analysis, whereas the relative configuration of the C-32→C-47 bis-tetrahydropyran core was confirmed to be antipodal in accord with the recently revised configuration of AM3. The hemolytic activities of the new metabolites and other related derivatives were evaluated, and structure–activity conclusions were established. Their isolation was based on a straightforward and high-performance bioprocess that could be suitable for the commercial development of AMs or other high-value compounds from shear sensitive dinoflagellates.

## 1. Introduction

Dinoflagellates are a well-recognized source of bioactives exhibiting wide diverse functionality and distinctive chemical structures that show great potential for use in the fields of biology, biomedicine, pharmacology and toxicology [1]. However, several difficulties remain in attempts to implement and commercialize these bioactive secondary metabolites [2]. The limited availability of natural sources, along with their exceedingly complex synthesis or the lack of knowledge of biotic and abiotic growth conditions, extreme shear sensitivity in photobioreactor culture, have greatly hampered their commercial development [3,4,5].

Despite the above difficulties, we have recently developed strategies related to the production of pilot-scale cultures of dinoflagellates of the genus *Amphidinium* [6,7,8,9]. *Amphidinium* species are known to produce super carbon chain compounds including amphidinols (AMs) and other related metabolites [10,11,12,13,14]. This growing family of opened long-chain polyketides, characterized by a hairpin shape constituted by a central common core delimited by two tetrahydropyran rings separating two moieties—one mainly polyhydroxilic and other polyenic—is known to elicit potent antifungal, ichthyotoxic, hemolytic, cytotoxic, antiprotozoan or antidiatom activities [15]. In addition, AMs also exhibit strong toxicity against some problematic human pathogens, such as *Candida albicans* fungus and bacteria belonging to *Mycoplasma* genus [16,17]. Antifungal and hemolytic activities are believed to correlate their structural features with their interaction with phospholipid bilayers that lead to membrane permeabilization, which is believed to be independent of membrane thickness but dependent on membrane sterols [18].

The basis of a bioprocess strategy for achieving technically feasible recovery of AMs excreted into the broth by photoautotrophic culture of *Amphidinium carterae* using a simple and scalable process was assessed [6]. AMs have never been detected or recovered from supernatants of *Amphidinium* cultures; the results reported in the literature refer to AMs extracted from biomass pellets. Briefly, the bioprocess consists of three distinct parts: (1) culture of *A. carterae* strain ACRN03 in a pilot-scale (80 L) bubble column photobioreactor illuminated with multi-color LEDs operated in fed-batch mode with a pulse feeding strategy to produce and recovery microalgal biomass and supernatant; (2) separation of an AMs-enriched extract from the supernatant by reverse phase chromatography; and (3) chromatographic purification, identification, dereplication and structural resolution of metabolites. 

Excellent yields were obtained for a new analog named amphidinol 20B [19], as well as the AMs luteophanol D and lingshuiol A, previously reported in other strains [20,21]. The concentrations of luteophanol D and lingshuiol A in the supernatant of *A. carterae* ACRN03 were much higher than those reported in cultures of other *Amphidinium* strains where both metabolites were recovered from cells and not from the processed cell-free culture medium, despite the recovery of AMs excreted to culture medium presenting advantages with respect to their intracellular counterparts from a downstream processing point of view [6]. Known and novel AMs are also expected to be present in variable quantities in the cell pellets and in the remaining supernatant resulting from centrifugation step in the clarification system. Herein, our advances tracing the presence of this type of derivatives in the singular fraction resulting from the remainder supernatant in the clarification system were point out. As a result, this work report on the isolation, structure determination and activity evaluation of three new derivatives, AMs 24–26 (**1**–**3**).

## 2. Results and Discussion

### 2.1. Isolation and Structural Elucidation of AMs

**Figure 1:**
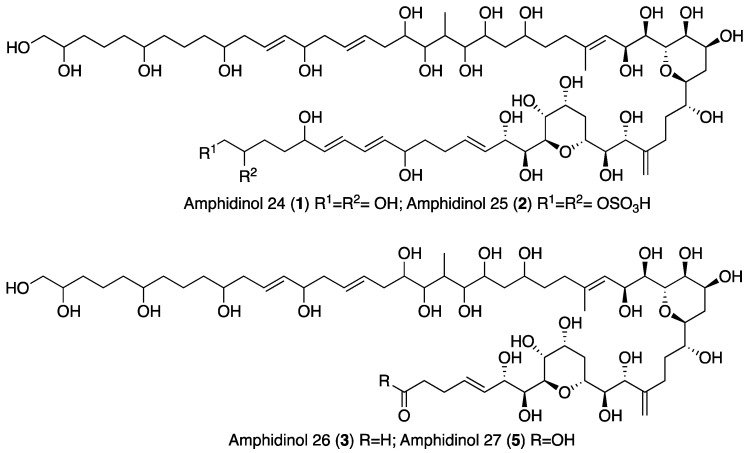
Structures of new AMs identified in *Amphidinium carterae* cultures.

When the objective of a marine dinoflagellate-based bioprocess is the identification and production of relatively minority specific secondary metabolites, such as AMs from *A. carterae*, the recovery of the largest amount possible of AMs becomes a priority. In the process of biomass harvesting from the *Amphidinium* photobioreactor culture, three fractions were clearly identified where the presence of AMs was likely. The first corresponded to the wet cell biomass pellet, a second one was related to the clarified cell-free culture medium, and a third included the supernatant (1 L) that remained in the clarification equipment. The presence of AMs in the second one was analyzed in a previous study [6]. Thus, in this work, the third fraction was investigated. The methanolic extract (10.9 g) from the reddish lyophilized supernatant was subjected to a series of chromatographic steps to provide two known AM derivatives (luteophanol D and AM20B) and three new AM derivatives, AM24 (**1**) 6.8 mg; AM25 (**2**) 1.2 mg, and AM26 (**3**) 1.8 mg (Figure 1).

**Figure 2:**
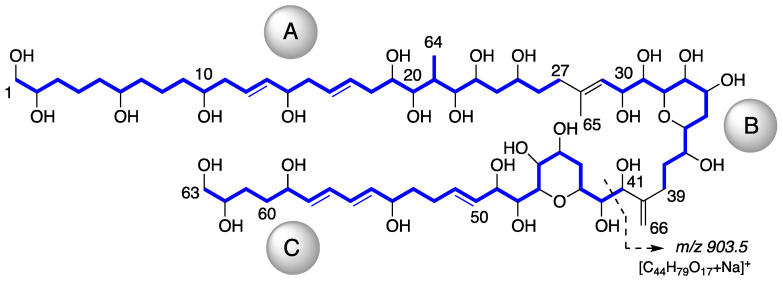
Partial structures obtained from COSY, TOCSY, HSQC, HSQC-TOCSY and H2BC analysis of AM24 (**1**) (Blue lines). Key fragmentation pattern for AMs 24–26 (**1**–**3**) observed in MS/MS spectra.

The molecular formula C_66_H_116_O_27_ for AM24 (**1**) was determined by HRESIMS analysis (*m/z* 1363.7606 [M + Na]^+^; calcd. 1363.7602), accounting for a highly oxygenated molecule with nine degrees of unsaturation. The NMR data (Table 1) in CD_3_OD revealed a total of 66 carbons assigned to eleven sp^2^ methines, one sp^2^ methylene, twenty-seven oxygenated sp^3^ methines, two oxygenated sp^3^ methylenes, one sp^3^ methine, twenty sp^3^ methylenes, two methyls and two quaternary sp^2^ carbons. The ^1^H NMR spectrum showed considerable signal overlap, especially in the regions δ_H_ 1.30→2.40 and 3.40→4.15; hence, a meticulous analysis of COSY, TOCSY, HSQC, HSQC-TOCSY, and H2BC spectra, allowed the identification of three independent ^1^H—^1^H spin systems A–C (Figure 2). Thus, the structural resolution of Fragment A was started at the methylene carbon C-1 (δ_H_ 3.43, 3.48; δ_C_ 67.0) and the linear connections determined allowed us to establish the sequence up to the methylene carbon C-27 (δ_H_ 2.12, 2.21; δ_C_ 36.8), including the pendant methyl group C-64 (δ_H_ 0.98; δ_C_ 6.6) branched to C-21 (δ_H_ 2.30; δ_C_ 35.0). Analogously, the linear connections of sp^2^ methine carbon C-29 (δ_H_ 5.48; δ_C_ 125.9) to the methylene carbon C-39 (δ_H_ 2.10, 2.42; δ_C_ 27.8) unequivocally constructed the Fragment B. In addition, the rest of the observed linear connectivities revealed the Fragment C between the oxymethine C-41 (δ_H_ 4.18; δ_C_ 76.3) and the oxymethylene C-63 (δ_H_ 3.43, 3.48; δ_C_ 67.8) carbons. The partial structures A, B and C were linked together through quaternary carbons on the basis of key HMBC and H2BC correlations. Thus, the connection between the substructures A and B was secured by the correlations of protons H_2_-27, H-29, and those of the methyl group at C-65 (δ_H_ 1.75; δ_C_ 17.1) with C-28 (δ_C_ 139.0). Furthermore, cross-peaks of H_2_-39, H-41 and the sp^2^ methylene H_2_-66 (δ_H_ 4.99, 5.08; δ_C_ 112.8) with the sp^2^ quaternary carbon C-40 (δ_C_ 151.4) allowed us to join the two Fragments B and C (Figure 2). The planar structure was completed with the confirmation of the presence of two tetrahydropyran rings on the basis of two long-range correlations between H-36/C-32 and H-47/C-43. Further confirmation of the structure of **1** was obtained from mass spectral fragmentation (see Supporting Information, Figure S17).

**Figure 3:**
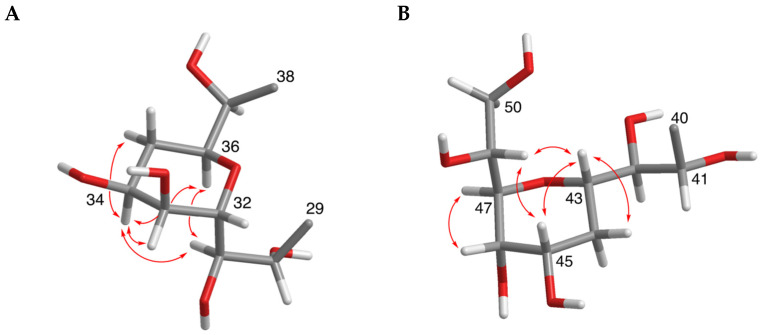
Relative configurations of the tetrahydropyran rings (C-32/C-36 (**A**) and C-43/C-47 (**B**)) and distinctive ROE interactions of compound **1**.

The relative configurations of the tetrahydropyran rings were deduced by distinctive NOE effects (Figure 3). NOE interactions between H-31/H-36, H-31/H-34 and H-34/H-36 suggested a chair conformation for the ring from C-32 to C-36 with H-34 and H-36 in 1,3-diaxial orientation. Similarly, the chair conformation of the ring from C-43 to C-47 with the axial orientations of H-43 and H-45 was supported by the NOE correlations between H-43/H-45, H-43/H-48 and H-45/H-48.

**Figure 4:**
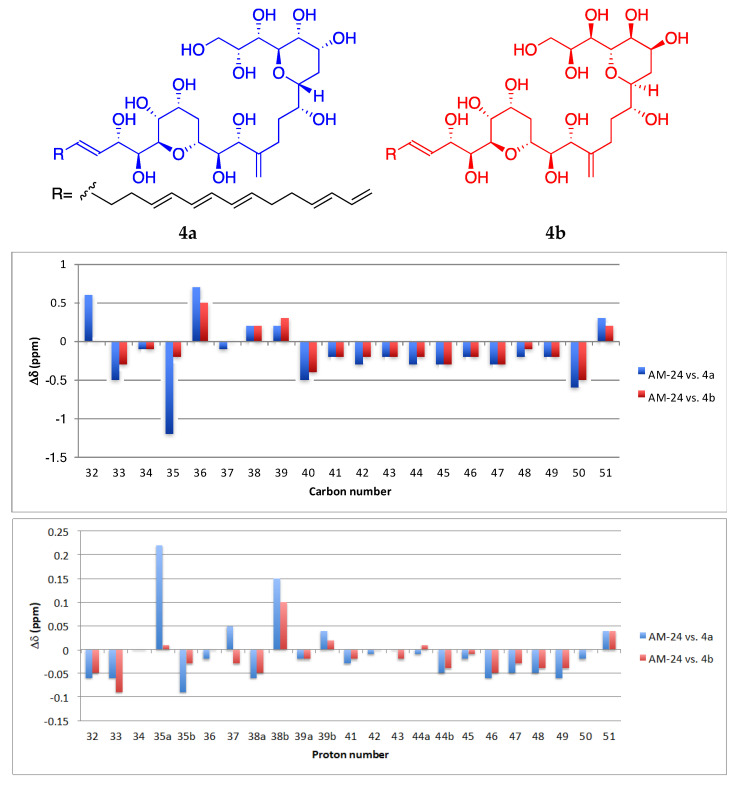
Comparative analysis of NMR data in 2:1 CD_3_OD/C_5_D_5_N for fragment C-30→C-49 between the chemical shifts of AM24 (**1**) and those from compounds **4a** and **4b** synthesized by Wakamiya et al. [22].

Interestingly, the central section C-30→C-49, including the two tetrahydropyran rings, was identical to the central core of AM3. Recently, Wakamiya et al. [22] revised the absolute configuration of AM3 by comparing the NMR data between the natural product and the synthetic model compounds **4a** and **4b**. To establish the relative configuration of the common substructure in compound **1**, a comparative analysis of the NMR chemical shifts in 2:1 CD_3_OD/C_5_D_5_N (Table S2) with those of **4a** and **4b** was carried out and the results are shown in Figure 4. Larger deviations of the C-30→C-49 portion of **1** with **4a** were observed in both ^1^H and ^13^C chemical shifts, whereas the analysis with **4b** revealed that it had a configuration similar to that of **1**. Therefore, the configurations at C-30→C-34 and C-36 in compound **1** are in plausible concordance with those in the revised AM3, and we propose 30*S*, 31*R*, 32*S*, 33*S*, 34*S*, and 36*S* to have two antipodal tetrahydropyran moieties on a simple carbon chain.

HRESIMS analysis of AM25 (**2**) revealed a molecular formula of C_66_H_115_NaO_33_S_2_ based on the *m/z* 1521.6678 ([M − H]^−^, calcd. 1521.6581, C_66_H_115_NaO_33_S_2_) of the observed peak, in negative mode. The structure of this metabolite was determined by comparison of its spectroscopic data with those of AM24 (**1**); in fact, the NMR spectra indicate that both compounds are closely related (Table 1). AM25 (**2**) differs from **1** mainly in the downfield shifts of the oxygenated methine and methylene assigned to C-62 and C-63 (δ_H_ 4.50; δ_C_ 77.3 and δ_H_ 4.10, 4.26; δ_C_ 69.1 in **2** vs. δ_H_ 3.58; δ_C_ 73.0 and δ_H_ 3.43, 3.48; δ_C_ 67.8 in **1**), which are consistent as the site of attachment of two sulfate groups at the 1,2-polyol terminus of **1** (Figure 1). The fragmentation pattern of **2** corresponds to the proposed arrangement of the sulfate ester linked at C-62 and C-63 groups, indeed all the fragment ions could be assigned (Figure S24).

**Figure 5:**
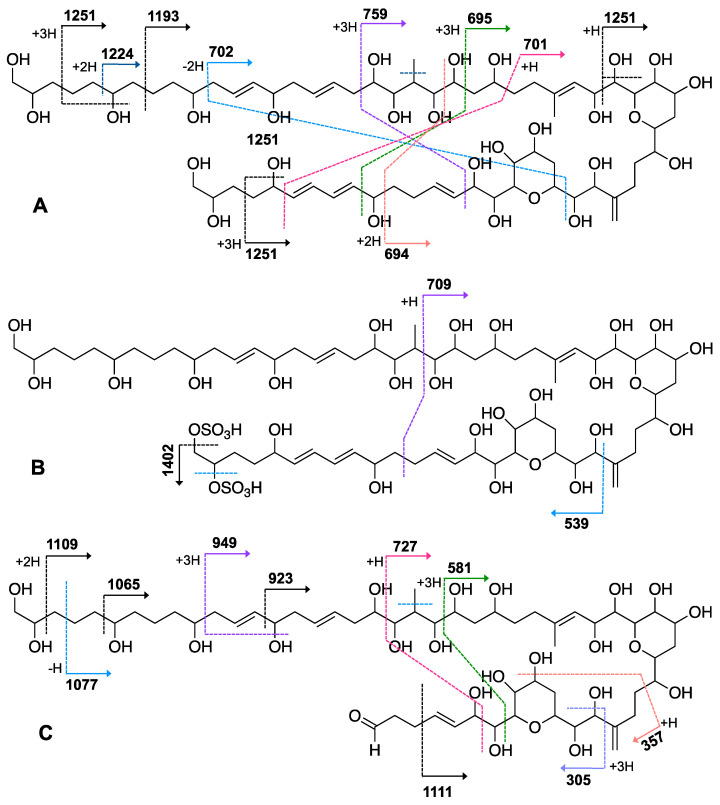
Key mass fragments of AM24 (**1**) (**A**), AM25 (**2**) (**B**), and AM26 (**3**) (**C**).

Compound **3**, AM26, was obtained as an amorphous white solid. The molecular formula was established by HRESIMS as C_57_H_100_O_24_ (*m/z* 1191.6482 [M + Na]^+^; calcd. 1191.6502, C_57_H_100_O_24_Na). Comparison of the ^1^H and ^13^C NMR data of **3** with those of **1** (Table 1) revealed very close similarity in the structures of both compounds sharing the same C-1→C-52 system. The structure of this metabolite was determined by comparison of its spectroscopic data with those of AM24 (**1**) (Table 1). The presence of the characteristic ion peak (Figure 2 and Figure 5 and Figure S33) suggests a similar C-1→C-41 moiety with respect to AMs 24 and 25. The structural difference between **1** and **3** turned out to reside in the terminal C-51→C-63, which was truncated by C-54/C-55. Thus, the main difference in their NMR spectra was the leaking of signals corresponding to fragment C-51→C-63. A ^1^H-^1^H spin system was built for the final fragment of this molecule, but as its carboxylic derivative, since it underwent oxidation during the NMR experiments, a fact that was confirmed by MS (Figures S33 and S34) giving rise to AM27 (**5**) (Table S1). Thus, the corresponding Fragment C was constructed from the proton signal H-41 (δ_H_ 4.19; δ_C_ 76.1), which is coupled as being similar to **1** and **2**, and ends in the methylene group H_2_-53 (δ_H_ 2.16 (2H); δ_C_ 38.8). Long-range ^1^H-^13^C connectivities extracted from the HMBC experiment allowed us to connect this substructure within the rest of the molecule and with the carboxylic carbon at 182.8 (C-54). The close similarity in the ^1^H and ^13^C NMR shifts of the common part C-1→C-49 of compounds **1**, **2** and **3** (Table 1) and NOE correlations analysis showed that the relative stereochemistry for the central core C-30→C-49 should be the same as the one revised for AM3 [22]. 

As part of our ongoing study into the technical feasibility of producing and recovering AMs from pilot-scale photobioreactor cultures of *Amphidinium carterae*, three new amphidinols, AMs 24–26, together with luteophanol D and AM20B were isolated from the remaining supernatant in the continuous clarifying centrifugal separator system. Compared to the 78 L supernatant [6], the yields of the luteophanol D and AM20B were higher, while lingshuiol A was undetected. Interestingly, these findings may be the result of at least two scenarios. The first of them would correspond to a possible breakage of cells by excess centrifugation treatment and the release of intracellular AMs to the supernatant. Although it cannot be dismissed, it is unlikely that lysis was as significant, as centrifugation conditions to prevent it were selected on the basis of a previous study [23].

Discarding cell breakage as the main cause responsible, the second scenario, more likely from our point of view, would point to centrifugation treatment intensity as a stress factor responsible for stimulating cell secretion intensity and altering the profile of secreted AMs. The rationale behind this is related to the well-known flow pattern associated with a tubular centrifuge like that used in our work [24]. Briefly, the feed (i.e., culture) flow inside the centrifuge actually takes place in an inner, much smaller annulus just below the outer stagnant liquid annulus. The depth of this moving, or boundary, layer is thin, yet relatively constant in thickness along the axial direction of the centrifuge. The moving layer is in contact with the more stagnant quiescent thicker layer which occupies almost the entire annular pool. This rotating stagnant supernatant pool has little interaction with the moving layer. In turn, this layer accumulates the cells that settle on the bowl surface forming the pellet. Given the low g-force used in our study (1000 g), the consistency of the pellet was similar to that of a mud. The time that the cells remain in this pellet is variable: from 0 h for the last sedimented cells, to approximately 7 h for the first ones (feed flow = 12–13 L/h). It is evident that the environment of these cells maintained for hours was really stressful: (i) pelleted cells are without access to nutrients and CO_2_ and (ii) continuously subjected to hydrodynamic stress. Under these conditions, it is quite risky to ensure that the stagnant supernatant pool harvested had an AMs profile similar to that detected in the 78 L supernatant. These are conditions that on the scale of hours can stimulate the excretion of some AMs to the detriment of others (no excretion during the culture) to the liquid surrounding the cells pellet in the mud, even leading to the oxidation or metabolization of the AMs initially present in the stagnant liquid pool. It is well documented that excess shear forces can boost the production of polyketides by dinoflagellates [25,26]. These newly synthesized AMs within the centrifugal separator might be mixed with the remainder of the supernatant in the bowl as a consequence of the turbulence generated in the liquid during the deceleration of the centrifuge bowl until it stops.

Although the excretion of polyketides by dinoflagellates into the culture medium has scarcely been studied, it is not a new matter. Intriguingly, published information is diverse. In terms of intracellular versus extracellular compounds, and depending on the growth phase, some of the studies reported almost perfectly mirror each other. In contrast, other polyketides are excreted in amounts that can be up to nine times higher than those recovered from the cell pellet [27,28]. In a few cases, specific polyketides were not detected in the cells (probably due to its low concentration), but in the supernatant [29,30]. In the case of AMs, data reported in the literature refer to compounds extracted from biomass pellets. For example, the concentration of cellular luteophanol D in a 750 L culture of *Amphidinium* sp. is as low as 1.2 μg L^−1^ [20]; 400-fold times lower than that obtained in our previously analyzed supernatant of 78 L [6]. On the other hand, a recent study revealed that the AM profile and cell quotas of eight *Amphidinium* strains were extraordinarily diverse [14]. In that study, lingshuiol A (3 fg cell^−1^) and luteophanol D (<1 fg cell^−1^) were detected in cells of the strain ACRN03 (the same strain as in the study presented here), but at a trace level (near the limit of detection). Meanwhile, other strains could either accumulate cell quotas as high as up to lingshuiol A 1876 fg cell^−1^ and luteophanol D 131 fg cell^−1^, or did not present any trace of AMs. 

### 2.2. Hemolytic Activity

The membrane disrupting and permeabilizing capabilities of AMs have been systematically studied, since these metabolites elicit potent hemolytic and antifungal activities [2]. AMs are singular marine natural products that are active on cell membranes via pore formation in a sterol-dependent manner [18,31,32]. These pores do not obey a cylindrical geometry or symmetry, but they possess a polymorphic nature, which depends on the different AM concentration ranges. Thereby, pore diameters can reach ~10 nm at the surface, measured by atomic force microscopy (AFM) [33], and an estimated inner diameter between 2.0–2.9 nm that can reach 4 nm, according to results obtained from osmotic protection experiments on erythrocytes [34] and conductance tests [33]. 

Although the mode of action of AMs has not been fully elucidated, it is currently accepted that AMs act on the 3-OH beta groups of sterols exposed to extracellular media, leading to a stable complexation as a key step for subsequent insertion into membranes [35]. Evidence on specific molecular recognition has shown that the interaction occurs in the core region, delimited by the tetrahydropyran rings, and involving some atoms beyond [36]. This hyper-conserved structural motif in AMs turns and folds the molecule itself, which is stabilized by intramolecular hydrogen bonds, thus conferring the molecule the characteristic hairpin conformation implied in the selective sterol recognition [37,38,39,40]. There are two main hypothetical models for the formation of AMs channels in the membrane: the barrel stave model, in which AMs are stabilized when embedded in the membranes by self-assembly, forming a pore, and the toroidal model, in which the polyene moiety of AMs interacts with the lipid bulk of membranes, whereas the polar fragments (polyhydroxylated section) do so with water and phosphocholine on the outer side. Furthermore, Iwamoto et al. [33] proposed that both molecular models coexist at all concentration ranges, suggesting morphological transitions between smaller barrel-stave type channels at low AM concentrations, and jumbo pores with a toroidal nature at higher concentrations. These characteristic and unique features make AMs attractive candidates for antimycotic drug development and as hemolytic agents, with AM3 being the most active analogue, often used as a model in both cases [35].

Thus, AMs 24–26 (**1**–**3**) and the previously reported luteophanol D and AM20B were evaluated against bovine and *Sparus aurata* erythrocytes using the methodology described by Eschbach et al. [41]. No hemolytic effects were observed in erythrocytes from defibrinated sheep blood and gilthead sea bream *Sparus aurata* at concentrations below 10 µM and 128 µM (13.4 and 171.5 µg mL^−1^ for AM24), respectively. This lack of activity proves that other structural features participate in poration besides sterol complexation. In this sense, two main aspects were considered: cellular structures (membrane constituents) and variable molecular motifs in AMs. It has been shown that AM activity is enhanced by the presence of transmembrane glycophorin A (GpA), protein especially abundant in erythrocytes and the target for the interaction of some peptidic toxins such as alpha-hemolysin [42]. Several studies on these proteins have proved the affinity of AMs to the GpA transmembrane domain, being able to dissociate protein oligomers linked at that point. Other participative structures on cell membranes like glycolipids have been proposed for consideration [13,35].

The lack of hemolytic activity of compounds **1**–**3** can be explained based on their structural motifs in comparison with the structure–activity relationship studies of several known analogues. It was observed that the hemolytic effects were not influenced, up to a point, by differences in the length and the structure of the polyhydroxyl initial chain among these families of compounds [43,44]. In contrast, they are dramatically affected by the hydrophobicity of the polyene chain of some AMs. A direct interaction between polyolefins and lipid bulk in membranes has been pointed out as being a crucial step for pore formation [34,36,45]. An analysis of the CLog P of the terminal carbon chains of the new compounds versus AM3 reveals important differences in lipophilicity (Table 2), since luteophanol D, AMs 20B, 24 (**1**) and 25 (**2**) contain hydroxyl groups with a diene portion instead of a conjugated triene. The importance of the C-polyene chain for biological efficacy has been illustrated for the case of luteophanol A, which shares the same polyhydroxy chain as luteophanol D (CLogP 0.44), containing two hydroxyl groups, in contrast to AM3 (CLogP 4.32), and showing no hemolytic activity [46]. The presence of highly hydroxylated branches in the new compounds must drastically reduce the interaction with GpA [42] and their capacity for membrane permeabilization. As a consequence, no hemolytic effects were observed [46]. The hemolytic activity depletion may be due to poration inability, since the polyene puncture is involved in earlier steps [34,40]. Furthermore, AM24 (**1**) shows the additional negative effect of the replacement of the terminal vinyl fragment by hydroxyl groups [43]. In the case of AM25 (**2**), the substitution is for a disulfate ester group, which was observed to always result in reduced activity [47]. Finally, the absence of hemolytic activity for AM26 (**3**) (or AM27 (**5**)) as a direct consequence of the truncated polyene chain is also in complete agreement with its increasing polarity (CLopP −1.20) (Table 2). In conclusion, the lack of activity of the molecules reported in this work can be correlated with their highly hydroxylated structures, additional presence of sulfated groups or the shortening of the crucial amphipathic polyenic terminus. 

## 3. Materials and Methods

### 3.1. General Experimental Procedures

Optical rotations were measured on a Perkin–Elmer 241 polarimeter (Waltham, MA, USA) equipped with a sodium lamp. IR spectra were recorded on a Bruker IFS55 spectrophotometer (Ettlingen, Germany) using methanolic solutions over NaCl disk. UV spectra were acquired on a Jasco V-560 spectrophotometer (Easton, MD, USA). NMR spectra were recorded on a Bruker AVANCE 600 MHz instrument (Karlsruhe, Germany) equipped with a 5-mm TCI (Triple Resonance CryoProbe) inverse detection cryo-probe. ^1^H and ^13^C NMR chemical shifts were reported in ppm and referenced to internal residual solvent CD_3_OD at 300 K (δ_H_ 3.31 ppm; δ_C_ 49.0 ppm). NMR experiments were performed using standard pulse sequences. NMR data were processed using Topspin or MestReNova software (v.10., Santiago de Compostela, Spain). Mass spectra were recorded on a LCT Premier XE Micromass spectrometer (Waters, Milford, CT, USA) and on a Waters Acquity H Class UHPLC with Q-Tof LCT Premier XE System (Waters, Milford, CT, USA). HPLC (High-performance liquid chromatography) separations were carried out with a Water system (Waters, Milford, CT, USA) equipped with a Binary HPLC Pump 1525 and Photodiode Array Detector 2996. All of the solvents used were HPLC-grade. Chromatography was monitored by TLC, performed on Silica gel Merck 60 F254. TLC (thin layer chromatography) (Merck, Darmstadt, Germany) plates were visualized using UV light (365 nm) and 10 wt% phosphomolybdic acid solution in methanol.

### 3.2. Biological Material

*Amphidinium carterae*, strain ACRN03, was the marine dinoflagellate microalga used. Cells were obtained from the Culture Collection of Harmful Microalgae at the IEO (Vigo, Spain). The inoculum for the photobioreactor was grown indoors under artificial light (60 μmol photons m^−2^ s^−1^ light flux at the vessel’s surface) in flasks at 21 ± 1 °C under a 12:12 h light–dark cycle. Illumination was supplied by four 58 W fluorescent lamps. The culture medium consisted of filter-sterilized (0.22 μm Millipore filter; Millipore Corporation, Billerica, MA, USA) modified K medium [48] prepared in Mediterranean seawater. The modified K medium composition was: NaNO_3_, 882 μM; NH_4_Cl, 50 μM; NaH_2_PO_4_, 10 μM; TRIS, 1 mM; Na_2_EDTA·2H_2_O, 90 μM; Fe-Na-EDTA, 14.6 μM; MnCl_2_·4H_2_O, 0.9 μM; ZnSO_4_·7H_2_O, 0.08 μM; CoSO_4_·7H_2_O, 0.05 μM; Na_2_MoO_4_·2H_2_O, 0.03 μM; H_2_SeO_3_, 0.01 μM; thiamine, 0.7 μM; biotin, 2.1 nM; B12, 0.37 nM.

### 3.3. Cultivation in the LED-Based Bubble Column PBR

**Figure 6:**
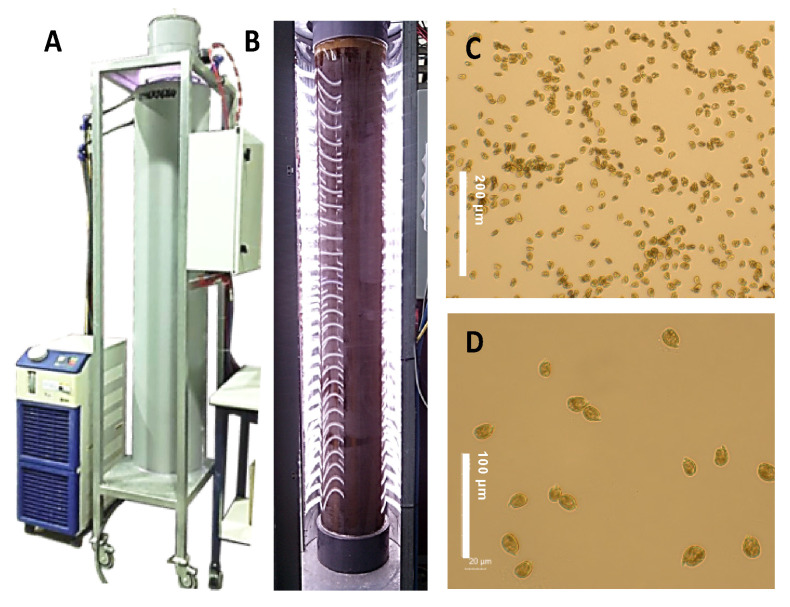
Pilot-scale bubble column photobioreactor system used in obtaining the data presented (**A**). Details of the illumination system based on strips of multicolor light-emission diodes (LEDs) (**B**). Optical microscope images of living cells of *Amphidinium carterae* ACRN03 taken at 20X (scale bar = 200 μm) (**C**) and 40X (scale bar = 100 μm) (**D**) magnification.

*Amphidinium carterae* ACRN03 was photoautotrophically cultured in an LED-illuminated bubble column PBR (Figure 6) as previously described [6]. Briefly, the air flow rate remained below 0.06 vvm to ensure freedom from damaging levels of hydrodynamic stress. Illumination was provided by multicolor LED strips (red, green, blue and warm white, collectively referred to as RGBG; Edison Opto Co., Taiwan) attached horizontally to the insides of two semicircular reflective plastic (PVC) covers that surrounded the PBR. A sinusoidal diel variation pattern was imposed in which the maximum irradiance occurring at midday was fixed at 1500 μmol photons m^−2^ s^−1^. Additional details of the culture system have been reported previously [49]. The culture temperature was controlled at 21 ± 1 °C and the pH was controlled at pH 8.5 by automatically injecting carbon dioxide, as needed. The modified K medium was prepared using filter-sterilized Mediterranean seawater. The medium (65 L) was inoculated with 15 L of an inoculum containing microalgal cells in the late exponential growth phase. The cell concentration in the freshly inoculated photobioreactor was around 30,000 cells mL^−1^. The PBR was operated in fed-batch mode with a pulse feeding strategy. In this procedure, repeated medium replacement was performed every time a stationary growth phase appeared. This replacement consisted of removing 2 L of the broth and replacing it with an equal volume containing a nutrient stock equivalent to 80 L of the modified K medium. Once pulses of nutrient stock did not increase the cell concentration, a stationary growth phase was maintained for 10 days by adding small amounts of nutrient stock (equivalent to 8 L of modified K medium) to compensate the nutritional requirements of basal metabolism. 

### 3.4. Extraction and Chromatographic Separation

**Figure 7:**
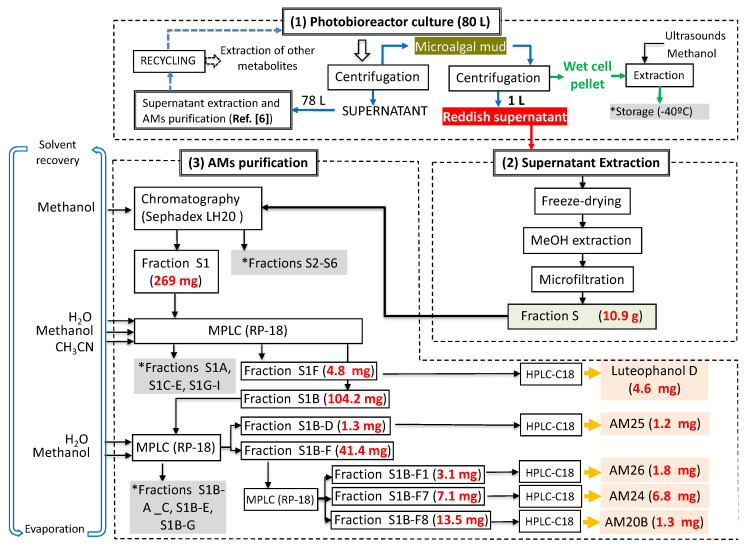
Production of new AM analogues by the marine microalga *Amphidinium carterae* grown in a pilot-scale LED-illuminated photobioreactor.

At the end of the culture period, continuous centrifugation (RINA, model 100M/200M, Spain) operated at 1000× *g* and fed with a broth flow rate as 13 L h^−1^ was applied to separate the microalga from the culture medium. The culture of nearly 80 L provided slightly more than 1 L of microalgal mud and about 78 L of supernatant. Then, in a second centrifugation step (benchtop centrifuge, model SIGMA 4-15C, 2000× *g*), the wet biomass pellet was separated from the microalgal mud and a reddish supernatant recovered (1 L). This supernatant was lyophilized and extracted with methanol yielding, after filtration and solvent removal, a viscous dark green residue of 10.9 g (Figure 7). This work was focused on the AMs present in the supernatant siphoned off. 

The extract (AC03 Fraction S) was subjected to a gel filtration using Sephadex LH-20 (65 × 275 mm) eluted with methanol obtaining six fractions. The first fraction (269 mg) was initially separated by a medium pressure reverse phase LC Lobar LiChroprep RP-18 column (25 × 310 mm) using a stepped gradient (52 min) from MeOH:CH_3_CN:H_2_O (1:2:7) to 100% CH_3_CN at 3 mL min^−1^ to yield nine new fractions S1A-S1I (see Supplementary Material, Scheme S2). The fraction S1B (104.2 mg) was rechromatographed in the above column using MeOH:H_2_O (3:17 to 1:0, 140 min) at 2 mL min^−1^. Fraction S1B–D (1.3 mg) was further purified by HPLC on a Water μ-Bondapack C18 column (19 × 150 mm) eluted with MeOH:H_2_O (3:17 to 1:0, 90 min), 1 mL min^−1^ to afford pure AM25 (**2**, 1.2 mg, *t_R_* = 56.5 min), whereas the fraction S1B–F (41.1 mg) was purified first by a medium pressure reverse phase (Lobar LiChroprep RP-18 column, 25 × 310 mm) with a gradient mobile phase (1:2:7 to 1:0:0 MeOH:CH_3_CN:H_2_O over 155 min, 2 mL min^−1^) to afford crude fractions of AM26 and AM24. Each fraction was subsequent final purified using HPLC (μ-Bondapack C18 column, 19 × 150 mm), with a gradient (1:1 to 8:2 MeOH:H_2_O (0.05%AcOH), 140 min, 1 mL min^−1^) and isocratic (MeOH:CH_3_CN:H_2_O, 1:2:7, 1 mL min^−1^) mobile phase, respectively, to yield AM26 (**3**, 1.8 mg, *t_R_* = 34.0 min) and AM24 (**1**, 6.8 mg, *t_R_* = 18.0 min). Known compounds luteophanol D (4.6 mg) and AM20B (1.3 mg) were also isolated from the fractions S1F and S1B–F, respectively (Figure 1, Scheme S2). Lingshuiol A was not detected in this study.

*Amphidinol 24* (**1**): Yellow oil; [α]^25^_D_ +13 (*c* 0.09, MeOH); UV (MeOH) λ_max_ 230 nm (ε 27123); IR ν_max_ 3264, 2931, 2366, 2345, 2034, 2011, 1978, 1608 and 1020 cm^−1^; ^1^H and ^13^C NMR data (CD_3_OD) see Table 1; HRESIMS *m/z* 1363.7606 [M + Na]^+^ (calcd. 1363.7602 for C_66_H_116_O_27_Na). 

*Amphidinol 25* (**2**): Yellow oil; [α]^25^_D_ +1 (*c* 0.13, MeOH); IR ν_max_ 3266, 2940, 2867, 2364, 2167, 2034, 1976, 1614 and 1022 cm^−1^; ^1^H and ^13^C NMR data (CD_3_OD) see Table 1; HRESIMS *m/z* 1521.6678 [M − H]^−^ (calcd. 1521.6581 for C_66_H_114_O_33_NaS_2_).

*Amphidinol 26* (**3**): Colorless amorphous solid; [α]^25^_D_ −30 (*c* 0.13, MeOH); IR ν_max_ 3262, 2832, 2366, 2167, 2034, 1976, 1613 and 1022 cm^−1^; ^1^H and ^13^C NMR data (CD_3_OD) see Table 1; HRESIMS *m/z* 1191.6482 [M + Na]^+^ (calcd. 1191.6502 for C_57_H_100_O_24_Na).

The known compounds luteophanol D and amphidinol 20B were identified by detailed analysis of the NMR and MS spectrometric data (Figures S36–S39) and comparison with those reported in the literature [6,20].

### 3.5. Hemolytic Assays

Erythrocyte lysis assay was performed as described elsewhere [41]. Erythrocytes from defibrinated sheep blood and from gilt-head (sea) bream (*Sparus aurata*) grown in a fish farm (blood was collected by caudal vein puncture). Serial methanolic dilutions of AMs 24–26 (**1**–**3**), luteophanol D and AM20B were placed in a microwell and air dried. The concentrations of AMs in microwells ranged from 0 to 5.5 × 10^4^ ng mL^−1^. A erythrocyte concentration of 45 × 10^6^ cells per well was used. Negative controls consisted of erythrocytes incubated in Mediterranean seawater. Positive control, i.e., 100% hemolysis, was obtained using distilled water. The dose–response curves (percentage of hemolysis (*PH*) vs. log of number of *A*. *carterae* cells per well (*x*)) were interpreted in terms of the Hill Equation (1):

(1) PH=PHmin+PHmax−PHmin1+(xEC50)η

where *PH* is the percentage of hemolysis; *x* is the concentration of AM per well; *PH_max_* represents the maximum percentage of hemolysis equal to 100%; EC_50_ is the concentration of AM per well giving 50% hemolysis and *η* is the Hill slope. Saponin (Sigma Aldrich, 47036, CAS nº 8047-15-2, Saint Louis, MO, USA), was used as a positive control obtaining an EC_50_ value of 10.7 × 10^6^ ± 1.06 × 10^6^ pg per well through Hill equation. An equivalent saponin potency (pg per AM pg) was calculated by dividing the EC_50_ for saponin by the EC_50_ for AM.

## 4. Conclusions

Three new related amphidinol analogues, named amphidinol 24, amphidinol 25 and amphidinol 26, were obtained from the marine dinoflagellate *Amphidinium carterae*, successfully cultured in a pilot-scale bubble column photobioreactor illuminated with multicolor light-emitting diodes (LEDs) operated in fed-batch mode with a pulse feeding strategy. The structures were established by extensive spectroscopic methods, while the relative configurations of the C16 common central core (C-32→C-47) were determined by comparison of the NMR data of AM24 with those of two synthetic intermediates of AM3 [22]. The results confirm that both tetrahydropyran counterparts exist as antipodal moieties on a single carbon chain, in accordance with that unique structural aspect of the recently revised configuration of AM3. 

A structure–activity relationship study of the new metabolites and other related derivatives against hemolytic activity was carried out, and it was observed that they are dramatically affected by the hydrophobicity (rationalized in the form of log P) of the polyene chain. The lack of activity of the molecules concerned can be correlated with their highly hydroxylated structures, additional presence of sulfated groups, or the shortening of the crucial amphipathic polyenic terminus. 

This work reveals, in terms of acclimation, growth rates, biomass productivity, downstream processing and excellent recovery yields of excreted AM analogs, a useful bioprocess strategy that may be adaptable to a suitable production of other super carbon chain compounds or biotoxins from marine dinoflagellates.

## Figures and Tables

**Table 1:** NMR data for AMs 24–26 (compounds **1**–**3**) (600 MHz; 300 °K, CD_3_OD).

	AM24 (1)		AM25 (2)		AM26 (3)	
Carbon nº	δ_C_, Type	δ_H_	δ_C_, Type	δ_H_	δ_C_, Type	δ_H_
**1**	67.0, CH_2_	3.43; 3.48	67.1, CH_2_	3.43; 3.47	67.1, CH_2_	3.43; 3.48
**2**	73.0, CH	3.58	73.1, CH	3.59	73.1, CH	3.59
**3**	34.2, CH_2_	1.38; 1.54	34.3, CH_2_	1.37; 1.54	34.3, CH_2_	1.38; 1.54
**4**	22.6, CH_2_	1.38; 1.62	22.6, CH_2_	1.38; 1.61	22.6, CH_2_	1.38; 1.61
**5**	38.2, CH_2_	1.40; 1.50	38.2, CH_2_	1.40; 1.50	38.1, CH_2_	1.40; 1.50
**6**	72.0, CH	3.54	72.1, CH	3.54	72.0, CH	3.56
**7**	38.2, CH_2_	1.40; 1.50	38.2, CH_2_	1.40; 1.50	38.1, CH_2_	1.40; 1.50
**8**	22.6, CH_2_	1.38; 1.62	22.6, CH_2_	1.38; 1.61	22.6, CH_2_	1.38; 1.62
**9**	37.6, CH_2_	1.40; 1.52	37.6, CH_2_	1.39; 1.52	37.7, CH_2_	1.40; 1.52
**10**	71.9, CH	3.58	72.2, CH	3.58	72.4, CH	3.59
**11**	41.2, CH_2_	2.20 (2H)	41.4, CH_2_	2.20 (2H)	41.2, CH_2_	2.19 (2H)
**12**	128.6, CH	5.69	128.6, CH	5.68	128.5, CH	5.70
**13**	136.0, CH	5.53	135.9, CH	5.53	135.9, CH	5.55
**14**	73.2, CH	4.05	73.3, CH	4.05	73.2, CH	4.05
**15**	41.7, CH_2_	2.25 (2H)	41.8, CH_2_	2.24 (2H)	41.7, CH_2_	2.24 (2H)
**16**	129.7, CH	5.54	129.6, CH	5.53	129.6, CH	5.55
**17**	137.3, CH	5.60	130.1, CH	5.60	130.1, CH	5.60
**18**	37.7, CH_2_	2.08; 2.48	37.7, CH_2_	2.08; 2.48	37.7, CH_2_	2.08; 2.48
**19**	72.2, CH	3.52	72.2, CH	3.52	72.1, CH	3.52
**20**	78.9, CH	3.52	78.7, CH	3.52	78.7, CH	3.52
**21**	35.0, CH	2.30	35.0, CH	2.30	34.9, CH	2.30
**22**	79.9, CH	3.53	79.6, CH	3.53	79.7, CH	3.53
**23**	71.7, CH	3.71	71.2, CH	3.71	71.7, CH	3.72
**24**	40.7, CH_2_	1.54; 1.91	40.9, CH_2_	1.53; 1.91	40.8, CH_2_	1.54; 1.90
**25**	71.1, CH	3.86	71.1, CH	3.86	70.1, CH	3.87
**26**	36.2, CH_2_	1.59; 1.68	37.4, CH_2_	1.59; 1.68	36.2, CH_2_	1.59; 1.68
**27**	36.8, CH_2_	2.12; 2.21	36.5, CH_2_	2.12; 2.21	36.4, CH_2_	1.54; 1.90
**28**	139.0, C		139.0, C		139.1, C	
**29**	125.9, CH	5.48	125.9, CH	5.48	125.8, CH	5.48
**30**	67.6, CH	4.55	67.6, CH	4.55	67.6, CH	4.56
**31**	72.0, CH	3.69	72.0, CH	3.69	72.0, CH	3.68
**32**	78.8, CH	3.96	78.9, CH	3.97	78.8, CH	3.96
**33**	67.1, CH	3.97	68.4, CH	4.04	68.4, CH	4.05
**34**	68.4, CH	4.04	68.4, CH	3.97	67.1, CH	3.98
**35**	30.0, CH_2_	1.79 (2H)	30.1, CH_2_	1.79 (2H)	30.1, CH_2_	1.79 (2H)
**36**	75.3, CH	3.49	75.3, CH	3.49	75.3, CH	3.49
**37**	74.2, CH	3.60	74.1, CH	3.60	74.1, CH	3.61
**38**	32.1, CH_2_	1.57; 1.97	32.3 CH_2_	1.57; 1.97	32.2 CH_2_	1.56; 1.97
**39**	27.8, CH_2_	2.10; 2.42	27.9, CH_2_	2.10; 2.42	28.0, CH_2_	2.10; 2.41
**40**	151.4, C		151.1, C		151.2, C	
**41**	76.3, CH	4.18	76.2, CH	4.18	76.1, CH	4.19
**42**	74.1, CH	3.35	75.0, CH	3.34	75.0, CH	3.35
**43**	70.0, CH	4.05	70.1, CH	4.04	70.2, CH	4.04
**44**	31.1 CH_2_	1.56; 2.09	31.3, CH_2_	1.56; 2.09	31.2, CH_2_	1.56; 2.09
**45**	66.8, CH	4.05	67.1, CH	4.05	67.2, CH	4.05
**46**	68.4, CH	4.05	68.4, CH	4.04	68.4, CH	4.05
**47**	80.2, CH	3.74	80.3, CH	3.75	80.1, CH	3.75
**48**	71.6, CH	3.97	71.7, CH	3.96	71.6, CH	3.97
**49**	73.8, CH	4.37	73.9, CH	4.36	73.7, CH	4.37
**50**	128.6, CH	5.64	128.6, CH	5.63	128.5, CH	5.66
**51**	134.9, CH	5.80	135.0, CH	5.80	134.7, CH	5.83
**52**	29.3, CH_2_	2.16 (2H)	29.4, CH_2_	2.15 (2H)	29.4, CH_2_	2.18 (2H)
**53**	37.6, CH_2_	1.60; 1.64	37.6, CH_2_	1.62 (2H)	38.8 *, CH_2_	2.16 * (2H)
**54**	72.2, CH	4.12	72.4, CH	4.11	182.8 *, C	
**55**	137.0, CH	5.69	133.8, CH	5.67	6.7, CH_3_	0.97
**56**	130.7, CH	6.23	130.7, CH	6.23	17.1, CH_3_	1.75
**57**	130.7, CH	6.23	130.7, CH	6.23	112.6, CH_2_	4.99; 5.09
**58**	137.0, CH	5.69	133.8, CH	5.67		
**59**	72.8, CH	4.10	72.4, CH	4.11		
**60**	34.2, CH_2_	1.59; 1.71	33.7, CH_2_	1.71; 1.73		
**61**	34.2, CH_2_	1.38; 1.54	34.2, CH_2_	1.71; 1.87		
**62**	73.0, CH	3.58	77.3, CH	4.50		
**63**	67.8, CH_2_	3.43; 3.48	69.1, CH_2_	4.10; 4.26		
**64**	6.6, CH_3_	0.98	6.7, CH_3_	0.98		
**65**	17.1, CH_3_	1.75	17.1, CH_3_	1.75		
**66**	112.8, CH_2_	4.99; 5.08	112.7, CH_2_	4.99; 5.08		

* Determined as AM27 (**5**).

**Table 2:** CLog P values for the polyene side chain of AMs identified in *A. carterae* ACRN03 vs. AM3.

Compound	CLog P	Molecular Fragment
AM3	4.32	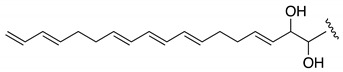
Luteophanol D	0.44	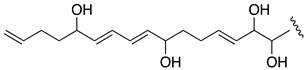
AM20B	−1.23	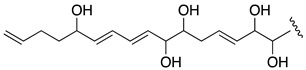
AM24	−2.73	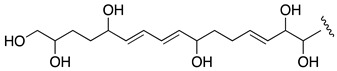
AM25	−2.82	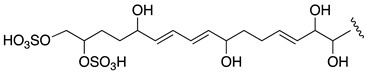
AM26	−1.20	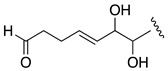
AM27	−1.17	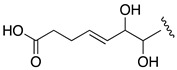

## Data Availability

Data is contained within the article and supplementary material.

## Supplementary Materials

The following are available online at https://www.mdpi.com/article/10.3390/md19080432/s1, Scheme S1: Production of new amphidinol analogues by the marine microalga Amphidinium carterae grown in a pilot-scale LED-illuminated photobioreactor, Scheme S2: Isolation procedure for new amphidinol analogues, Table S1: 1H and 13C NMR data (600 MHz, CD3OD) for amphidinols 24, 25 and 27, Table S2: 1H and 13C NMR data comparison for carbons C-30 → C-51 in CD3OD-C5D5N 2:1 for amphidinol 24 versus related synthetic fragments 4a and 4b reported by Wakamiya et al. [22], Figures S1–S15: 1D and 2D NMR spectra for amphidinol 24, Figure S16: HRESIMS spectrum for amphidinol 24, Figure S17: Main MS/MS fragments observed for amphidinol 24, Figures S18–S22: 1D and 2D NMR spectra for amphidinol 25, Figure S23: HRESIMS spectrum for amphidinol 25, Figure S24: Main MS/MS fragments observed for amphidinol 25, Figures S25–S31: 1D and 2D NMR spectra for amphidinol 26, Figure S32: HRESIMS spectrum for amphidinol 26, Figure S33: Amphidinol 26 conversion from aldehyde to carboxylic acid at C-54 observed by ESI-HRMS, Figure S34: Amphidinol 26 single mass composition analysis for aldehyde and carboxylic states, Figure S35: Main MS/MS fragments observed for amphidinol 26, Figures S36 and S38: 1H NMR and HSQCed spectra of known luteophanol D and amphidinol 20B, Figures S37 and S39: HRESIMS spectra of known luteophanol D and amphidinol 20B.
