# Supplementary material for: Isolation and Structural Elucidation of New Amphidinol Analogues from Amphidinium carterae Cultivated in a Pilot-Scale Photobioreactor
Source: Mar Drugs. 2021 Jul 29;19(8):432. doi: 10.3390/md19080432 (PMC8399002; doi:10.3390/md19080432)
Supplement: Supplementary file 1 [file marinedrugs-19-00432-s001.zip › marinedrugs-1311252-supplementary.pdf]

SUPPLEMENTARY MATERIAL

Isolation and structural elucidation of new amphidinol analogues from *Amphidinium carterae* cultivated in a pilot scale photobioreactor

Adrián Morales-Amador[1,2], Alejandro Molina-Miras[3,4], Lorenzo López-Rosales[3,4], Asterio Sánchez-Mirón[3,4], Francisco García-Camacho[3,4], María L. Souto[1,2]* and José J. Fernández[1,2]*

[1] Instituto Universitario de Bio-Orgánica Antonio González (IUBO AG), Universidad de La Laguna (ULL), Avda. Astrofísico F. Sánchez 2, 38206 La Laguna, Tenerife, Spain

[2] Departamento de Química Orgánica, Universidad de La Laguna (ULL), Avda. Astrofísico F. Sánchez 2, 38206 La Laguna, Tenerife, Spain

[3] Chemical Engineering Department, University of Almería, 04120 Almería, Spain

[4] Research Center CIAIMBITAL, University of Almería, 04120 Almería, Spain

Table of contents

	Description	Page
**Scheme S1**	Production of new amphidinol analogues by the marine microalga *Amphidinium carterae* grown in a pilot-scale LED-illuminated photobioreactor.	S4
**Scheme S2**	Isolation procedure for new amphidinol analogues.	S5
**Table S1**	^{1}H and ^{13}C NMR data (600 MHz, CD_3OD) for amphidinols 24, 25 and 27.	S6
**Figure S1**	^{1}H NMR spectrum (600 MHz, CD_3OD) for amphidinol 24.	S7
**Figure S2**	COSY spectrum (600 MHz, CD_3OD) for amphidinol 24.	S8
**Figure S3**	$HSQC_{ed}$ spectrum (600 MHz, CD_3OD) for amphidinol 24.	S9
**Figure S4**	HSQC-TOCSY spectrum (600 MHz, CD_3OD) for amphidinol 24.	S10
**Figure S5**	HMBC spectrum (600 MHz, CD_3OD) for amphidinol 24.	S11
**Figure S6**	H2BC spectrum (600 MHz, CD_3OD) for amphidinol 24.	S12
**Figure S7**	T-ROESY spectrum (600 MHz, CD_3OD) for amphidinol 24.	S13
**Figure S8**	1D-NOESY spectral (600 MHz, CD_3OD) for amphidinol 24.	S14
**Figure S9**	^{1}H NMR spectrum (600 MHz, CD_3OD-C_5D_5N 2:1) for amphidinol 24.	S15
**Figure S10**	$HSQC_{ed}$ spectrum (600 MHz, CD_3OD-C_5D_5N 2:1) for amphidinol 24.	S16
**Figure S11**	HSQC-TOCSY spectrum (600 MHz, CD_3OD-C_5D_5N 2:1) for amphidinol 24.	S17
**Figure S12**	HMBC spectrum (600 MHz, CD_3OD-C_5D_5N 2:1) for amphidinol 24.	S18
**Figure S13**	H2BC spectrum (600 MHz, CD_3OD-C_5D_5N 2:1) for amphidinol 24.	S19
**Figure S14**	T-ROESY spectrum (600 MHz, CD_3OD-C_5D_5N 2:1) for amphidinol 24.	S20
**Figure S15**	1D-NOESY spectrum (600 MHz, CD_3OD-C_5D_5N 2:1) for amphidinol 24.	S21
**Table S2**	^{1}H and ^{13}C NMR data comparison for carbons C-30 → C-51 in CD_3OD-C_5D_5N 2:1 for amphidinol 24 *versus* related synthetic fragments 4a and 4b reported by Wakamiya et al [22].	S22
**Figure S16**	HRESIMS spectrum for amphidinol 24.	S23
**Figure S17**	Main MS/MS fragments observed for amphidinol 24.	S24
**Figure S18**	^{1}H NMR spectrum (600 MHz, CD_3OD) for amphidinol 25.	S25
**Figure S19**	COSY spectrum (600 MHz, CD_3OD) for amphidinol 25.	S26
**Figure S20**	$HSQC_{ed}$ spectrum (600 MHz, CD_3OD) for amphidinol 25.	S27
**Figure S21**	HSQC-TOCSY spectrum (600 MHz, CD_3OD) for amphidinol 25.	S28

**Figure S22**	HMBC spectrum (600 MHz, CD_3OD) for amphidinol 25.	S29
**Figure S23**	HRESIMS spectrum for amphidinol 25.	S30
**Figure S24**	Main MS/MS fragments observed for amphidinol 25.	S31
**Figure S25**	^{1}H NMR spectrum (600 MHz, CD_3OD) for amphidinol 26.	S32
**Figure S26**	COSY spectrum (600 MHz, CD_3OD) for amphidinol 26.	S33
**Figure S27**	$HSQC_{ed}$ spectrum (600 MHz, CD_3OD) for amphidinol 26.	S34
**Figure S28**	HSQC-TOCSY spectrum (600 MHz, CD_3OD) for amphidinol 26.	S35
**Figure S29**	HMBC spectrum (600 MHz, CD_3OD) for amphidinol 26.	S36
**Figure S30**	H2BC spectrum (600 MHz, CD_3OD) for amphidinol 26.	S37
**Figure S31**	T-ROESY spectrum (600 MHz, CD_3OD) for amphidinol 26.	S38
**Figure S32**	HRESIMS spectrum for amphidinol 26.	S39
**Figure S33**	Amphidinol 26 conversion from aldehyde to carboxylic acid at C-54 observed by ESI-HRMS.	S40
**Figure S34**	Amphidinol 26 single mass composition analysis for aldehyde and carboxylic states.	S41
**Figure S35**	Main MS/MS fragments observed for amphidinol 26.	S42
**Figure S36**	^{1}H NMR and $HSQC_{ed}$ spectra (600 MHz, CD_3OD) for luteophanol D.	S43
**Figure S37**	HRESIMS spectrum for luteophanol D.	S44
**Figure S38**	^{1}H NMR and $HSQC_{ed}$ spectra (600 MHz, CD_3OD) for amphidinol 20B.	S45
**Figure S39**	HRESIMS spectrum for amphidinol 20B.	S46

Scheme S1. Production of new amphidinol analogues by the marine microalga *Amphidinium carterae* grown in a pilot-scale LED-illuminated photobioreactor.

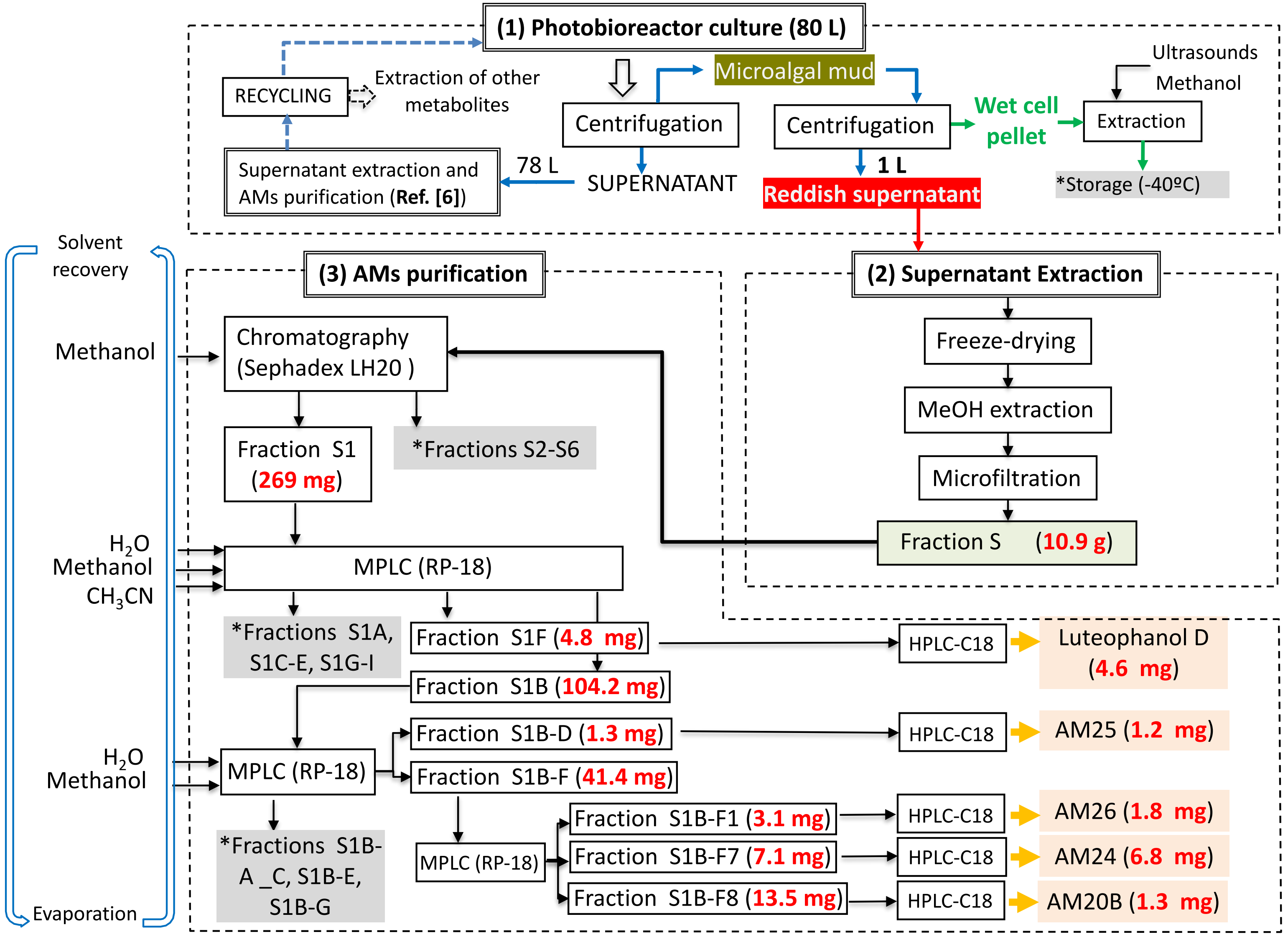

Scheme S2. Isolation procedure for new amphidinol analogues.

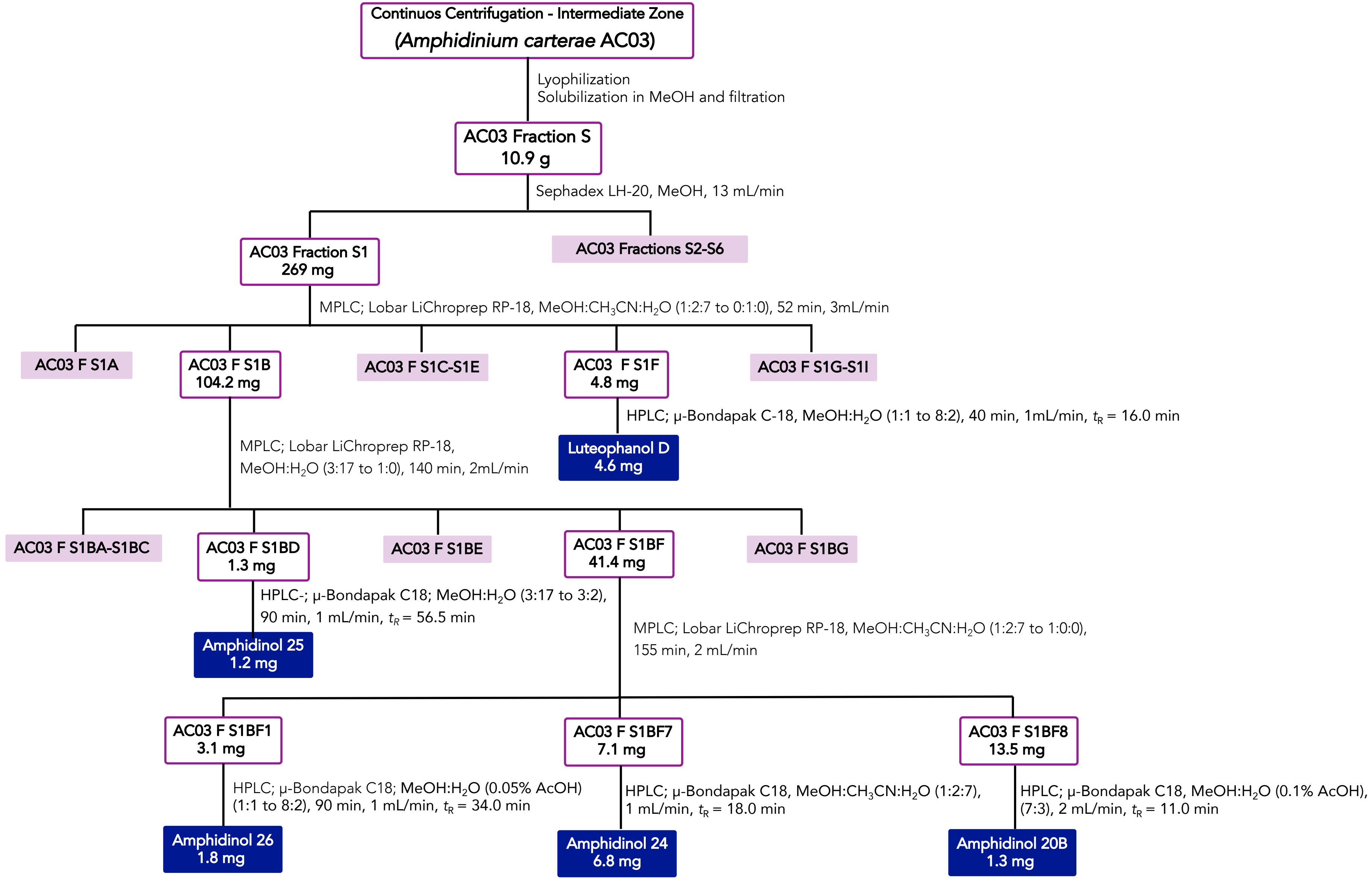

Table S1. ^{1}H and ^{13}C-NMR data (600 MHz, CD_3OD) for amphidinols 24, 25 and 27.

#	Amphidinol 24		Amphidinol 25		Amphidinol 27	
	δC, type	δH	δC, type	δH	δC, type	δH
**1**	67.0, CH_2	3.43; 3.48	67.1, CH_2	3.43; 3.47	67.1, CH_2	3.43; 3.48
**2**	73.0, CH	3.58	73.1, CH	3.59	73.1, CH	3.59
**3**	34.2, CH_2	1.38; 1.54	34.3, CH_2	1.37; 1.54	34.3, CH_2	1.38; 1.54
**4**	22.6, CH_2	1.38; 1.62	22.6, CH_2	1.38; 1.61	22.6, CH_2	1.38; 1.61
**5**	38.2, CH_2	1.40; 1.50	38.2, CH_2	1.40; 1.50	38.1, CH_2	1.40; 1.50
**6**	72.0, CH	3.54	72.1, CH	3.54	72.0, CH	3.56
**7**	38.2, CH_2	1.40; 1.50	38.2, CH_2	1.40; 1.50	38.1, CH_2	1.40; 1.50
**8**	22.6, CH_2	1.38; 1.62	22.6, CH_2	1.38; 1.61	22.6, CH_2	1.38; 1.62
**9**	37.6, CH_2	1.40; 1.52	37.6, CH_2	1.39; 1.52	37.7, CH_2	1.40; 1.52
**10**	71.9, CH	3.58	72.2, CH	3.58	72.4, CH	3.59
**11**	41.2, CH_2	2.20 (2H)	41.4, CH_2	2.20 (2H)	41.2, CH_2	2.19 (2H)
**12**	128.6, CH	5.69	128.6, CH	5.68	128.5, CH	5.70
**13**	136.0, CH	5.53	135.9, CH	5.53	135.9, CH	5.55
**14**	73.2, CH	4.05	73.3, CH	4.05	73.2, CH	4.05
**15**	41.7, CH_2	2.25 (2H)	41.8, CH_2	2.24 (2H)	41.7, CH_2	2.24 (2H)
**16**	129.7, CH	5.54	129.6, CH	5.53	129.6, CH	5.55
**17**	137.3, CH	5.60	130.1, CH	5.60	130.1, CH	5.60
**18**	37.7, CH_2	2.08; 2.48	37.7, CH_2	2.08; 2.48	37.7, CH_2	2.08; 2.48
**19**	72.2, CH	3.52	72.2, CH	3.52	72.1, CH	3.52
**20**	78.9, CH	3.52	78.7, CH	3.52	78.7, CH	3.52
**21**	35.0, CH	2.30	35.0, CH	2.30	34.9, CH	2.30
**22**	79.9, CH	3.53	79.6, CH	3.53	79.7, CH	3.53
**23**	71.7, CH	3.71	71.2, CH	3.71	71.7, CH	3.72
**24**	40.7, CH_2	1.54; 1.91	40.9, CH_2	1.53; 1.91	40.8, CH_2	1.54; 1.90
**25**	71.1, CH	3.86	71.1, CH	3.86	70.1, CH	3.87
**26**	36.2, CH_2	1.59; 1.68	37.4, CH_2	1.59; 1.68	36.2, CH_2	1.59; 1.68
**27**	36.8, CH_2	2.12; 2.21	36.5, CH_2	2.12; 2.21	36.4, CH_2	1.54; 1.90
**28**	139.0, C		139.0, C		139.1, C	
**29**	125.9, CH	5.48	125.9, CH	5.48	125.8, CH	5.48
**30**	67.6, CH	4.55	67.6, CH	4.55	67.6, CH	4.56
**31**	72.0, CH	3.69	72.0, CH	3.69	72.0, CH	3.68
**32**	78.8, CH	3.96	78.9, CH	3.97	78.8, CH	3.96
**33**	67.1, CH	3.97	68.4, CH	4.04	68.4, CH	4.05
**34**	68.4, CH	4.04	68.4, CH	3.97	67.1, CH	3.98
**35**	30.0, CH_2	1.79 (2H)	30.1, CH_2	1.79 (2H)	30.1, CH_2	1.79 (2H)
**36**	75.3, CH	3.49	75.3, CH	3.49	75.3, CH	3.49
**37**	74.2, CH	3.60	74.1, CH	3.60	74.1, CH	3.61
**38**	32.1, CH_2	1.57; 1.97	32.3 CH_2	1.57; 1.97	32.2 CH_2	1.56; 1.97
**39**	27.8, CH_2	2.10; 2.42	27.9, CH_2	2.10; 2.42	28.0, CH_2	2.10; 2.41
**40**	151.4, C		151.1, C		151.2, C	
**41**	76.3, CH	4.18	76.2, CH	4.18	76.1, CH	4.19
**42**	74.1, CH	3.35	75.0, CH	3.34	75.0, CH	3.35
**43**	70.0, CH	4.05	70.1, CH	4.04	70.2, CH	4.04
**44**	31.1 CH_2	1.56; 2.09	31.3, CH_2	1.56; 2.09	31.2, CH_2	1.56; 2.09
**45**	66.8, CH	4.05	67.1, CH	4.05	67.2, CH	4.05
**46**	68.4, CH	4.05	68.4, CH	4.04	68.4, CH	4.05
**47**	80.2, CH	3.74	80.3, CH	3.75	80.1, CH	3.75
**48**	71.6, CH	3.97	71.7, CH	3.96	71.6, CH	3.97
**49**	73.8, CH	4.37	73.9, CH	4.36	73.7, CH	4.37
**50**	128.6, CH	5.64	128.6, CH	5.63	128.5, CH	5.66
**51**	134.9, CH	5.80	135.0, CH	5.80	134.7, CH	5.83
**52**	29.3, CH_2	2.16 (2H)	29.4, CH_2	2.15 (2H)	29.4, CH_2	2.18 (2H)
**53**	37.6, CH_2	1.60; 1.64	37.6, CH_2	1.62 (2H)	38.8, CH	2.16 (2H)
**54**	72.2, CH	4.12	72.4, CH	4.11	182.8, C	
**55**	137.0, CH	5.69	133.8, CH	5.67	6.7, CH_3	0.97
**56**	130.7, CH	6.23	130.7, CH	6.23	17.1, CH_3	1.75
**57**	130.7, CH	6.23	130.7, CH	6.23	112.6, CH_2	4.99; 5.09
**58**	137.0, CH	5.69	133.8, CH	5.67		
**59**	72.8, CH	4.10	72.4, CH	4.11		
**60**	34.2, CH_2	1.59; 1.71	33.7, CH_2	1.71; 1.73		
**61**	34.2, CH_2	1.38; 1.54	34.2, CH_2	1.71; 1.87		
**62**	73.0, CH	3.58	77.3, CH	4.50		
**63**	67.8, CH_2	3.43; 3.48	69.1, CH_2	4.10; 4.26		
**64**	6.6, CH_3	0.98	6.7, CH_3	0.98		
**65**	17.1, CH_3	1.75	17.1, CH_3	1.75		
**66**	112.8, CH_2	4.99; 5.08	112.7, CH_2	4.99; 5.08		

Figure S1. ^{1}H NMR spectrum (600 MHz, CD_3OD) for amphidinol 24.

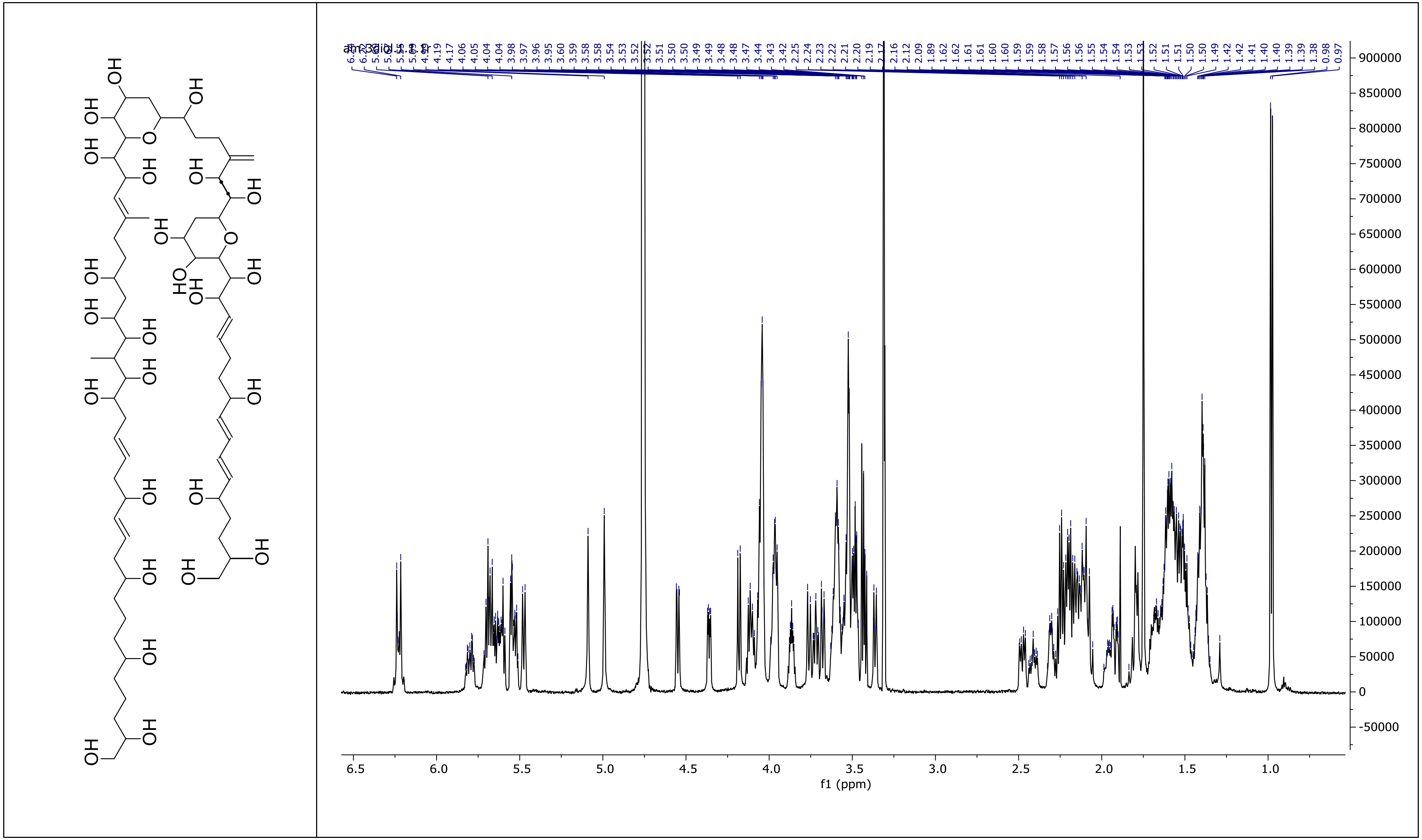

Figure S2. COSY spectrum (600 MHz, CD_3OD) for amphidinol 24.

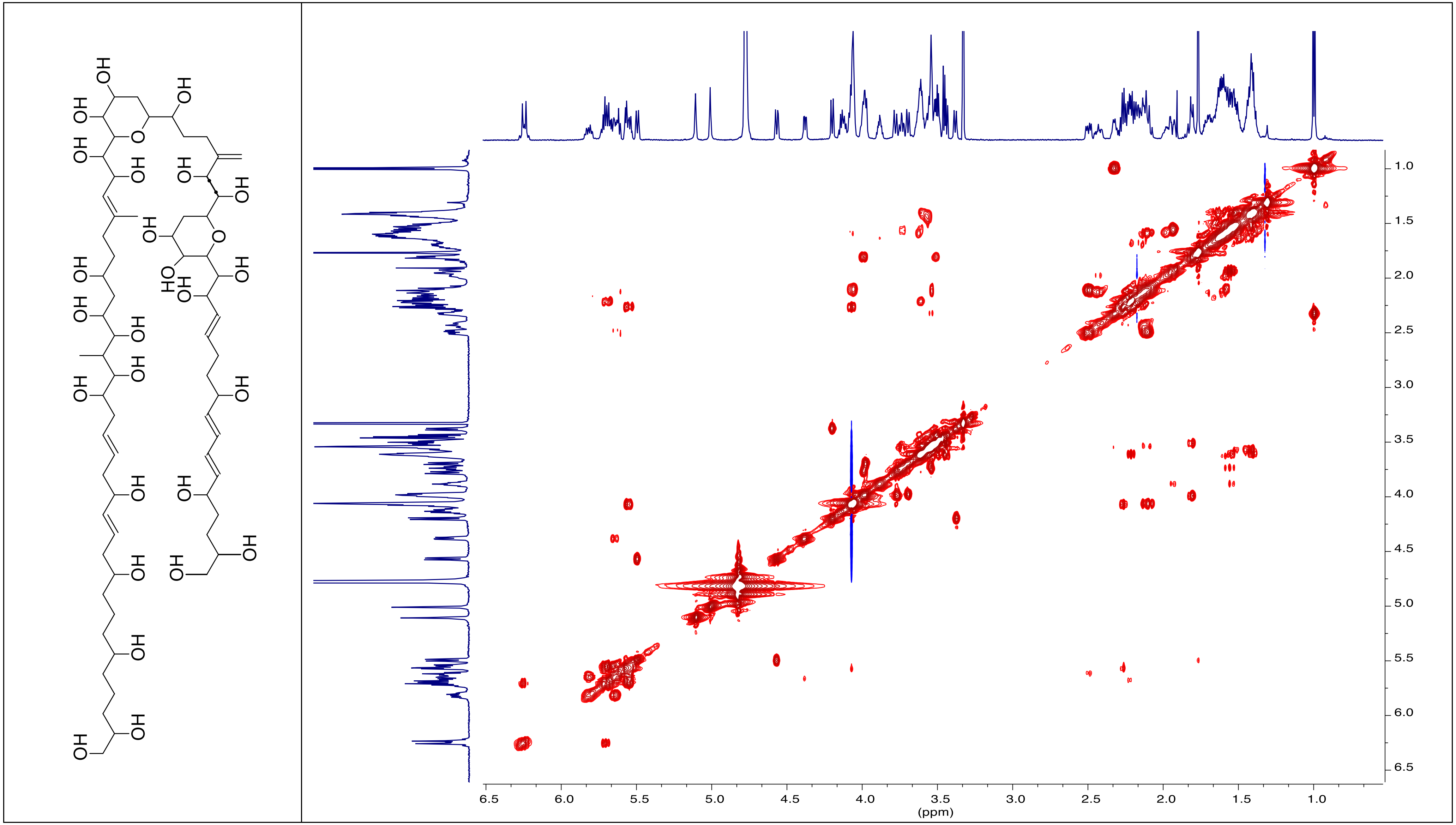

Figure S3. HSQC$_{ed}$ spectrum (600 MHz, CD_3OD) for amphidinol 24.

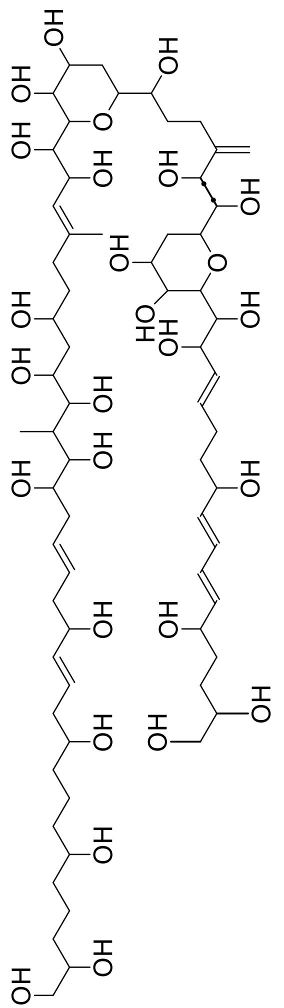

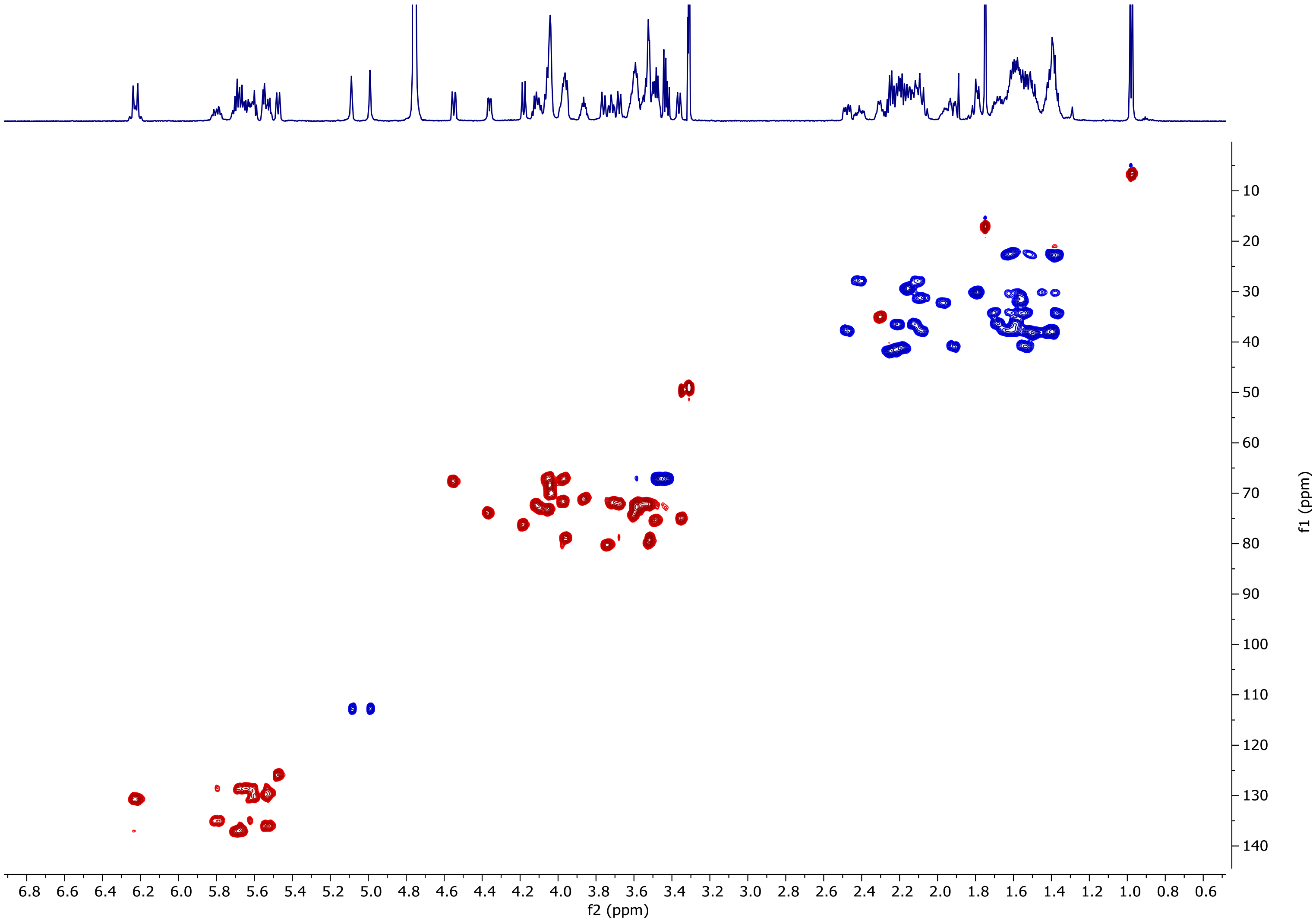

Figure S4. HSQC-TOCSY spectrum (600 MHz, CD_3OD) for amphidinol 24.

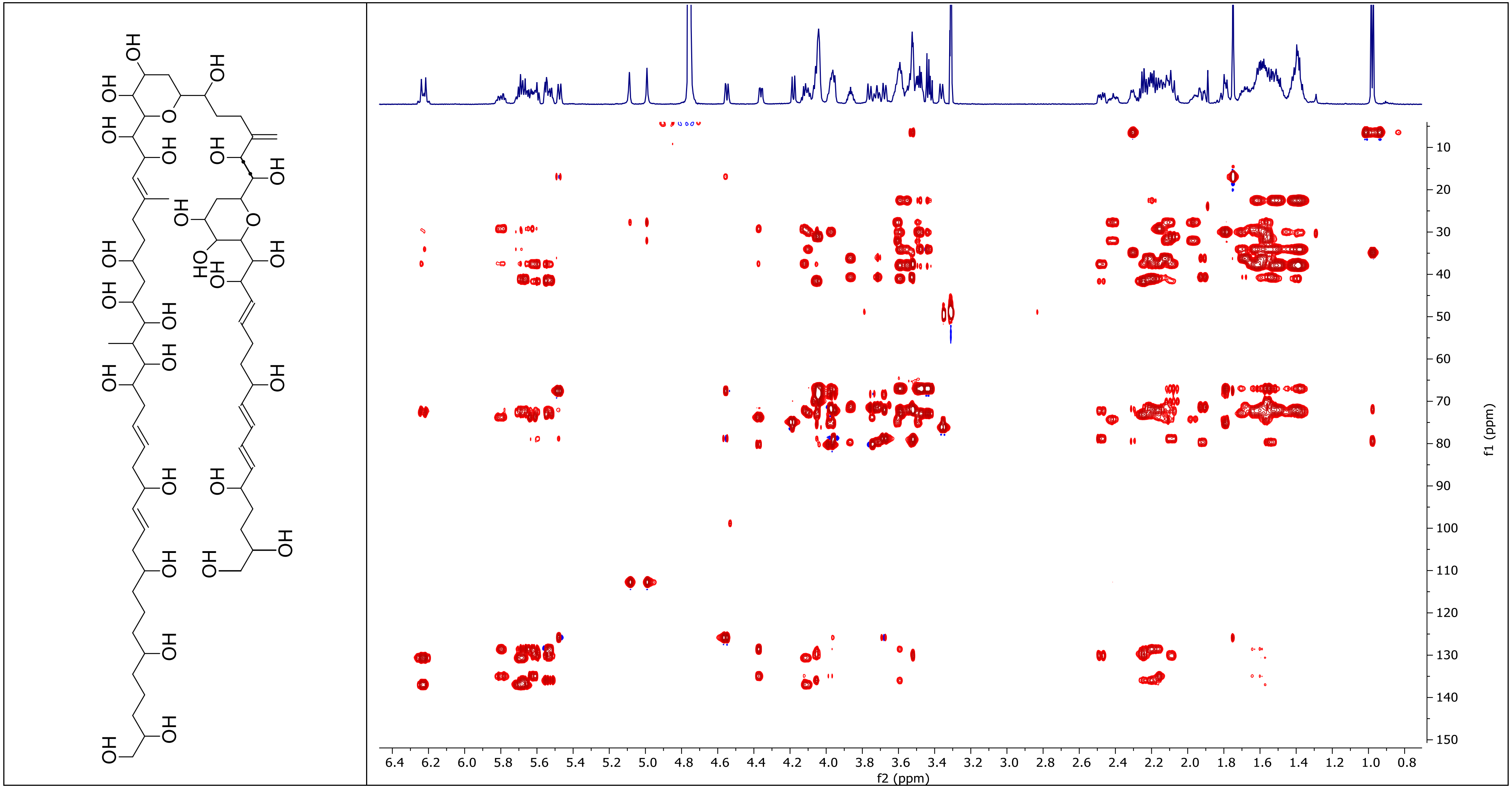

Figure S5. HMBC spectrum (600 MHz, CD_3OD) for amphidinol 24.

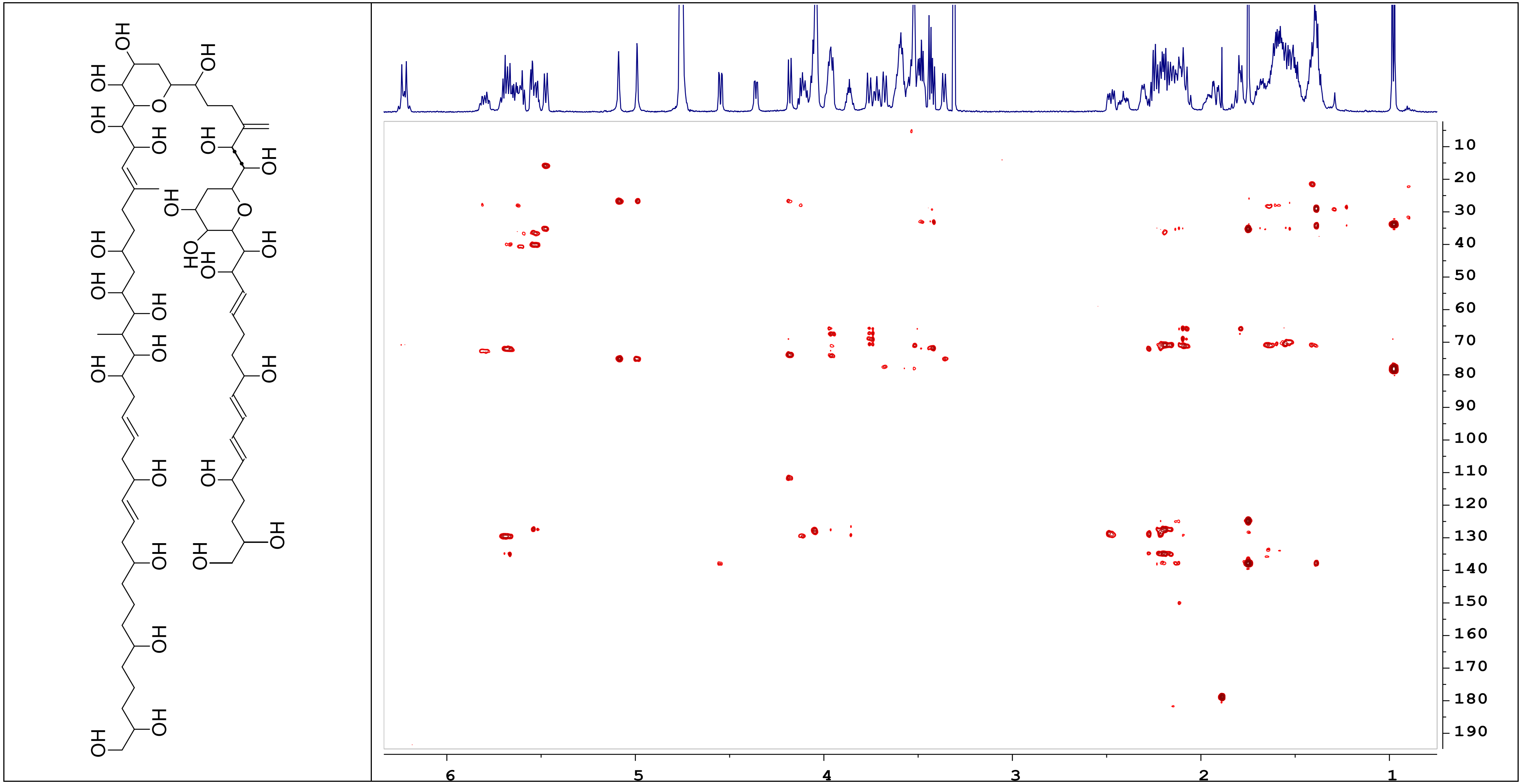

Figure S6. H2BC spectrum(600 MHz, CD_3OD) for amphidinol 24.

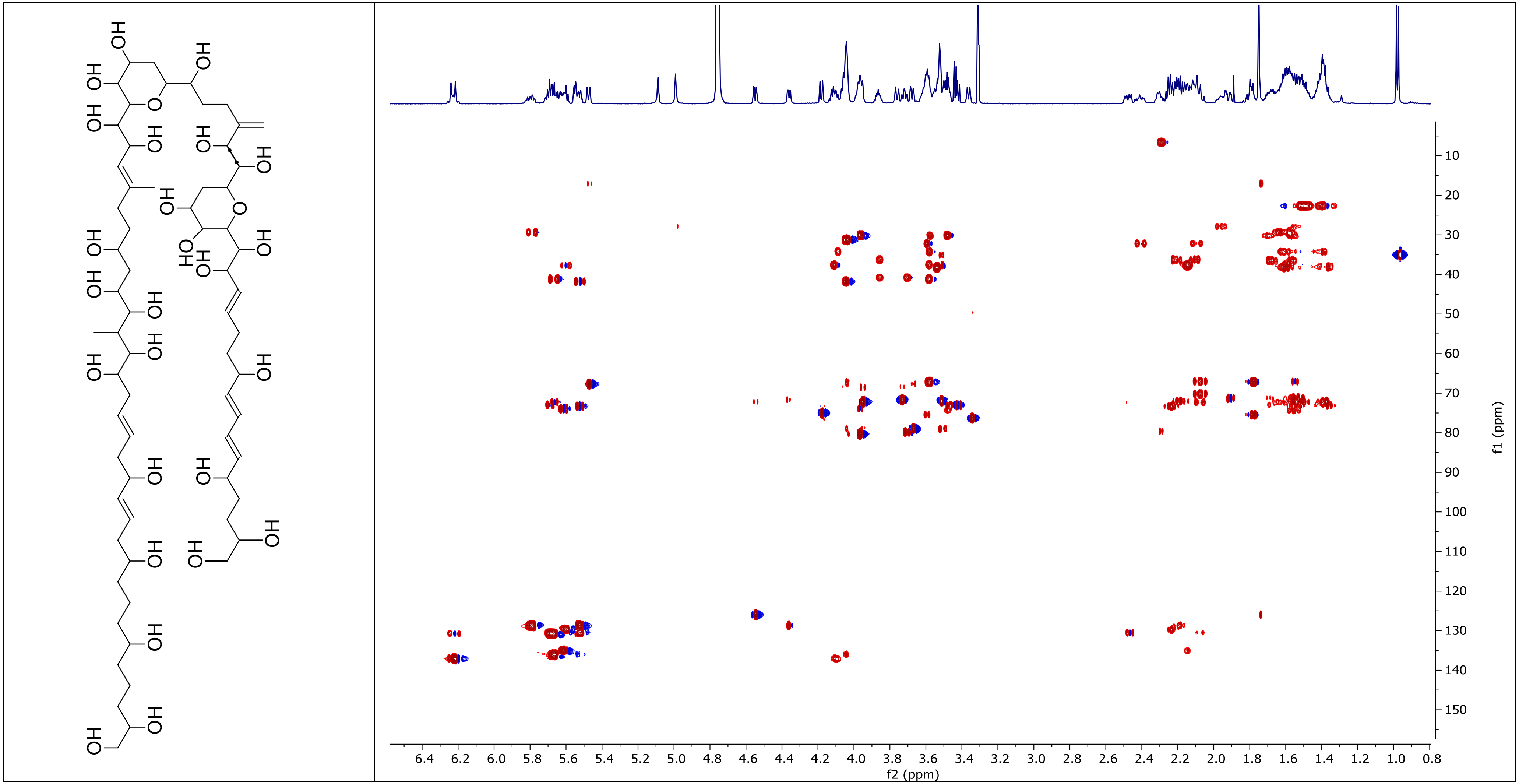

Figure S7. T-ROESY spectrum (600 MHz, CD_3OD) for amphidinol 24.

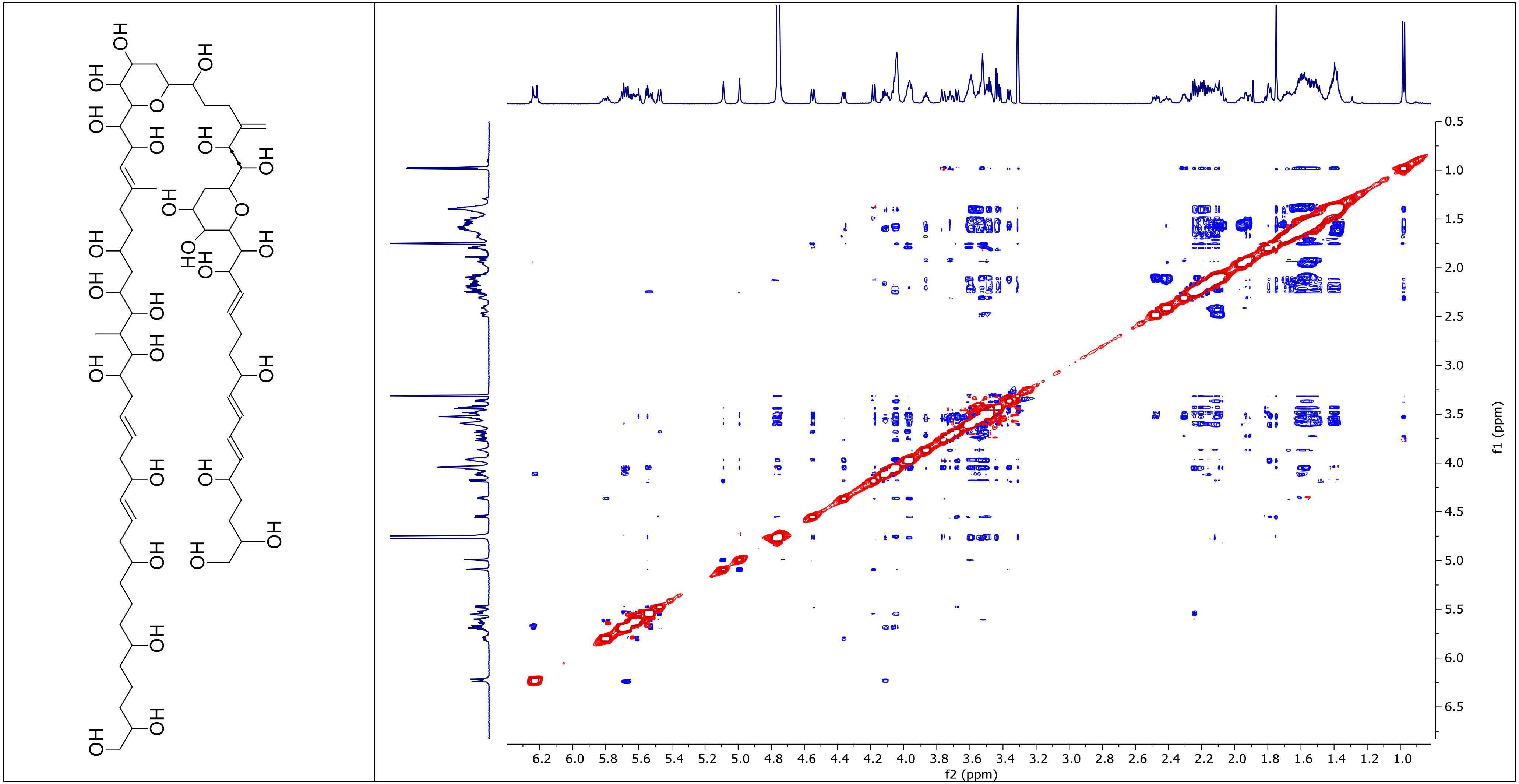

Figure S8. 1D-NOESY spectra (600 MHz, CD_3OD) for amphidinol 24.

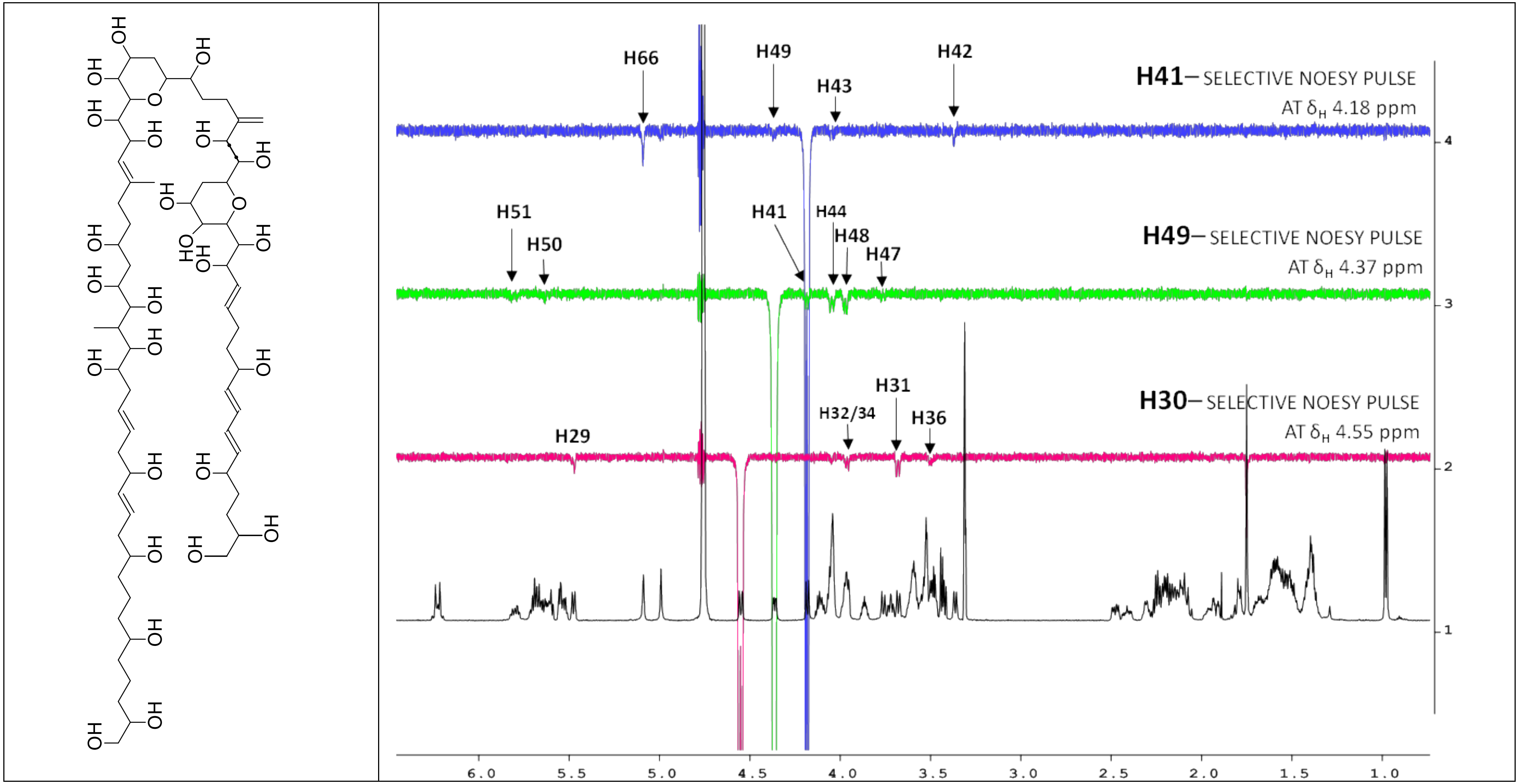

Figure S9. ^{1}H NMR spectrum (600 MHz, CD_3OD-C_5D_5N 2:1) for amphidinol 24.

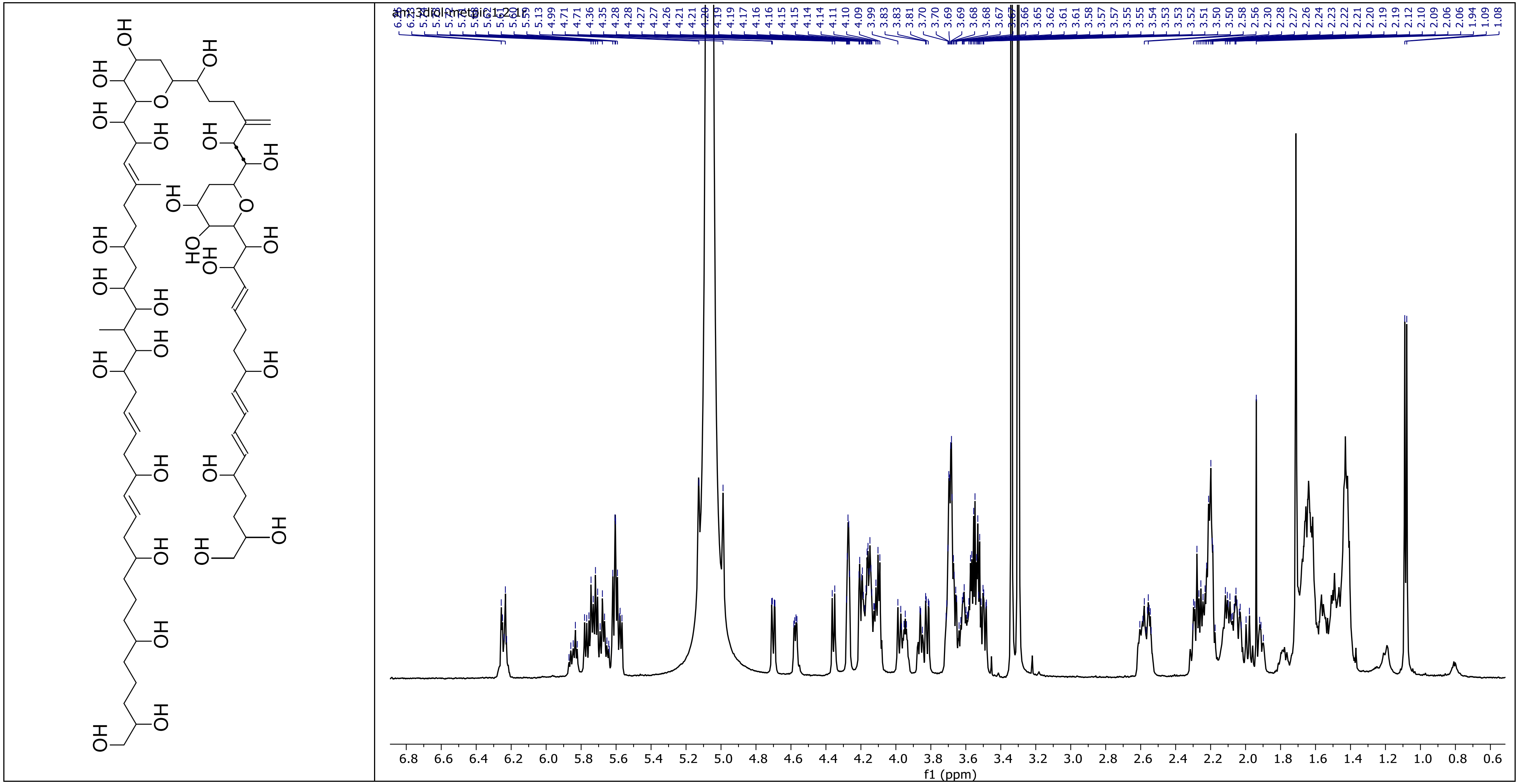

Figure S10. HSQC$_{ed}$ spectrum (600 MHz, CD_3OD-C_5D_5N 2:1) for amphidinol 24.

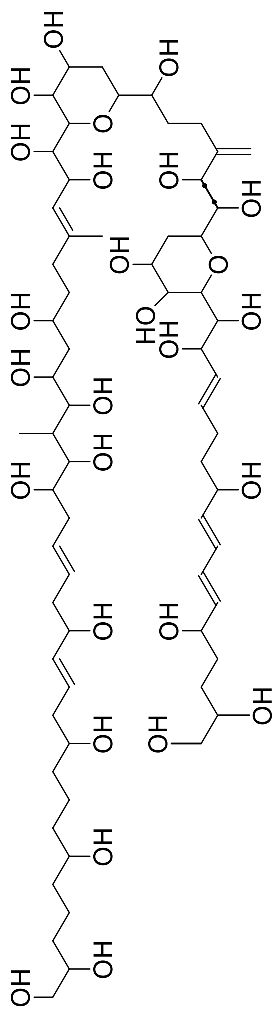

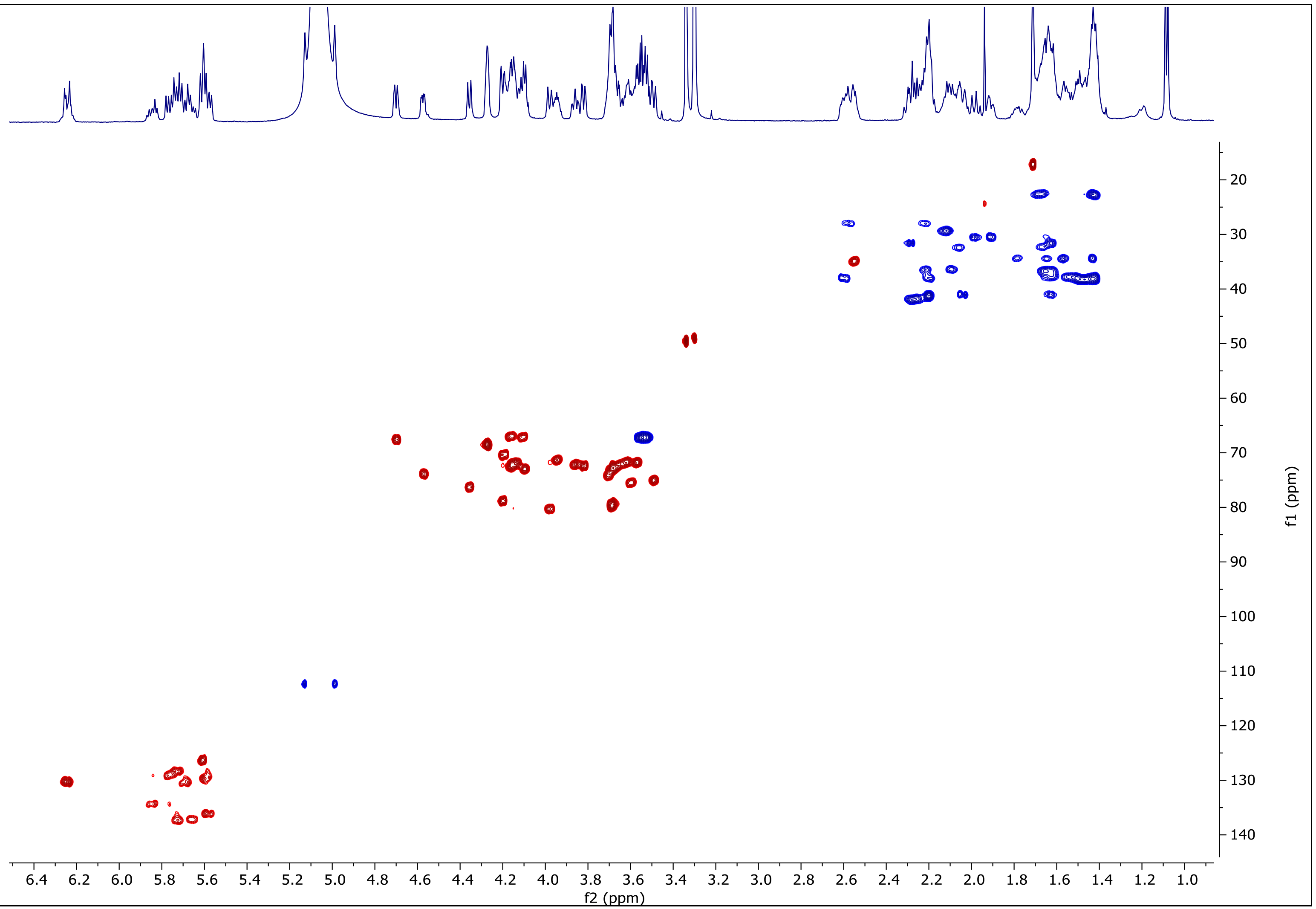

Figure S11. HSQC-TOCSY spectrum (600 MHz, CD_3OD-C_5D_5N 2:1) for amphidinol 24.

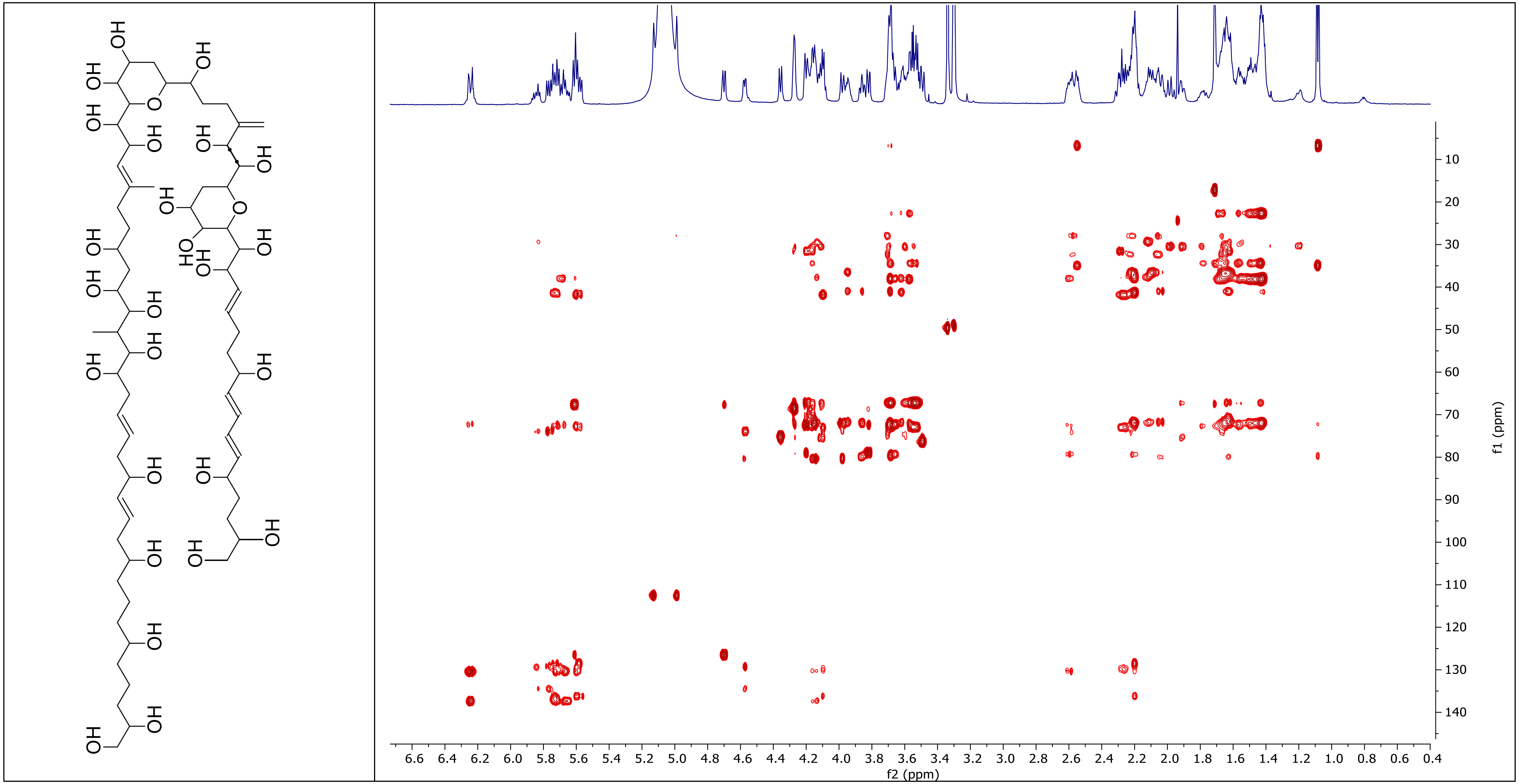

Figure S12. HMBC spectrum (600 MHz, CD_3OD-C_5D_5N 2:1) for amphidinol 24.

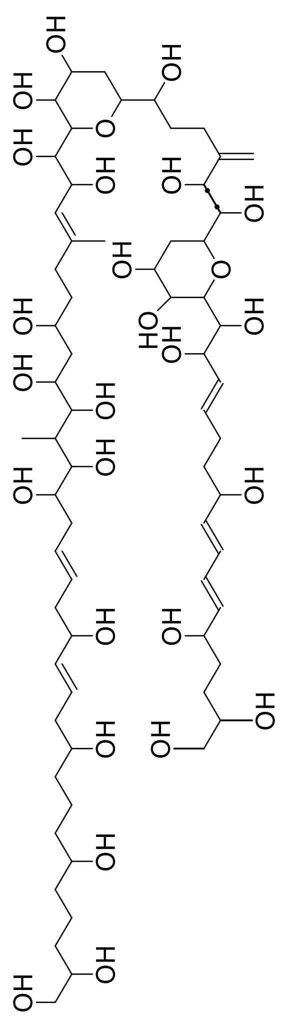

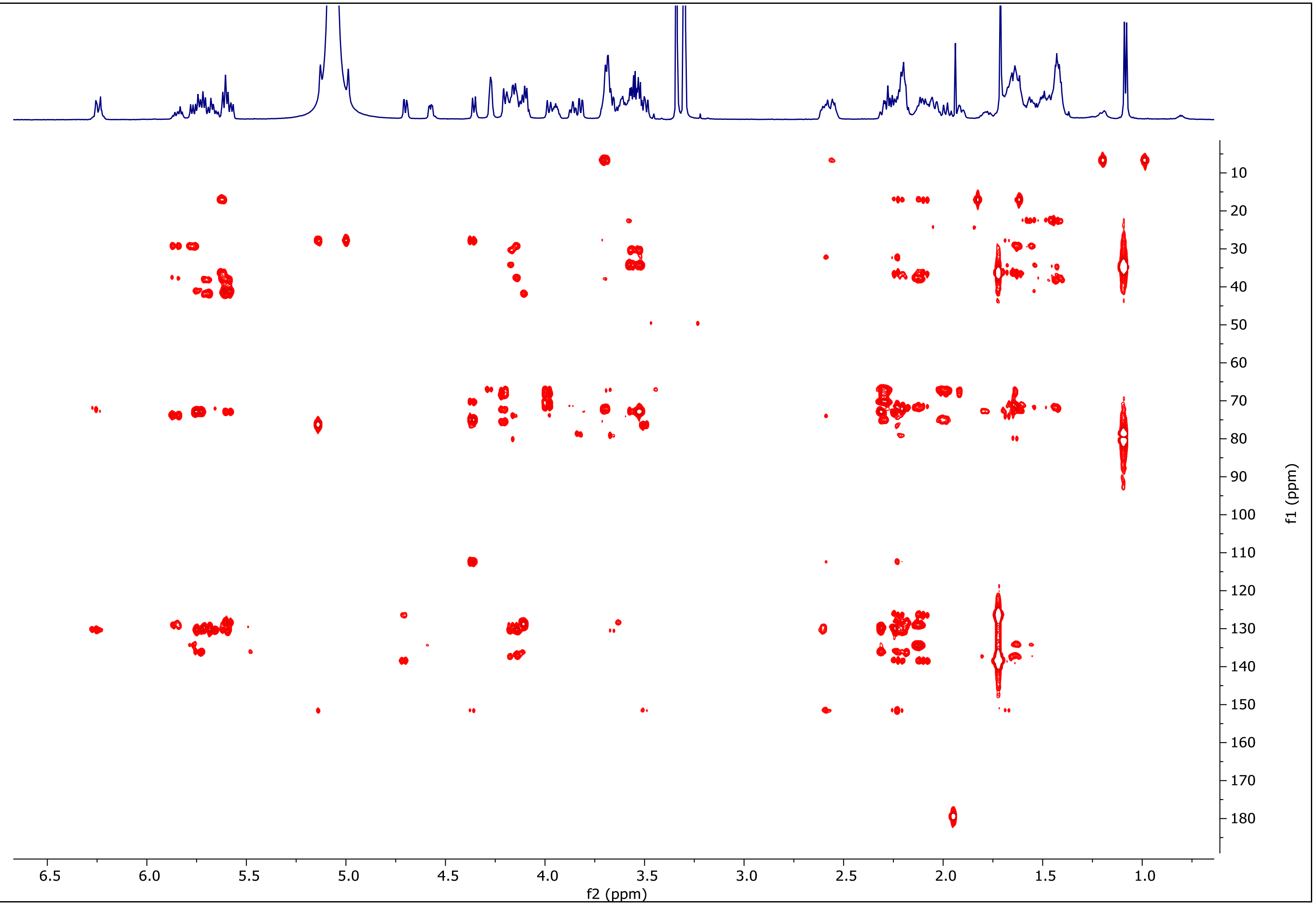

Figure S13. H2BC spectrum (600 MHz, CD_3OD-C_5D_5N 2:1) for amphidinol 24.

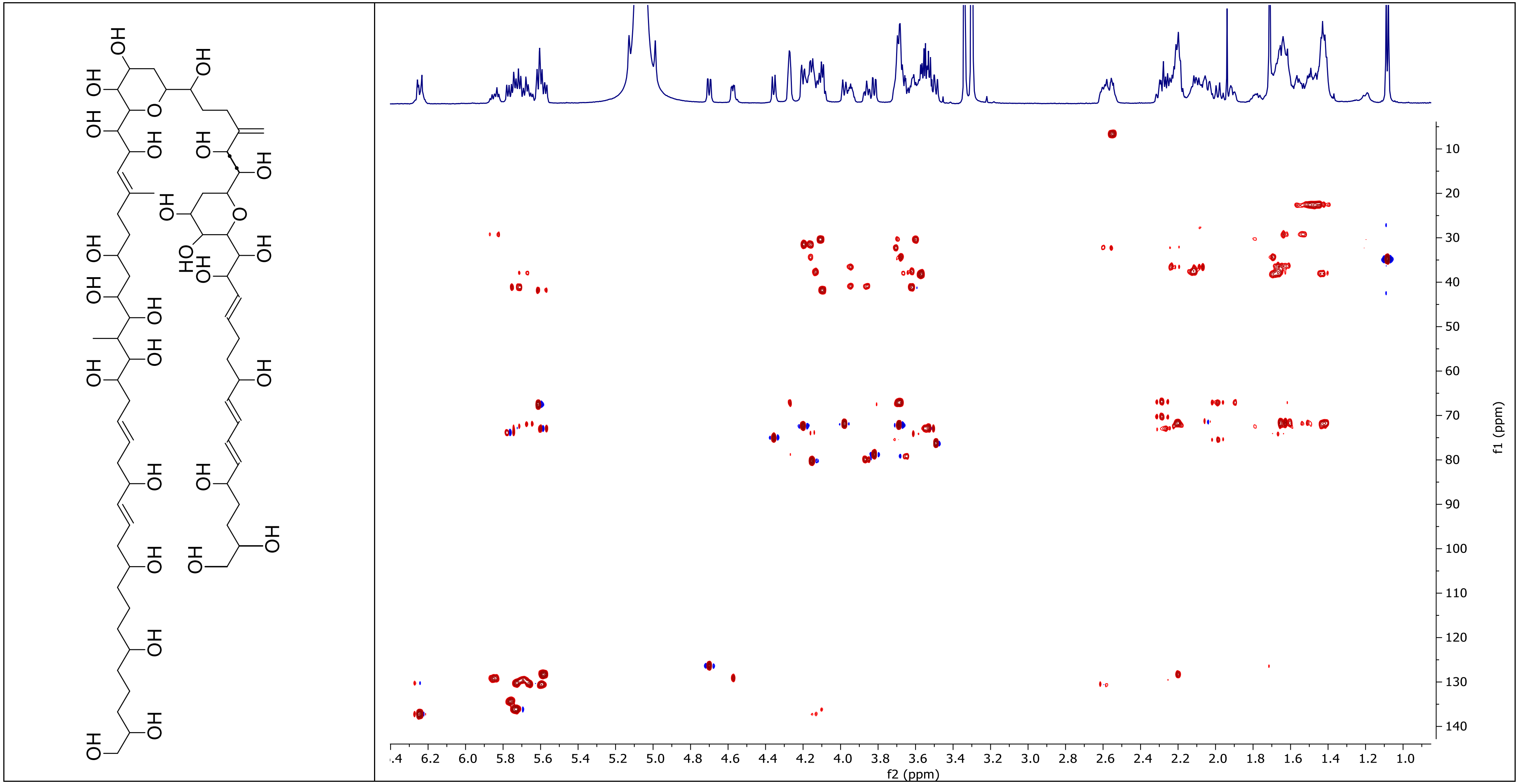

Figure S14. T-ROESY spectrum (600 MHz, CD_3OD-C_5D_5N 2:1) for amphidinol 24.

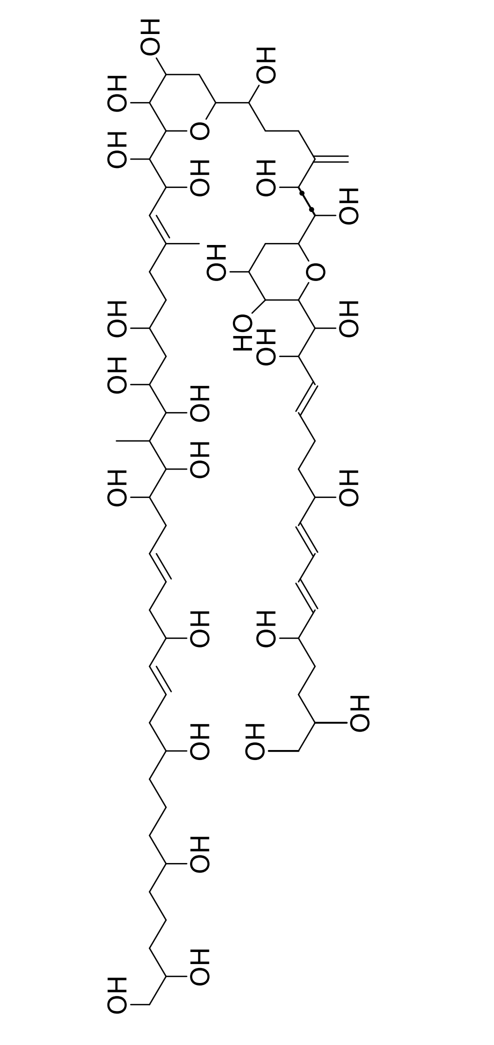

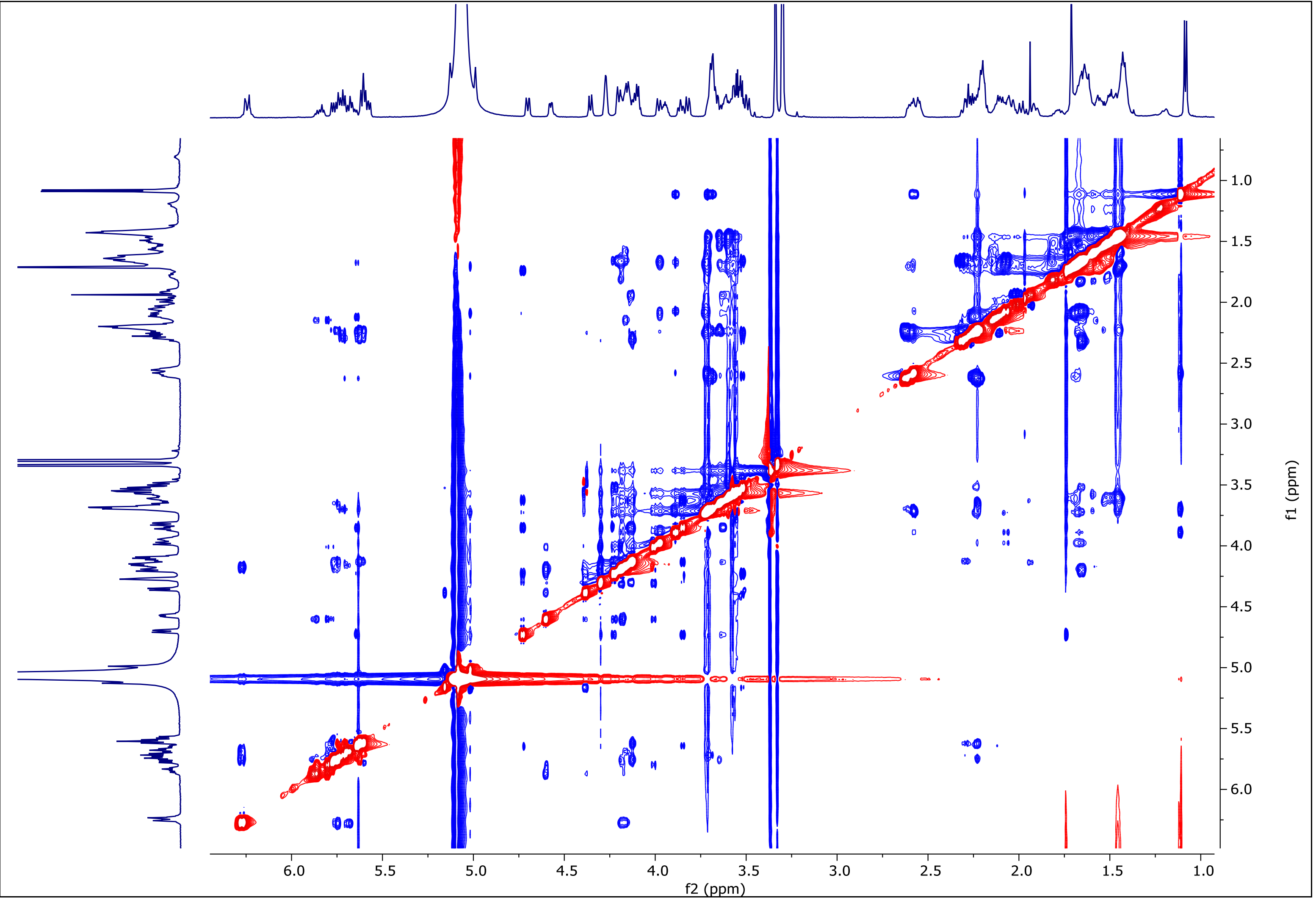

Figure S15. 1D-NOESY spectrum (600 MHz, CD_3OD-C_5D_5N 2:1) for amphidinol 24.

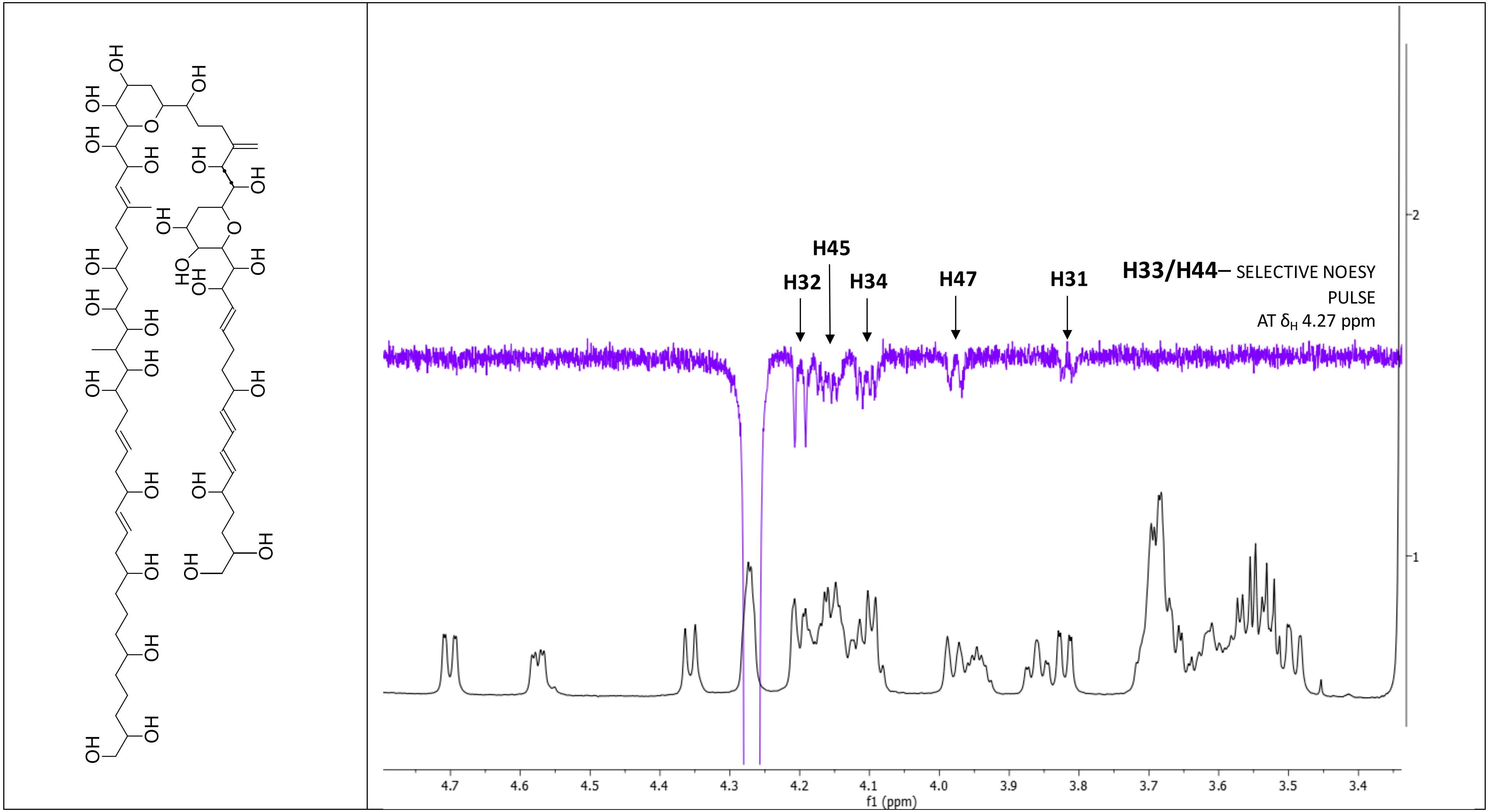

Table S2. ^{1}H and ^{13}C NMR data comparison for carbons C-30 → C-51 in CD_3OD-C_5D_5N 2:1 for amphidinol 24 *versus* related synthetic fragments 4a and 4b reported by Wakamiya et al [22].

#	δC, type CD_3OD-C_5D_5N 2:1	4a	am 24 / 4a	4b	am 24 / 4b
30	67.6, CH	71.1	-3,5	70.9	-3,3
31	72.4, CH	68.3	4,1	67.8	4,6
32	78.9, CH	78.3	0,6	78.9	0
33	68.6, CH	69.1	-0,5	68.9	-0,3
34	67.2, CH	67.3	-0,1	67.3	-0,1
35	30.5, CH_2	31.7	-1,2	30.7	-0,2
36	75.5, CH	74.8	0,7	75	0,5
37	74.1, CH	74.2	-0,1	74.1	0
38	32.4, CH_2	32.2	0,2	32.2	0,2
39	28.0, CH_2	27.8	0,2	27.7	0,3
40	151.5, C	152.0	-0,5	151.9	-0,4
41	76.3, CH	76.5	-0,2	76.5	-0,2
42	75.1, CH	75.4	-0,3	75.3	-0,2
43	70.4, CH	70.6	-0,2	70.6	-0,2
44	31.6, CH_2	31.9	-0,3	31.8	-0,2
45	67.0, CH	67.3	-0,3	67.3	-0,3
46	68.5, CH	68.7	-0,2	68.7	-0,2
47	80.3, CH	80.6	-0,3	80.6	-0,3
48	72.0, CH	72.2	-0,2	72.1	-0,1
49	73.9, CH	74.1	-0,2	74.1	-0,2
50	129.2, CH	129.8	-0,6	129.7	-0,5
51	134.4, CH	134.1	0,3	134.2	0,2

#	δH CD_3OD-C_5D_5N 2:1	4a	am 24 / 4a	4b	am 24 / 4b
30	4.7	4.08	0,62	4.07	0,63
31	3.82	4.14	-0,32	4.15	-0,33
32	4.2	4.26	-0,06	4.25	-0,05
33	4.27	4.33	-0,06	4.36	-0,09
34	4.11	4.11	0	4.11	0
35	1.91	1.69	0,22	1.90	0,01
	1.99	2.08	-0,09	2.02	-0,03
36	3.6	3.62	-0,02	3.60	0
37	3.7	3.65	0,05	3.73	-0,03
38	1.67	1.73	-0,06	1.72	-0,05
	2.06	1.91	0,15	1.96	0,1
39	2.22	2.24	-0,02	2.24	-0,02
	2.58	2.54	0,04	2.56	0,02
41	4.36	4.39	-0,03	4.38	-0,02
42	3.5	3.51	-0,01	3.50	0
43	4.19	4.19	0	4.21	-0,02
44	1.63	1.64	-0,01	1.62	0,01
	2.29	2.34	-0,05	2.33	-0,04
45	4.16	4.18	-0,02	4.17	-0,01
46	4.28	4.34	-0,06	4.33	-0,05
47	3.97	4.02	-0,05	4.00	-0,03
48	4.15	4.20	-0,05	4.19	-0,04
49	4.57	4.63	-0,06	4.61	-0,04
50	5.76	5.78	-0,02	5.76	0
51	5.85	5.81	0,04	5.81	0,04

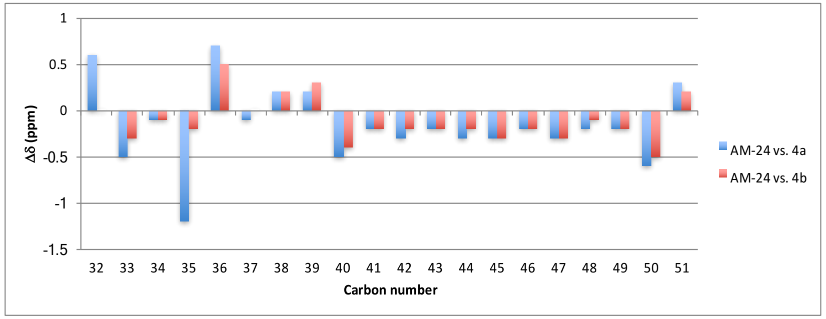

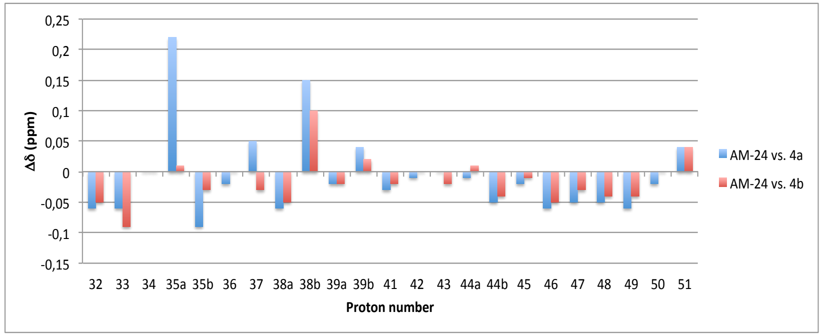

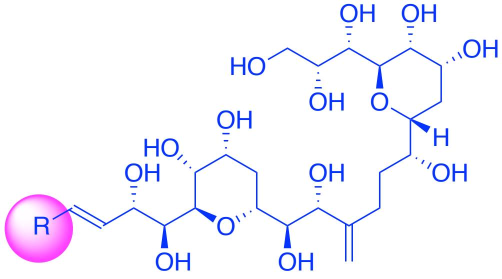

4a

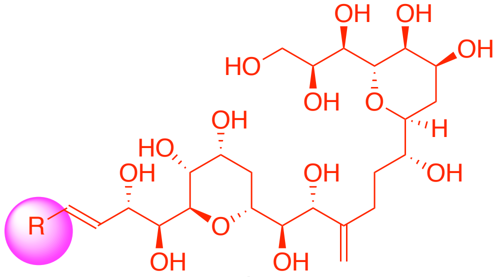

4b

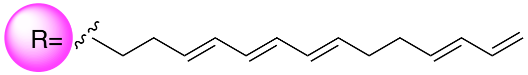

Figure S16. HRESIMS spectrum for amphidinol 24.

Elemental Composition Report **Page 1**

Multiple Mass Analysis: 3 mass(es) processed
Tolerance = 5.0 PPM / DBE: min = -1.5, max = 100.0
Element prediction: Off
Number of isotope peaks used for i-FIT = 3

Monoisotopic Mass, Even Electron Ions
303 formula(e) evaluated with 5 results within limits (up to 50 best isotopic matches for each mass)
Elements Used:
C: 0-70 H: 0-120 O: 0-30 Na: 0-1
David
(ESI 18-69) Adrian M (AcO 3 Sigma 1 B (F+G) 7) 65 (2.843) 1: TOF MS ES+
1.12e+003

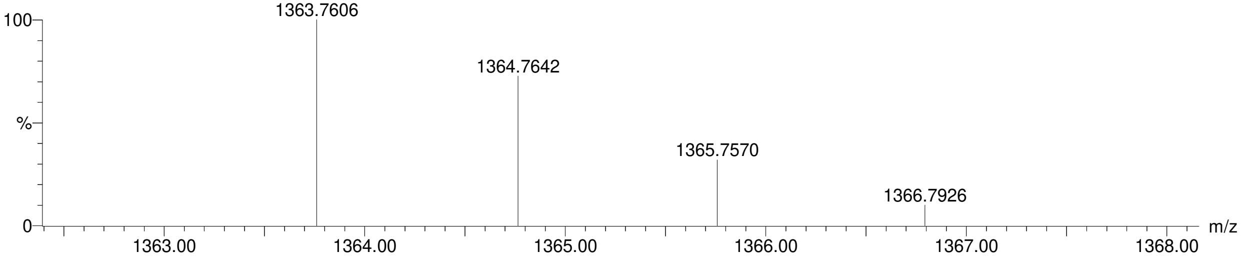

Minimum:	15.00				-1.5			
Maximum:	100.00		5.0	5.0	100.0			
Mass	RA	Calc. Mass	mDa	PPM	DBE	i-FIT	i-FIT (Norm)	Formula
1363.7606	100.00	1363.7602	0.4	0.3	8.5	11.6	0.2	C66 H116 O27 Na
		1363.7626	-2.0	-1.5	11.5	13.0	1.6	C68 H115 O27
1364.7642	72.64	---						
1365.7570	31.81	1365.7630	-6.0	-4.4	6.5	25.5	0.9	C64 H117 O30
		1365.7606	-3.6	-2.6	3.5	25.5	1.0	C62 H118 O30 Na
		1365.7547	2.3	1.7	12.5	26.0	1.5	C69 H114 O25 Na

Figure S17. Main MS/MS fragments observed for amphidinol 24.

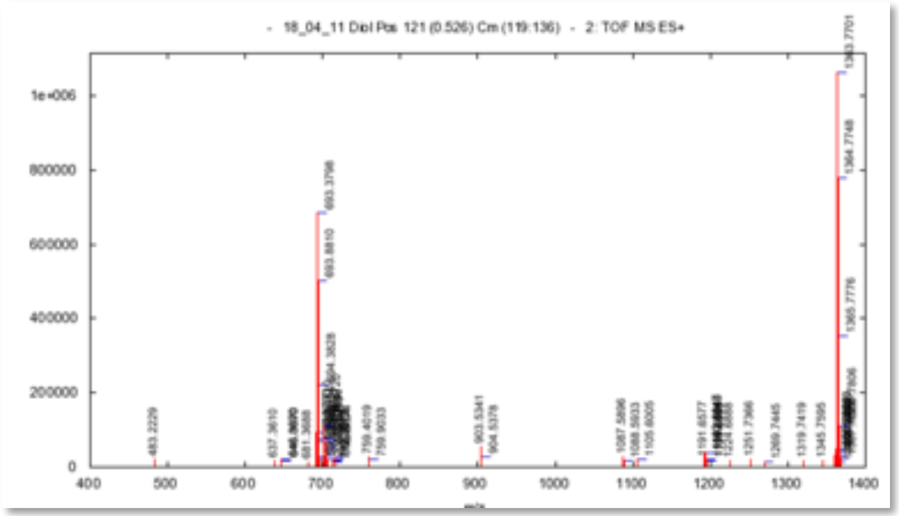

ID	*m/z*	Formula	Calc.	Deviation	Losses	Add.	Score	Bonds
1	1251.7366	$C_{63}H_{111}O_{24}$	1251.7465	- 9.9 mDa	- $C_3H_6O_3$	+ 3H	1.5	2
2	1251.7366	$C_{63}H_{111}O_{24}$	1251.7465	- 9.9 mDa	- $C_3H_6O_3$	+ 3H	1.5	2
3	1224.6888	$C_{60}H_{104}O_{24}$	1224.6867	+ 2.1 mDa	- $C_6H_{13}O_2$	+ 2H	2.0	2
4	1193.6669	$C_{59}H_{101}O_{24}$	1193.6683	- 1.4 mDa	- $C_7H_{16}O_3$	+ 0H	5.0	2
5	1193.6669	$C_{59}H_{101}O_{24}$	1193.6683	- 1.4 mDa	- $C_7H_{16}O_3$	+ 0H	5.0	2
6	759.4019	$C_{34}H_{63}O_{18}$	759.4019	+ 0.5 mDa	- $C_{32}H_{54}O_9$	+ 3H	2.0	2
7	705.3731	$C_{34}H_{57}O_{15}$	705.3731	+ 3.4 mDa	- $C_{32}H_{60}O_{12}$	+ 1H	1.5	2
8	702.3754	$C_{35}H_{58}O_{18}$	702.3754	- 7.3 mDa	- $C_{32}H_{59}O_{13}$	- 2H	2.0	2
9	701.3720	$C_{35}H_{57}O_{14}$	701.3720	- 2.8mDa	- $C_{31}H_{60}O_{13}$	+ 1H	2.0	2
10	695.3849	$C_{33}H_{58}O_{15}$	695.3849	- 0.5 mDa	- $C_{33}H_{58}O_{12}$	+ 3H	2.0	2
11	694.3828	$C_{35}H_{58}O_{15}$	694.3828	+ 5.2 mDa	- $C_{33}H_{59}O_{12}$	+ 2H	2.0	2
12	681.3688	$C_{32}H_{57}O_{15}$	681.3697	- 0.9 mDa	- $C_{34}H_{60}O_{12}$	+ 3H	2.0	2

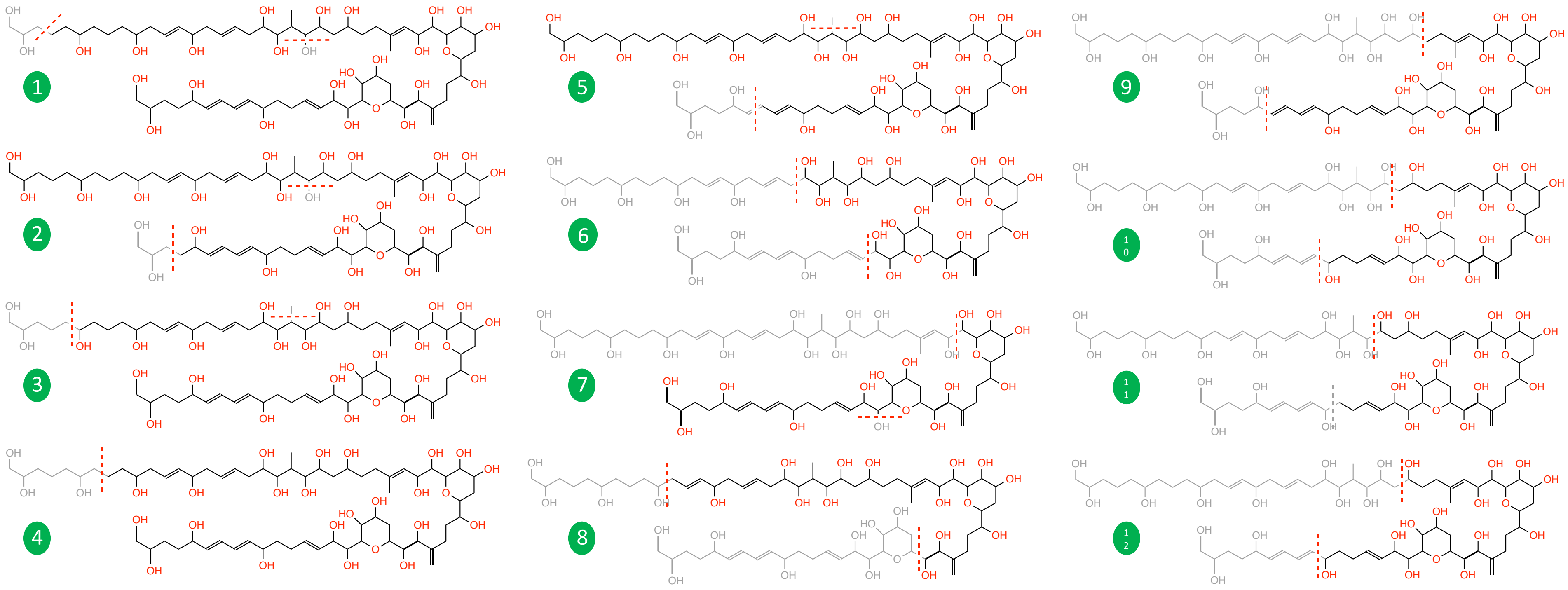

Figure S18.^{1}H NMR spectrum (600 MHz, CD_3OD) for amphidinol 25.

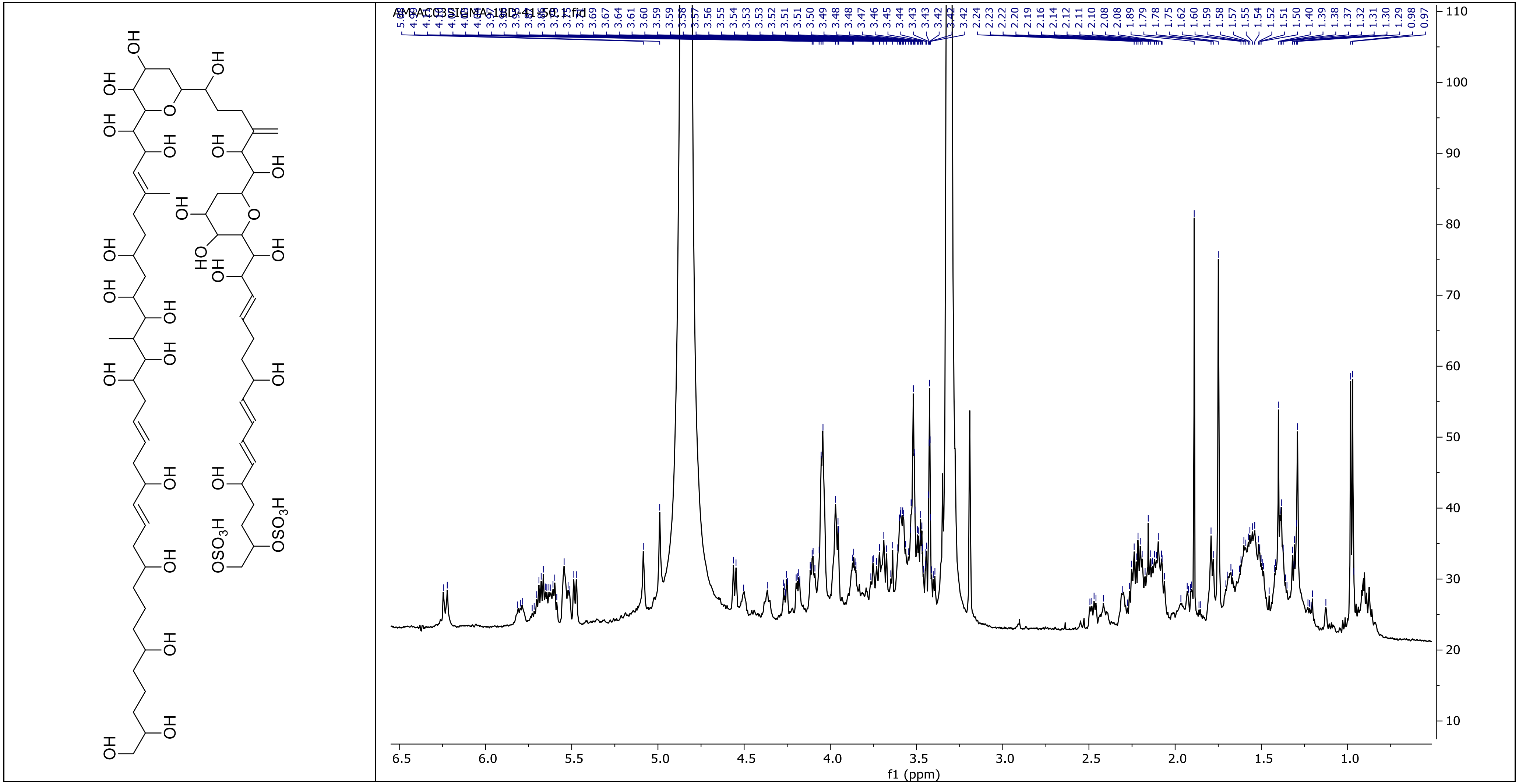

Figure S19. COSY spectrum (600 MHz, CD_3OD) for amphidinol 25.

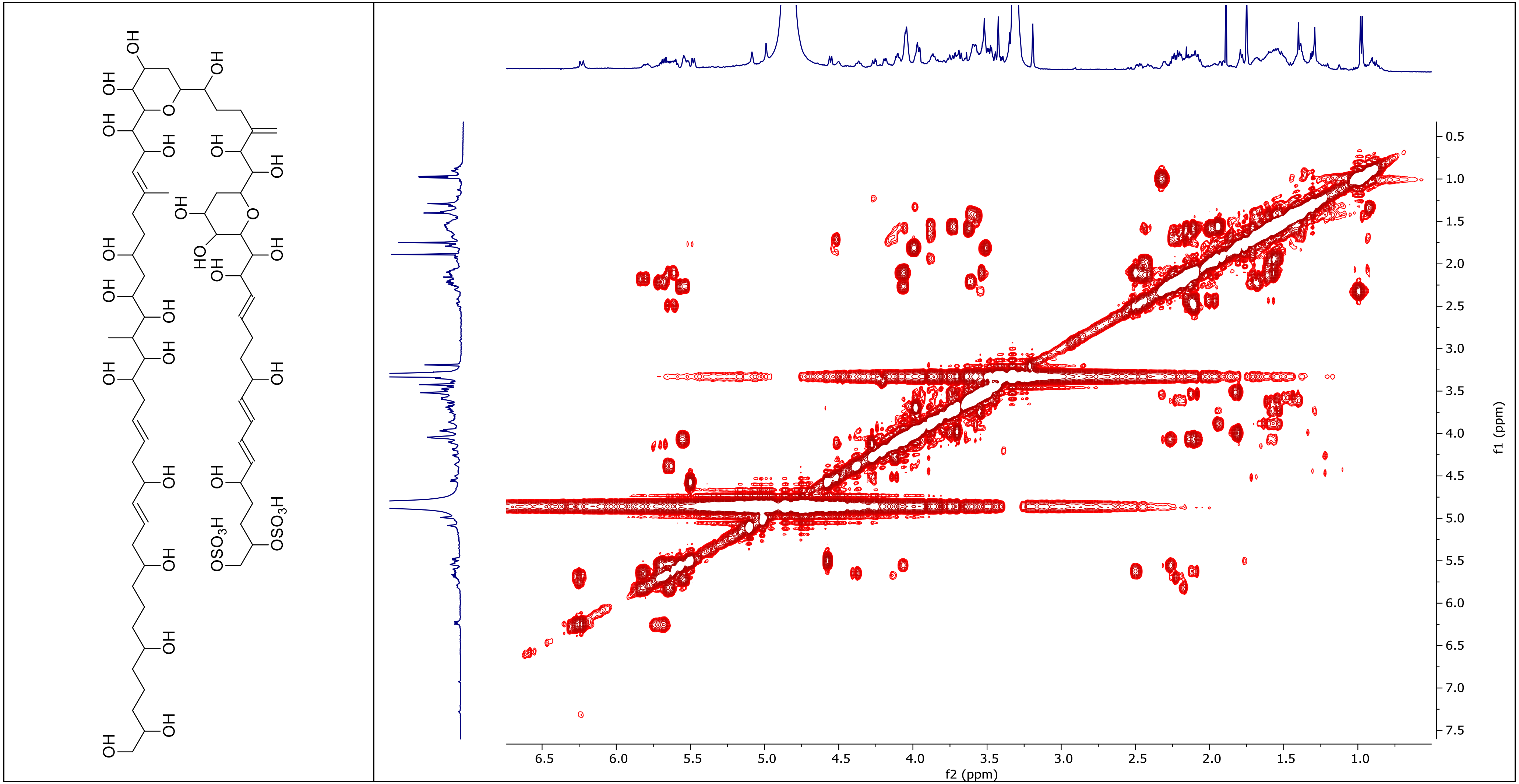

Figure S20. HSQCed spectrum (600 MHz, CD_3OD) for amphidinol 25.

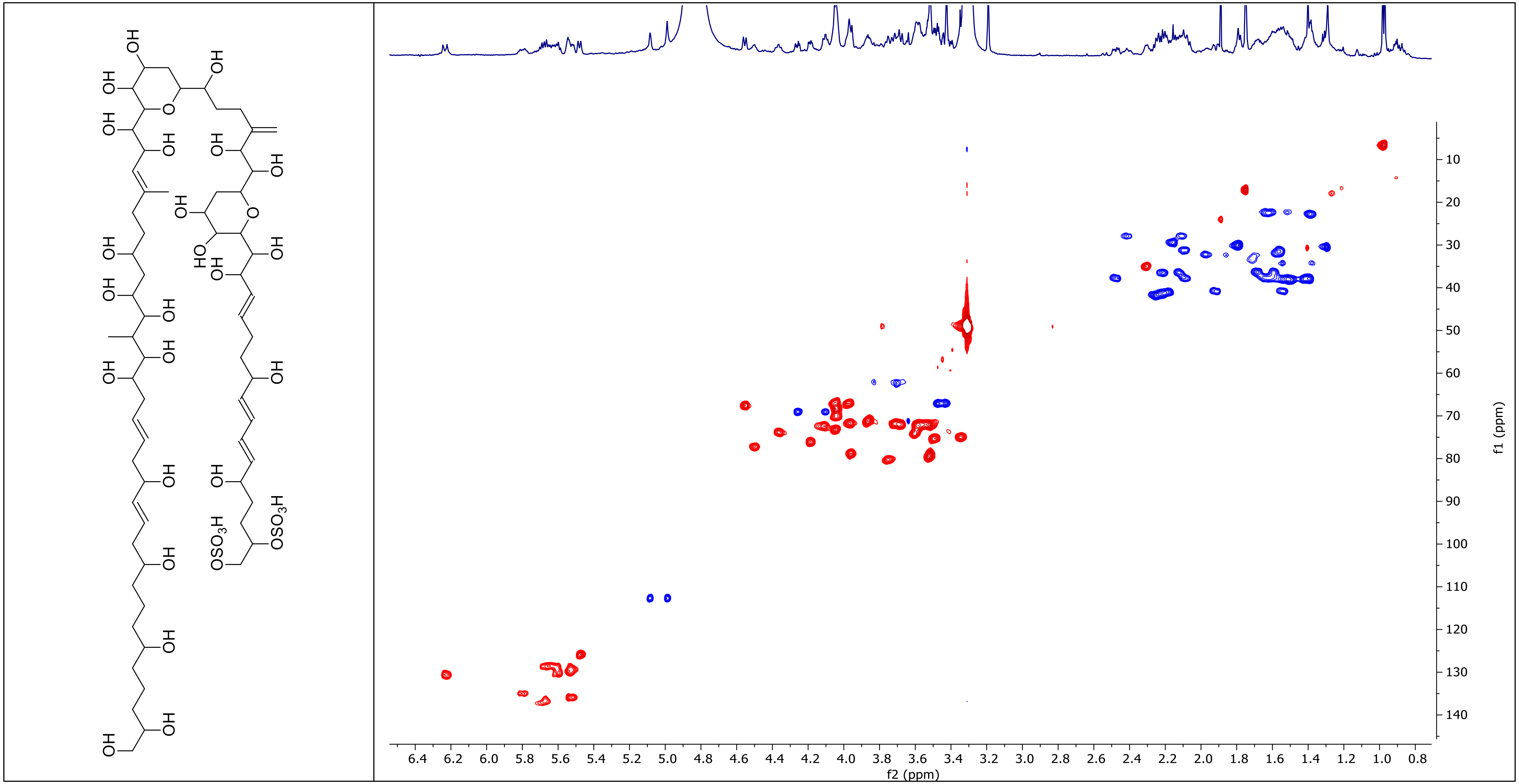

Figure S21. HSQC-TOCSY spectrum (600 MHz, CD_3OD) for amphidinol 25.

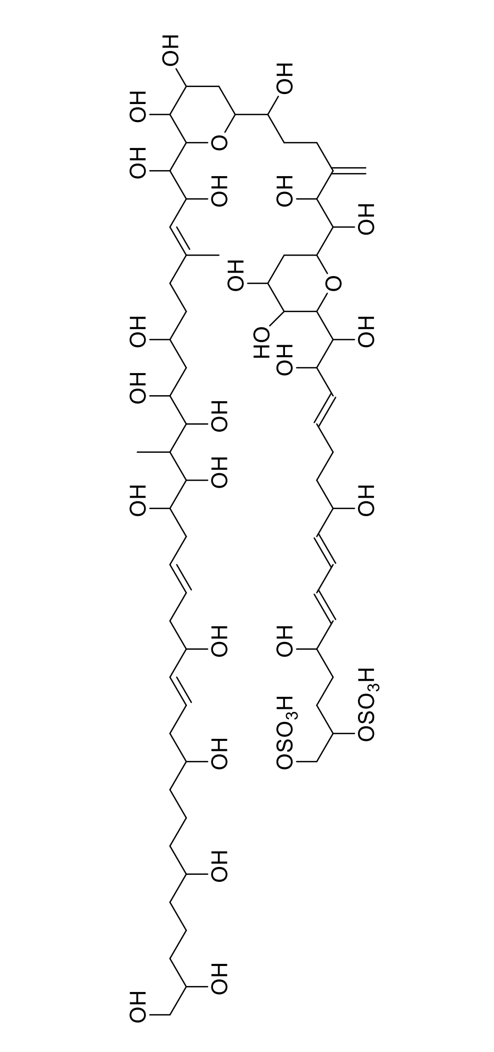

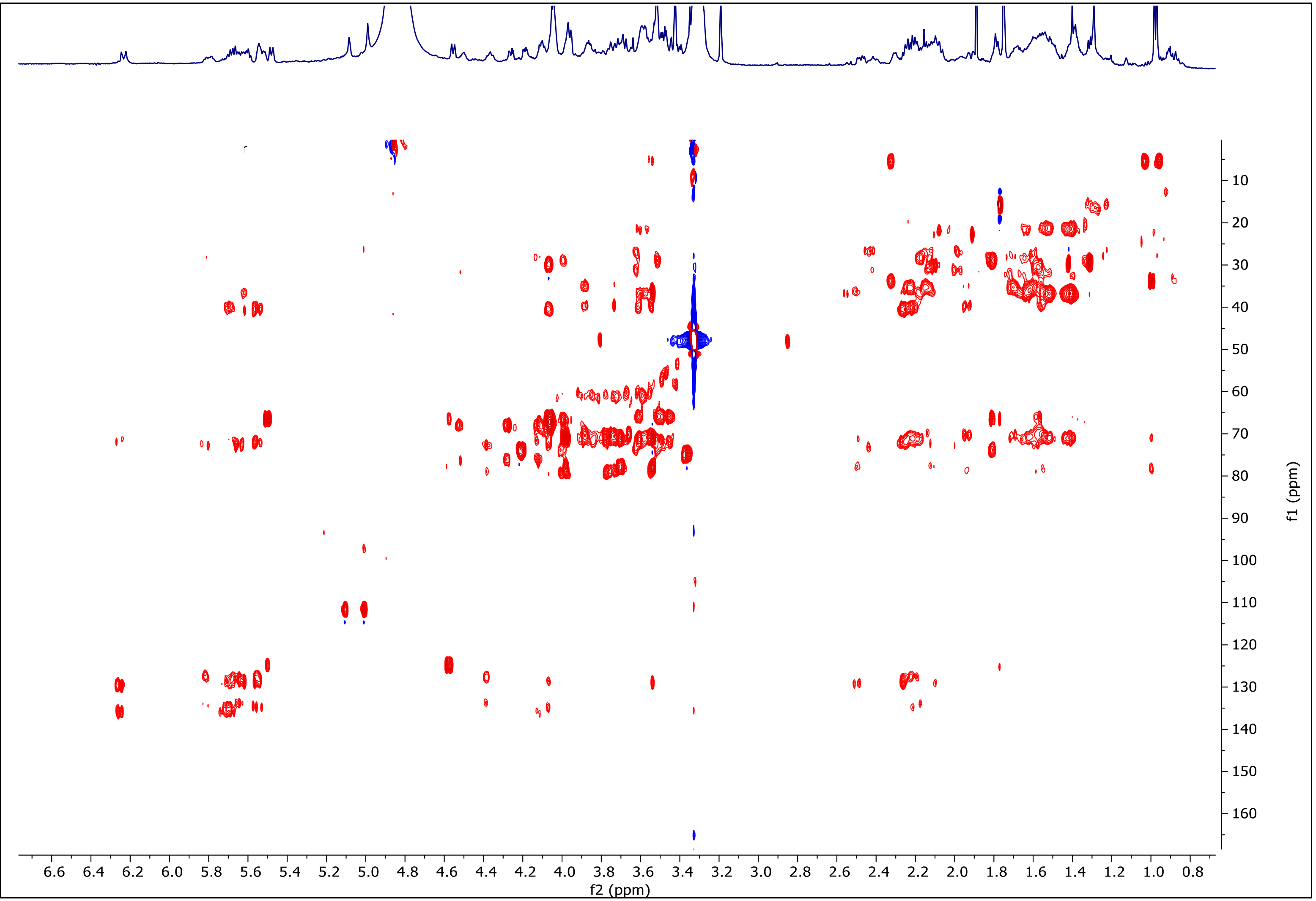

Figure S22. HMBC spectrum (600 MHz, CD_3OD) for amphidinol 25.

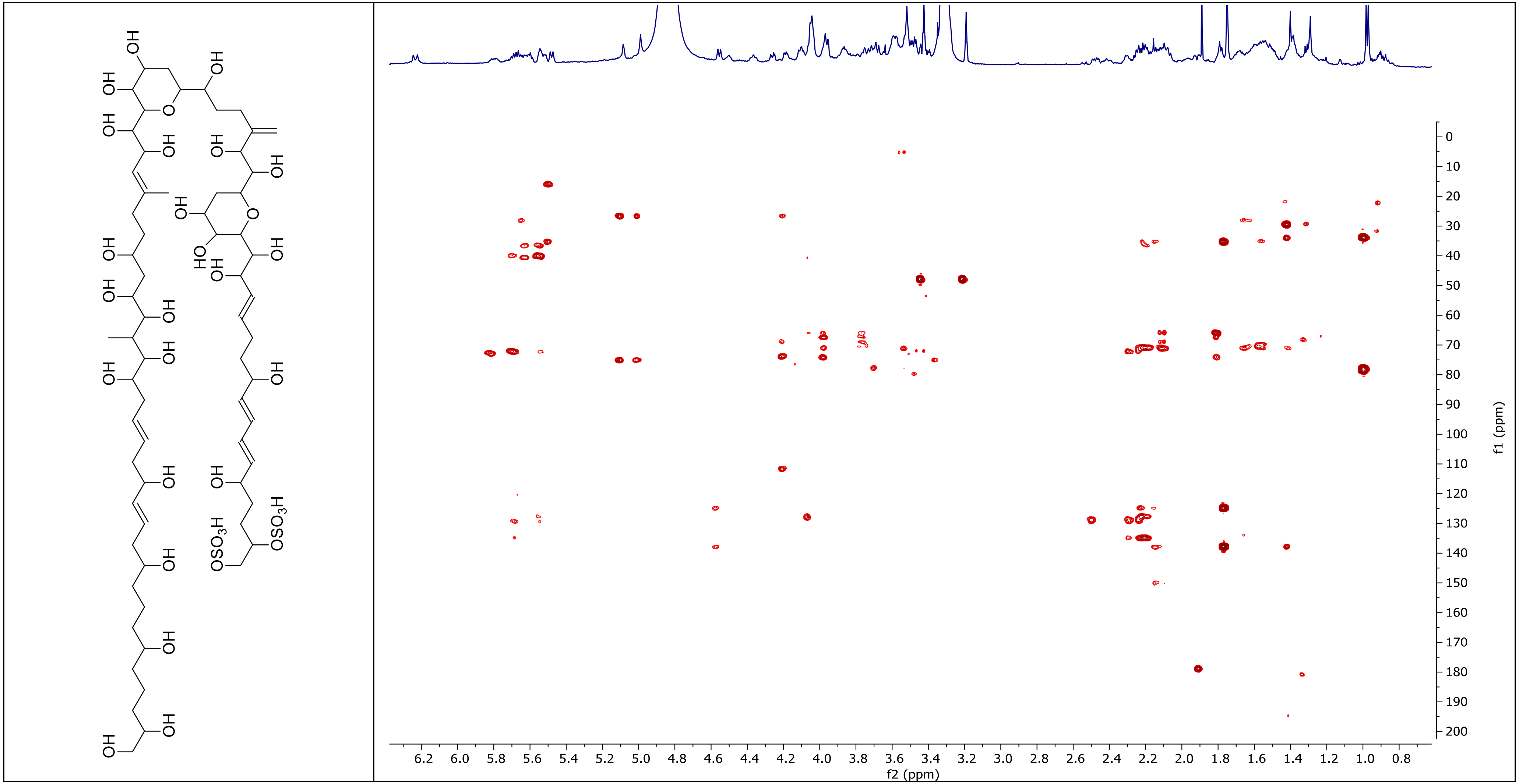

Figure S23. HRESIMS spectrum for amphidinol 25

Elemental Composition Report **Page 1**

Single Mass Analysis
Tolerance = 10.0 PPM / DBE: min = -50.0, max = 75.0
Element prediction: Off
Number of isotope peaks used for i-FIT = 3

Monoisotopic Mass, Odd and Even Electron Ions
1111 formula(e) evaluated with 31 results within limits (all results (up to 1000) for each mass)
Elements Used:
C: 5-80 H: 0-150 O: 0-40 Na: 0-1 S: 0-2
18_04_11 Sulfatado NEG 161 (0.694) Cm (159:177)
1: TOF MS ES-

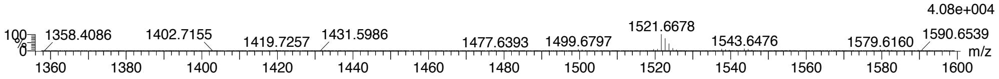

Minimum:				-50.0				
Maximum:		40.0	10.0	75.0				

Mass	Calc. Mass	mDa	PPM	DBE	i-FIT	Norm	Conf(%)	Formula
1521.6678	1521.6819	-14.1	-9.3	27.5	409.5	9.702	0.01	C80 H106 O27 Na
	1521.6642	3.6	2.4	27.5	407.7	7.829	0.04	C80 H106 O25 Na S
	1521.6699	-2.1	-1.4	25.5	408.6	8.750	0.02	C79 H109 O25 S2
	1521.6691	-1.3	-0.9	26.5	406.2	6.395	0.17	C78 H105 O30
	1521.6675	0.3	0.2	22.5	408.0	8.184	0.03	C77 H110 O25 Na S2
	1521.6667	1.1	0.7	23.5	406.0	6.141	0.22	C76 H106 O30 Na
	1521.6724	-4.6	-3.0	21.5	406.2	6.364	0.17	C75 H109 O30 S
	1521.6547	13.1	8.6	21.5	409.7	9.834	0.01	C75 H109 O28 S2
	1521.6538	14.0	9.2	22.5	408.7	8.831	0.01	C74 H105 O33
	1521.6700	-2.2	-1.4	18.5	405.2	5.362	0.47	C73 H110 O30 Na S
	1521.6758	-8.0	-5.3	16.5	407.1	7.260	0.07	C72 H113 O30 S2
	1521.6572	10.6	7.0	17.5	406.2	6.323	0.18	C71 H109 O33 S
	1521.6749	-7.1	-4.7	17.5	406.5	6.659	0.13	C71 H109 O35
	1521.6734	-5.6	-3.7	13.5	405.5	5.700	0.33	C70 H114 O30 Na S2
	1521.6725	-4.7	-3.1	14.5	406.3	6.487	0.15	C69 H110 O35 Na
	1521.6548	13.0	8.5	14.5	406.3	6.431	0.16	C69 H110 O33 Na S
	1521.6783	-10.5	-6.9	12.5	404.9	5.111	0.60	C68 H113 O35 S
	1521.6606	7.2	4.7	12.5	404.7	4.849	0.78	C68 H113 O33 S2
	1521.6597	8.1	5.3	13.5	407.4	7.617	0.05	C67 H109 O38
	1521.6759	-8.1	-5.3	9.5	405.2	5.321	0.49	C66 H114 O35 Na S
	1521.6581	9.7	6.4	9.5	401.6	1.726	17.79	C66 H114 O33 Na S2
	1521.6817	-13.9	-9.1	7.5	400.4	0.536	58.49	C65 H117 O35 S2
	1521.6573	10.5	6.9	10.5	408.7	8.904	0.01	C65 H110 O38 Na
	1521.6808	-13.0	-8.5	8.5	409.3	9.488	0.01	C64 H113 O40
	1521.6631	4.7	3.1	8.5	405.3	5.496	0.41	C64 H113 O38 S
	1521.6793	-11.5	-7.6	4.5	401.6	1.780	16.87	C63 H118 O35 Na S2
	1521.6784	-10.6	-7.0	5.5	409.3	9.471	0.01	C62 H114 O40 Na
	1521.6606	7.2	4.7	5.5	407.0	7.193	0.08	C62 H114 O38 Na S
	1521.6664	1.4	0.9	3.5	403.7	3.880	2.07	C61 H117 O38 S2
	1521.6640	3.8	2.5	0.5	406.2	6.359	0.17	C59 H118 O38 Na S2
	1521.6818	-14.0	-9.2	0.5	409.4	9.595	0.01	C59 H118 O40 Na S

Figure S24. Main MS/MS fragments observed for amphidinol 25.

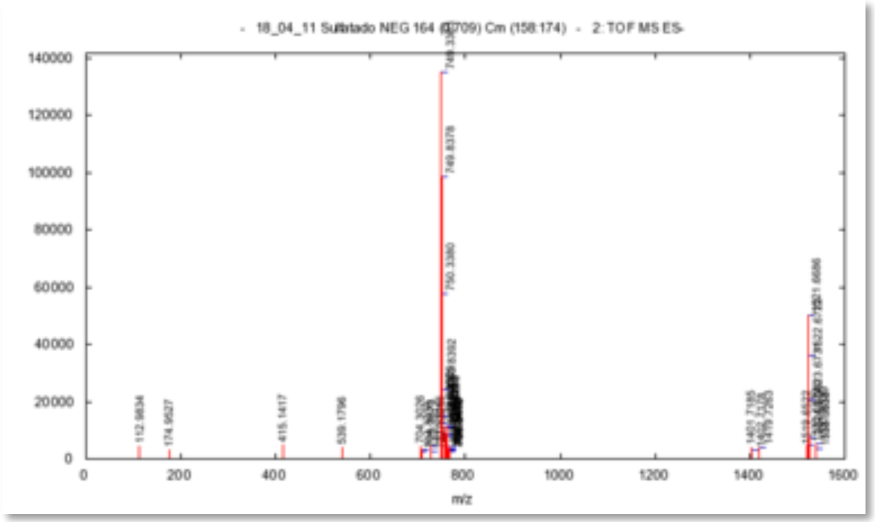

ID	*m/z*	Formula	Calc.	Deviation	Losses	Add.	Score	Bonds
1	1419.7263	$C_{66}H_{115}O_{30}S$	1419.7194	+ 6.9 mDa	- O_3S	+ 2H	1.0	1
2	1402.7178	$C_{66}H_{114}O_{29}S$	1402.7166	+ 1.2 mDa	- HO_4S	+ 1H	0.5	1
3	1402.7178	$C_{66}H_{114}O_{29}S$	1402.7166	+ 1.2 mDa	- HO_4S	+ 1H	0.5	1
4	1401.7185	$C_{66}H_{113}O_{29}S$	1401.7088	+ 9.7 mDa	- H_2O_4S	+ 0H	0.5	1
5	709.3573	$C_{33}H_{57}O_{16}$	709.3647	- 7.4 mDa	- $C_{33}H_{58}O_{17}S_2$	+ 3H	2.0	2
6	709.3573	$C_{33}H_{58}O_{16}$	709.3647	- 7.4 mDa	- $C_{33}H_{58}O_{17}S_2$	+ 1H	2.0	2
7	539.1706	$C_{22}H_{35}O_{13}S$	539.1798	- 0.2 mDa	- $C_{44}H_{80}O_{20}S$	+ 1H	2.0	2
8	539.1706	$C_{22}H_{35}O_{13}S$	539.1798	- 0.2 mDa	- $C_{44}H_{80}O_{20}S$	+ 1H	2.0	2

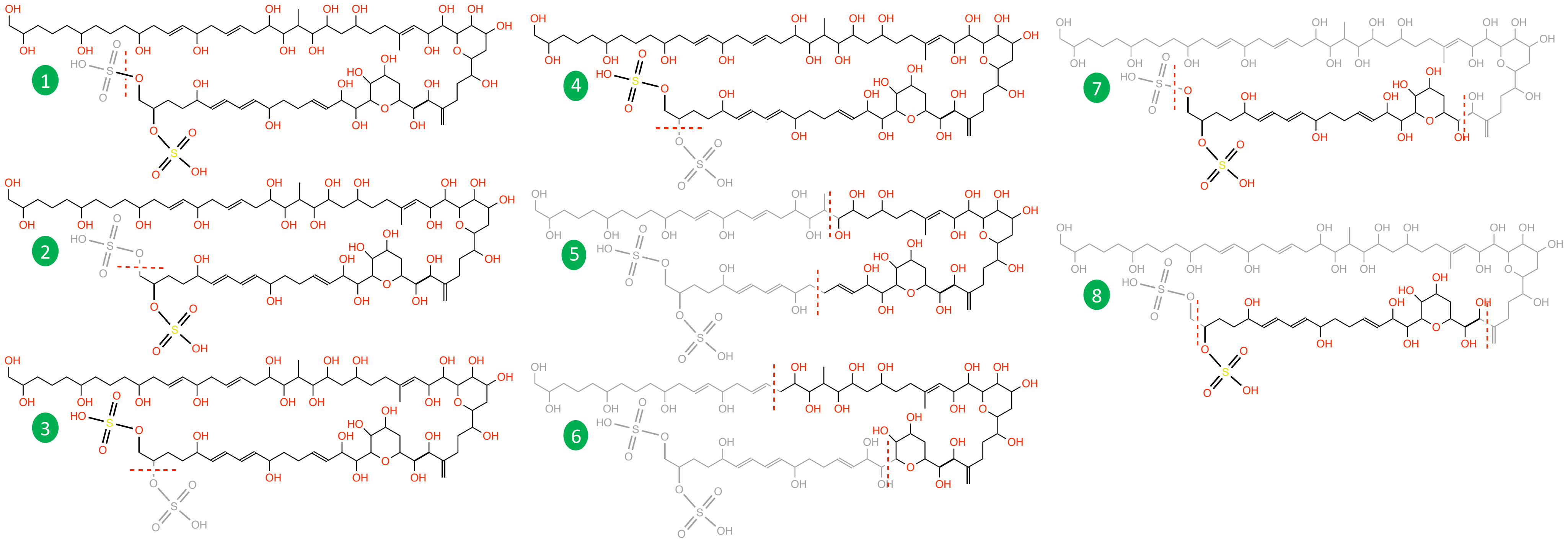

Figure S25. ^{1}H NMR spectrum (600 MHz, CD_3OD) for amphidinol 26.

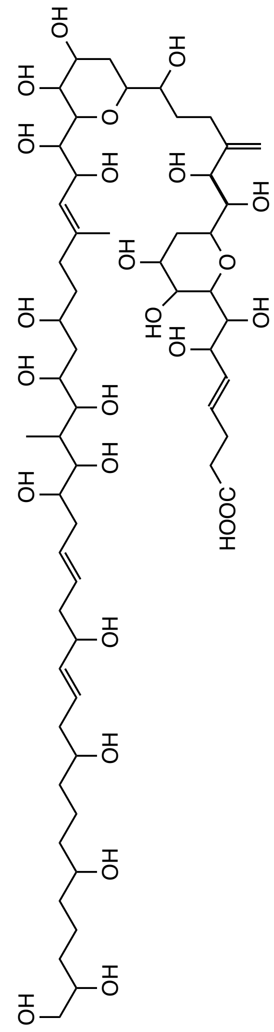

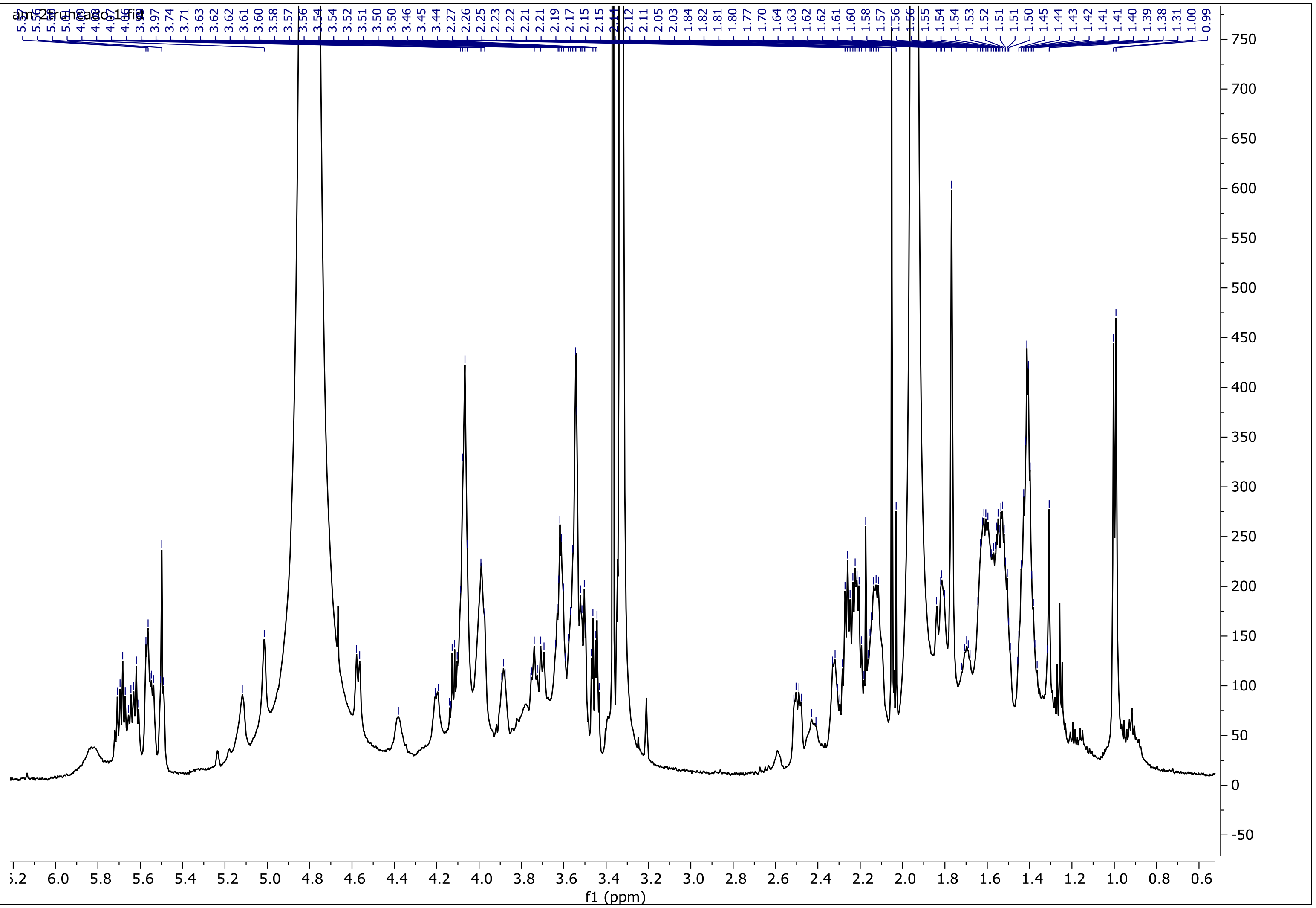

Figure S26. COSY spectrum (600 MHz, CD_3OD) for amphidinol 26.

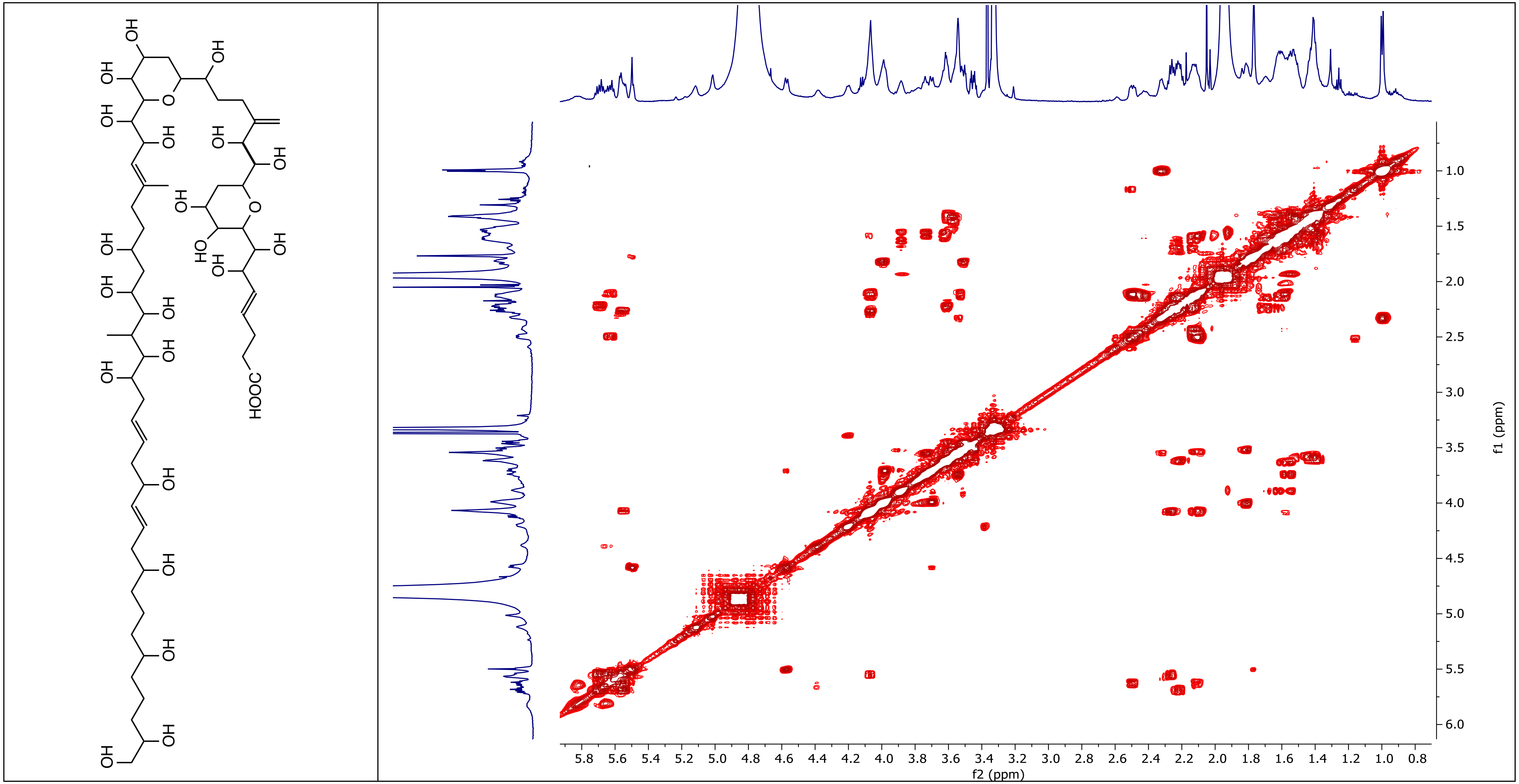

Figure S27. HSQC$_{ed}$ spectrum (600 MHz, CD_3OD) for amphidinol 26.

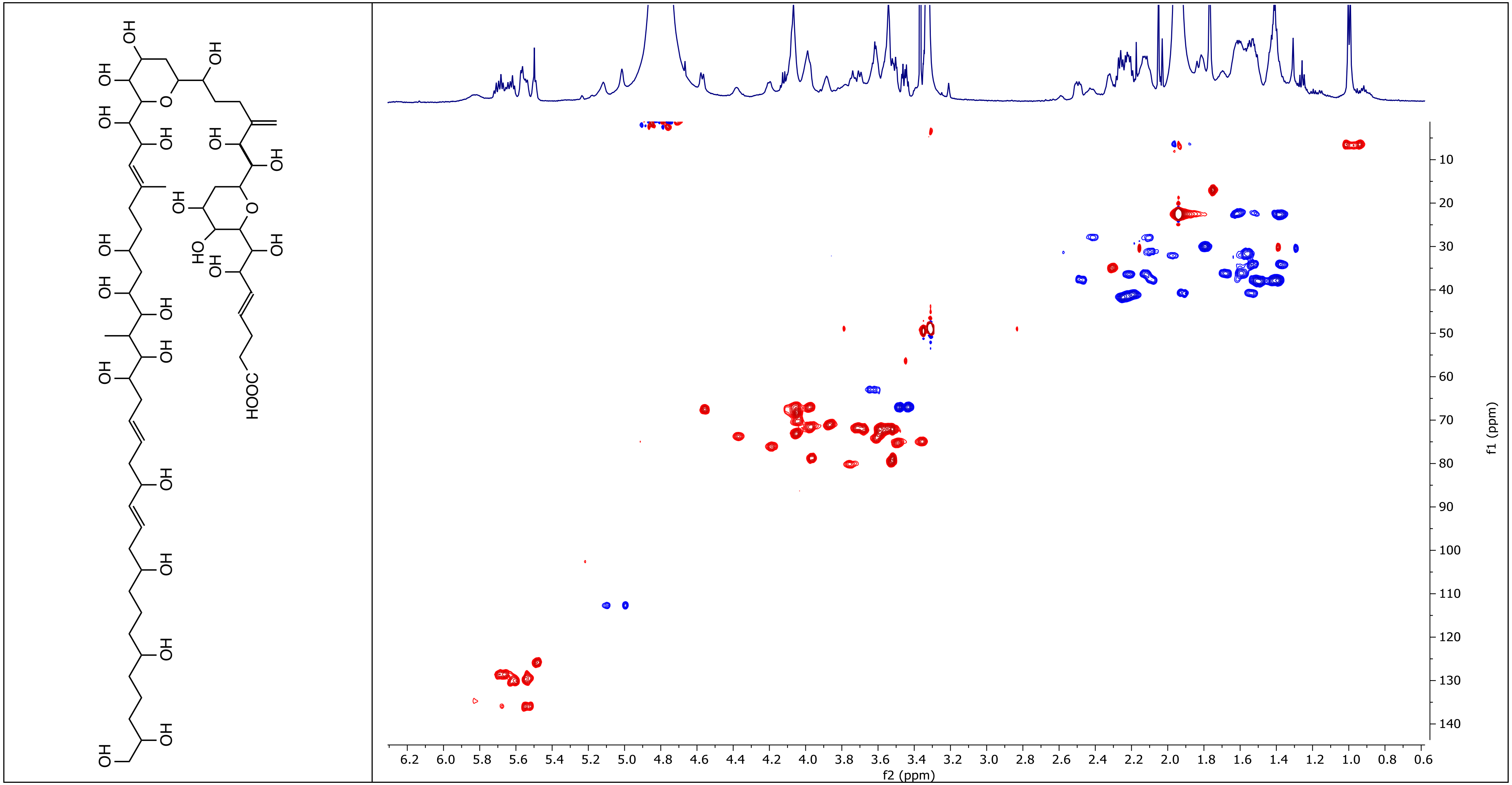

Figure S28. HSQC-TOCSY spectrum (600 MHz, CD_3OD) for amphidinol 26.

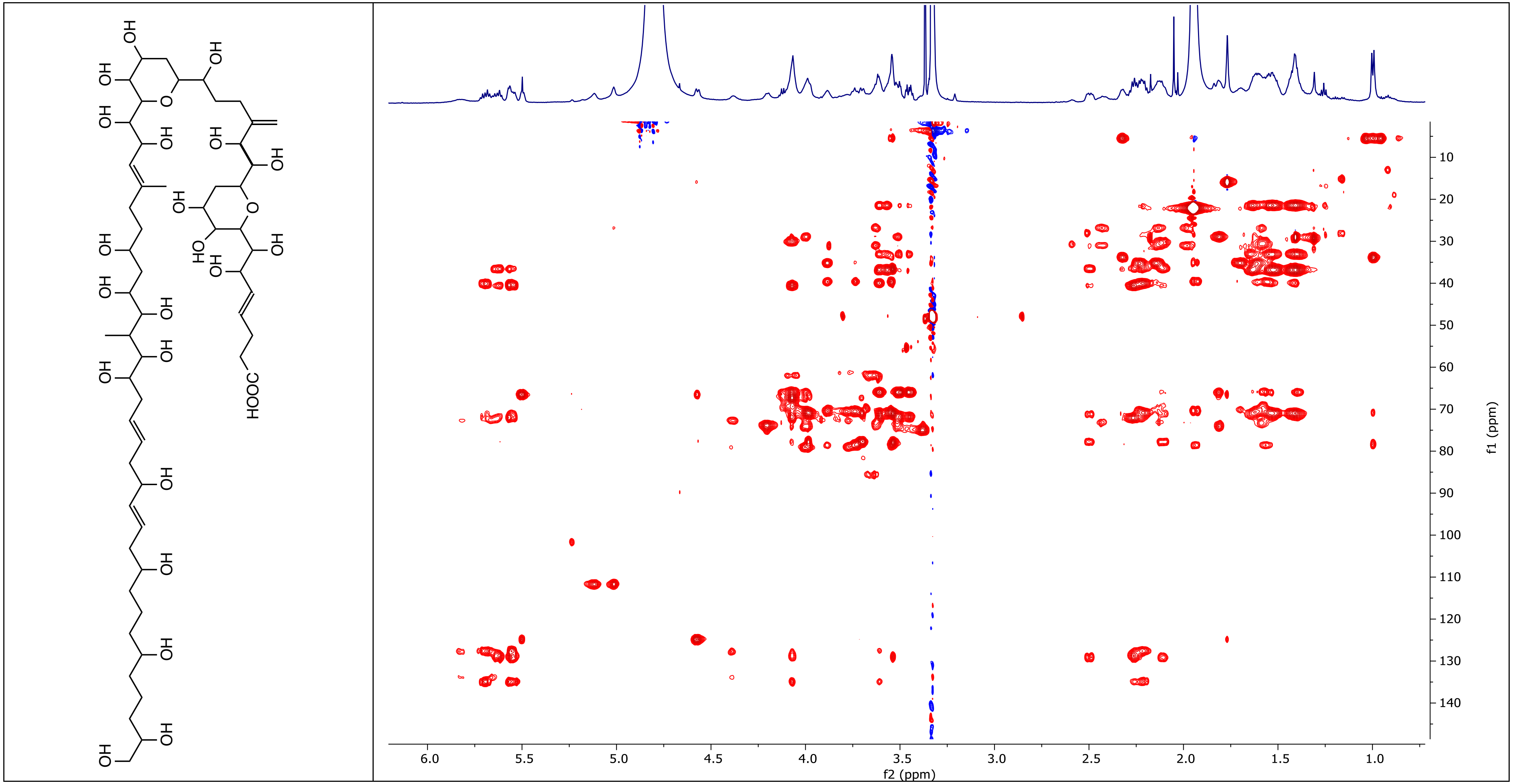

Figure S29. HMBC spectrum (600 MHz, CD_3OD) for amphidinol 26.

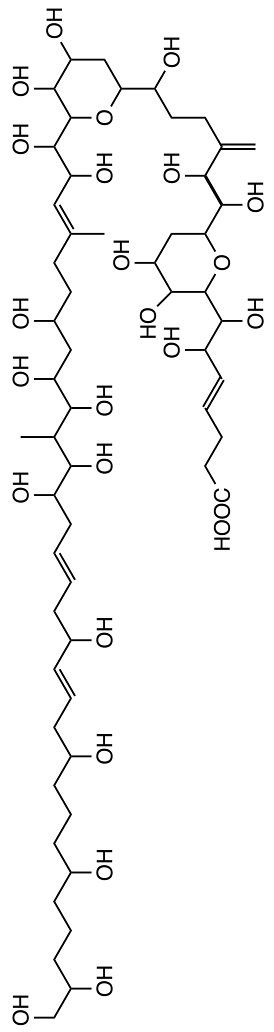

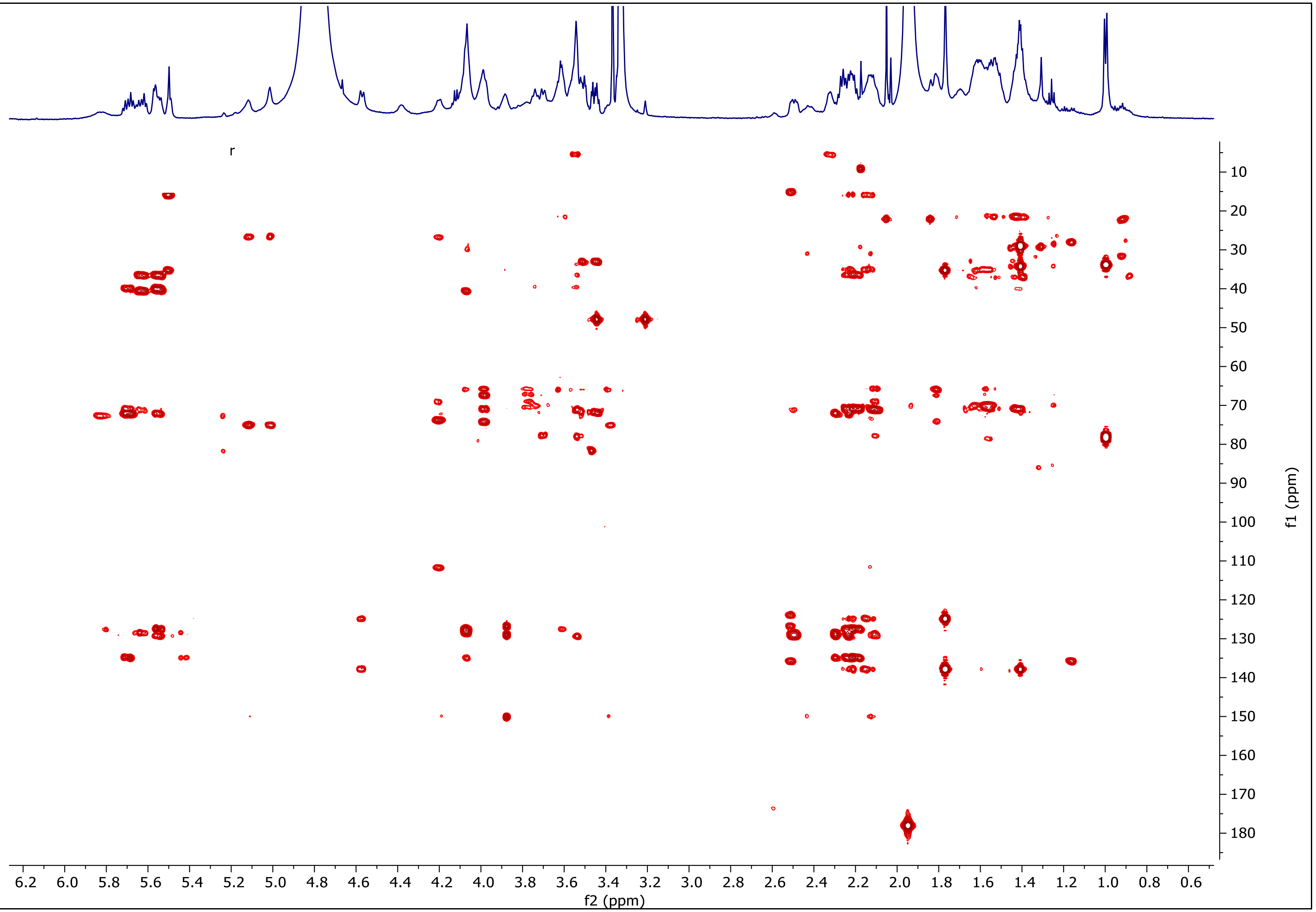

Figure S30. H2BC spectrum (600 MHz, CD_3OD) for amphidinol 26.

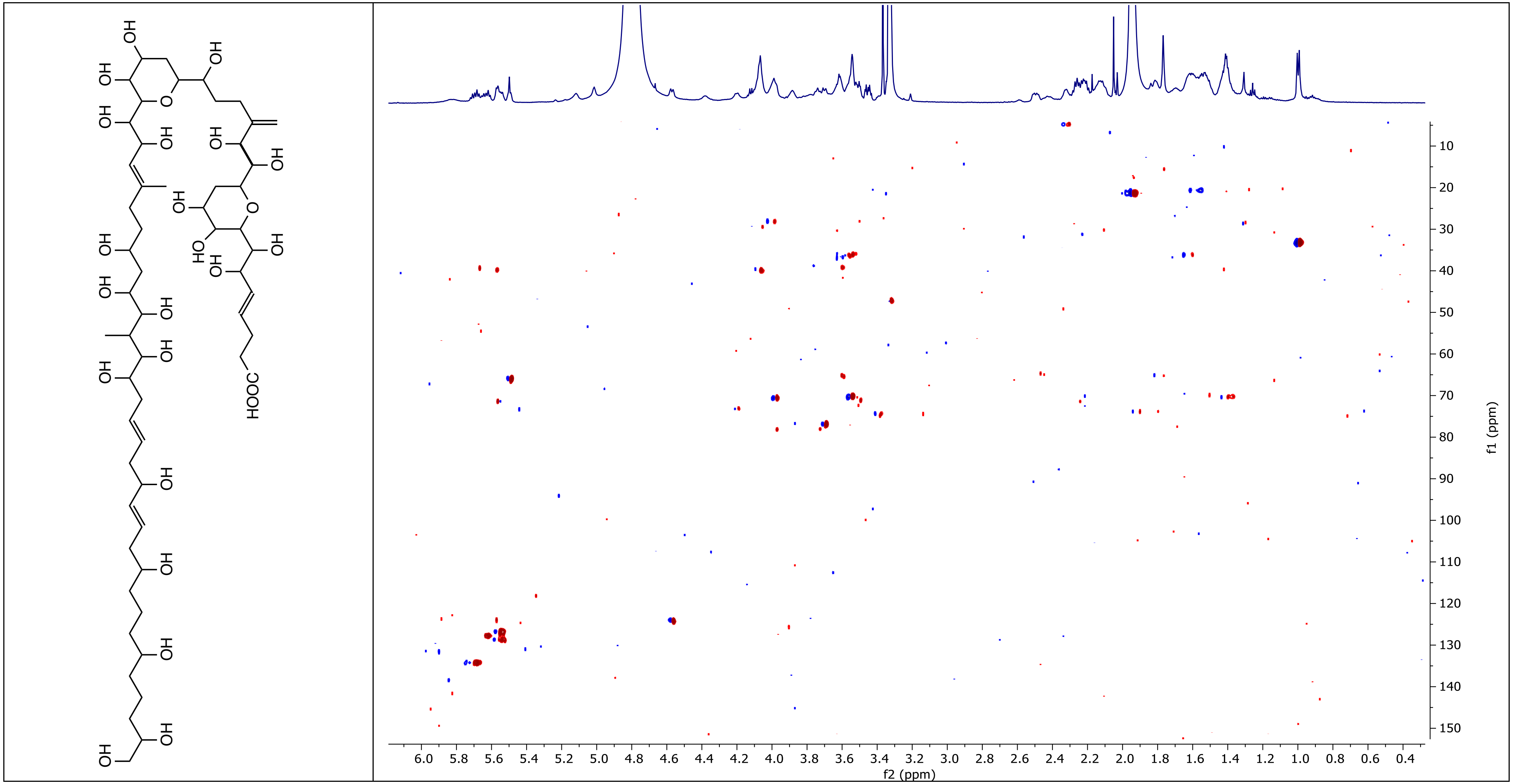

Figure S31. T-ROESY spectrum (600 MHz, CD_3OD) for amphidinol 26.

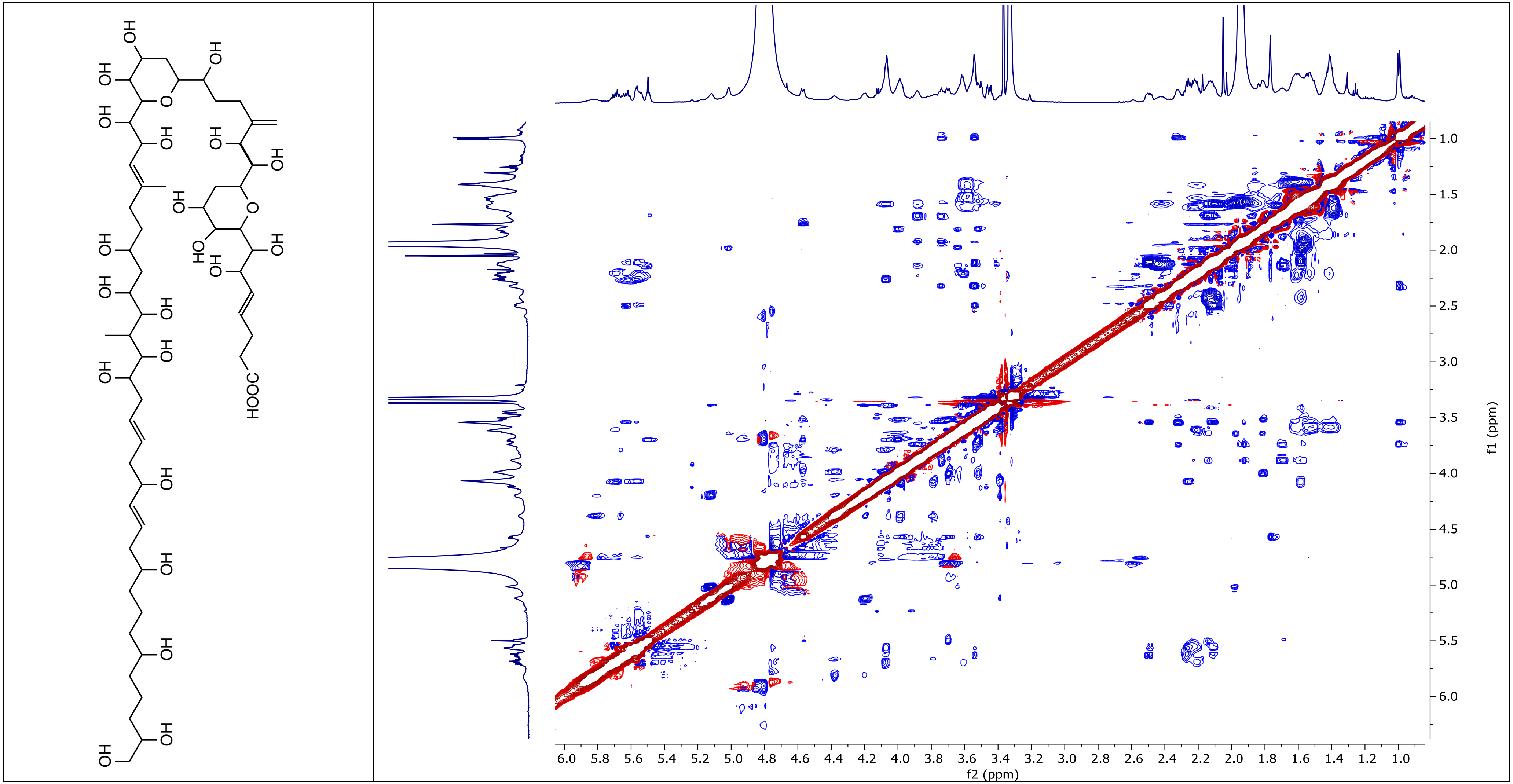

Figure S32. HRESIMS spectrum for amphidinol 26.

Elemental Composition Report **Page 1**

Multiple Mass Analysis: 3 mass(es) processed
Tolerance = 5.0 PPM / DBE: min = -1.5, max = 120.0
Element prediction: Off
Number of isotope peaks used for i-FIT = 3

Monoisotopic Mass, Even Electron Ions
325 formula(e) evaluated with 5 results within limits (all results (up to 1000) for each mass)
Elements Used:
C: 0-60 H: 0-110 O: 0-25 Na: 0-2
David
ESI (17-159) Adrian (ACO35CI8MAC) 51 (1.778)
2: TOF MS ES+
1.68e+002

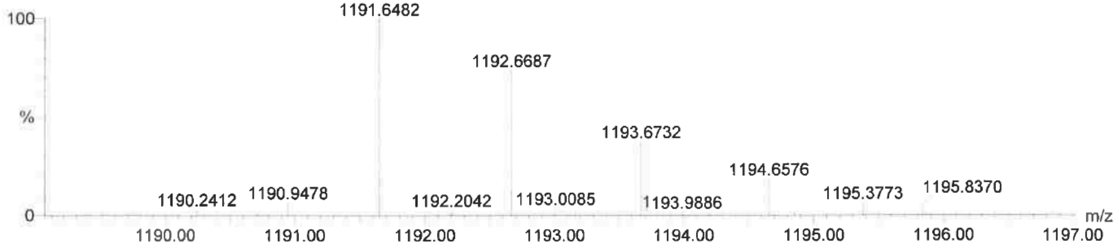

Minimum:	20.00				-1.5			
Maximum:	100.00		5.0	5.0	120.0			

Mass	RA	Calc. Mass	mDa	PPM	DBE	i-FIT	i-FIT (Norm)	Formula
1191.6482	100.00	1191.6526	-4.4	-3.7	10.5	26.5	0.5	C59 H99 O24
		1191.6502	-2.0	-1.7	7.5	27.2	1.3	C57 H100 O24 Na
		1191.6478	0.4	0.3	4.5	28.1	2.2	C55 H101 O24 Na2
1192.6687	73.82	---						
1193.6732	37.73	1193.6683	4.9	4.1	9.5	33.5	0.4	C59 H101 O24
		1193.6787	-5.5	-4.6	7.5	34.4	1.2	C59 H103 O21 Na2

Figure S33. Amphidinol 26 conversion from aldehyde to carboxylic acid at C-54 observed by ESI-HRMS.

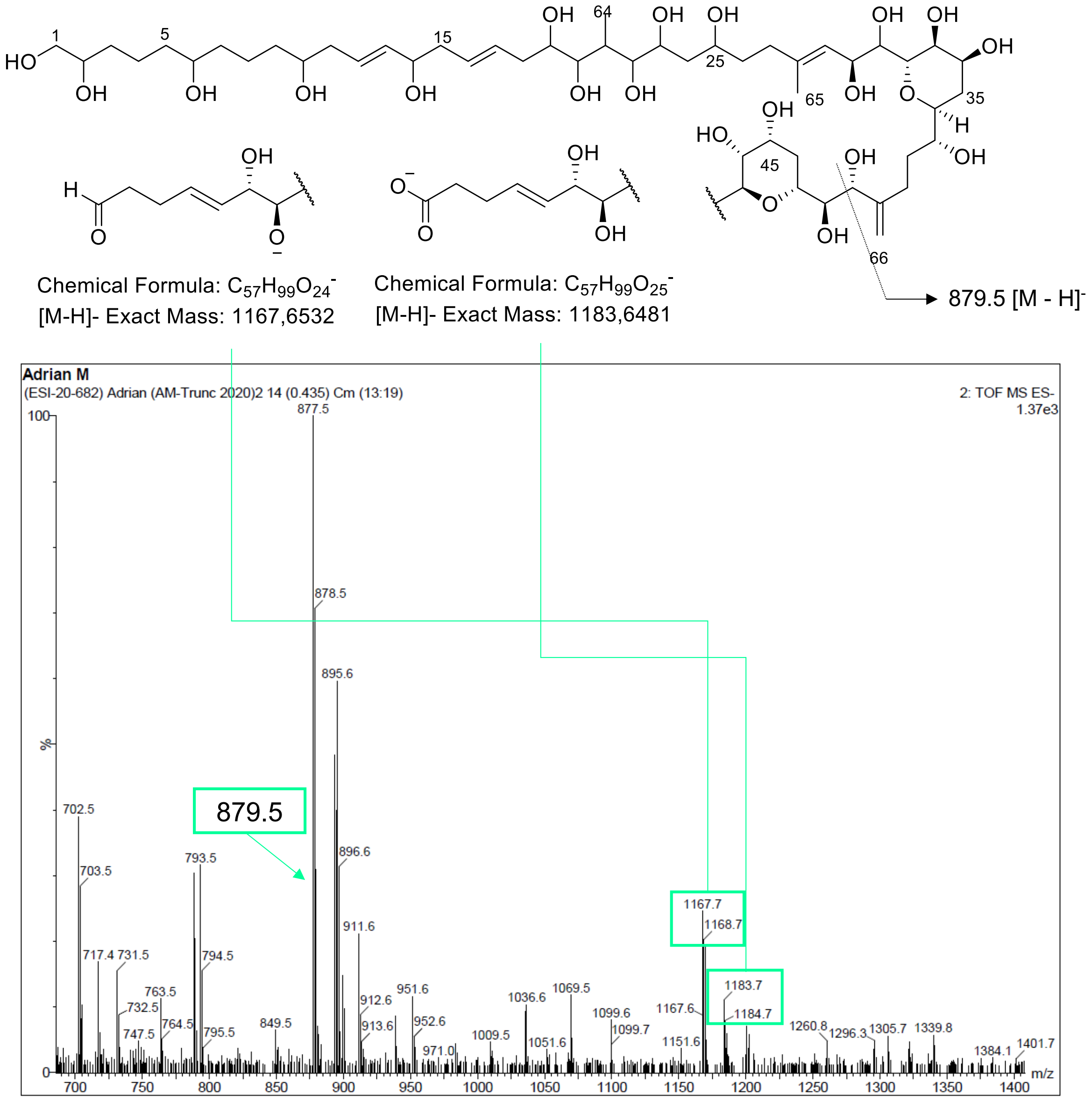

Figure S34. Amphidinol 26 single mass composition analysis for aldehyde and carboxylic states.

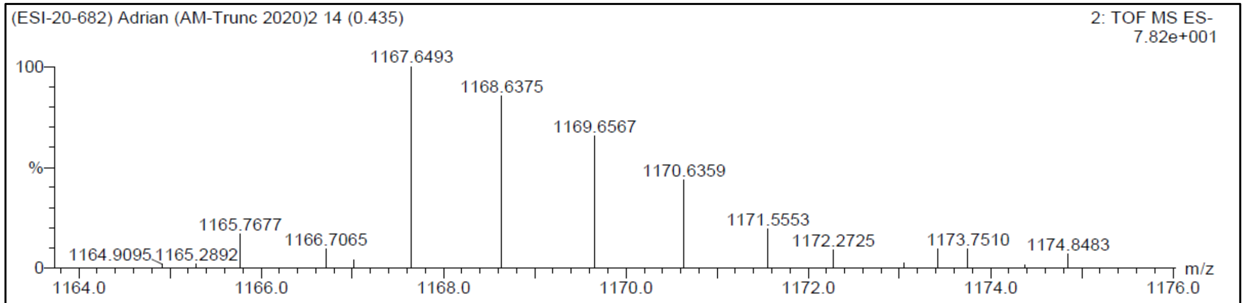

Minimum:	23.00				-3.0			
Maximum:	100.00		5.0	5.0	120.0			
Mass	RA	Calc. Mass	mDa	PPM	DBE	i-FIT	i-FIT (Norm)	Formula
1167.6493	100.00	1167.6502	-0.9	-0.8	5.5	14.7	0.6	C55 H100 O24 Na
		1167.6526	-3.3	-2.8	8.5	15.0	0.8	C57 H99 O24

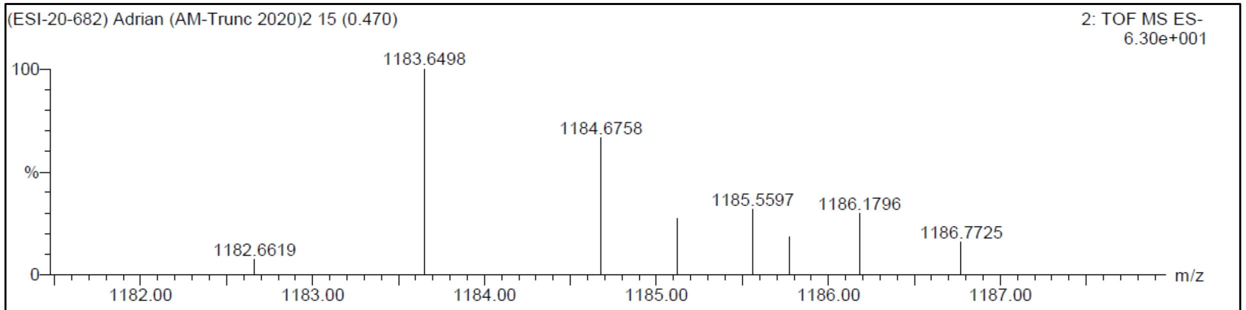

Minimum:	23.00				-3.0			
Maximum:	100.00		5.0	5.0	120.0			
Mass	RA	Calc. Mass	mDa	PPM	DBE	i-FIT	i-FIT (Norm)	Formula
1183.6498	100.00	1183.6475	2.3	1.9	8.5	26.9	0.7	C57 H99 O25
		1183.6451	4.7	4.0	5.5	26.9	0.7	C55 H100 O25 Na

Figure S35. Main MS/MS fragments observed for amphidinol 26 as aldehyde.

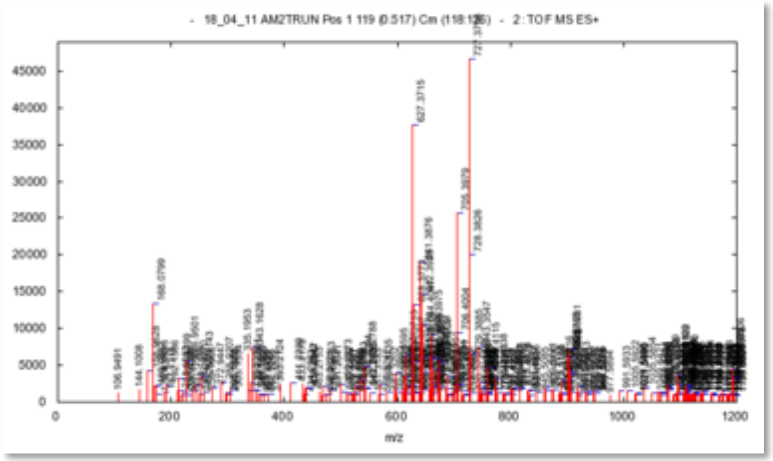

ID	*m/z*	Formula	Calc.	Deviation	Losses	Add.	Score	Bonds	ID	*m/z*	Formula	Calc.	Deviation	Losses	Add.	Score	Bonds
1	1153.6769	$C_{57}H_{101}O_{23}$	1153.6743	+ 3.4 mDa	- O	+ 2H	0.5	1	12	728.3831	$C_{33}H_{60}O_{17}$	728.3831	- 0.5 mDa	- $C_{24}H_{41}O_7$	+ 2H	2.0	2
2	1140.6281	$C_{55}H_{96}O_{24}$	1140.6292	- 1.1 mDa	- C2H_5	+ 0H	2.0	2	13	688.3976	$C_{35}H_{59}O_{13}$	687.3956	- 0.5 mDa	- $C_{22}H_{42}O_{11}$	- 1H	2.0	2
3	1111.6271	$C_{54}H_{95}O_{23}$	1111.6264	+ 0.7 mDa	- C_3H_6O	+ 0H	1.0	1	14	629.3107	$C_{31}H_{49}O_{13}$	629.3173	- 6.6 mDa	- $C_{26}H_{52}$O11	- 1H	1.5	2
4	1109.6536	$C_{55}H_{97}O_{22}$	1109.6471	+ 6.5 mDa	- $C_2H_4O_2$	+ 2H	1.0	1	15	581.3202	$C_{27}H_{49}O_{13}$	581.3173	+ 2.9 mDa	- $C_{30}H_{52}O_{11}$	+ 3H	2.0	2
5	1077.5829	$C_{53}H_{89}O_{22}$	1077.5845	- 1.6 mDa	- $C_4H_{12}O_2$	- 1H	2.0	0	16	581.3202	$C_{27}H_{49}O_{13}$	581.3173	+ 2.9 mDa	- $C_{30}H_{52}O_{11}$	+ 1H	2.0	2
6	1065.5861	$C_{52}H_{89}O_{22}$	1065.5845	+ 1.6 mDa	- $C_5H_{12}O_2$	+ 0H	1.0	1	17	481.2649	$C_{22}H_{41}O_{11}$	481.2649	- 0.8 mDa	- $C_{35}H_{60}O_{13}$	+ 1H	2.0	2
7	949.5388	$C_{47}H_{81}O_{19}$	949.5372	+ 1.2 mDa	- $C_{10}H_{20}O_5$	+ 3H	1.5	2	18	372.1876	$C_{18}H_{28}O_8$	372.1784	+ 9.2 mDa	- $C_{39}H_{73}O_{16}$	- 2H	2.0	2
8	923.4917	$C_{44}H_{75}O_{20}$	923.4852	+ 8.3 mDa	- $C_{13}H_{26}O_4$	+ 0H	1.0	1	19	357.1827	$C_{18}H_{29}O_7$	357.1913	- 8.6 mDa	- $C_{39}H_{72}O_{17}$	+ 1H	1.5	2
9	891.4882	$C_{44}H_{75}O_{18}$	891.4953	- 7.1mDa	- $C_{13}H_{26}O_6$	- 1H	2.0	2	20	305.1554	$C_{14}H_{25}O_7$	305.1554	- 4.6 mDa	- $C_{43}H_{76}O_{17}$	+ 3H	1.5	2
10	805.4586	$C_{40}H_{69}O_{16}$	805.4586	- 6.9 mDa	- $C_{17}H_{32}O_8$	- 1H	2.0	2	21	187.1318	$C_{10}H_{19}O_3$	187.1334	- 1.6 mDa	- $C_{47}H_{82}O_{21}$	- 1H	1.5	2
11	729.3885	$C_{35}H_{58}O_{15}$	729.3909	- 2.4 mDa	- $C_{24}H_{40}O_7$	+ 3H	2.0	2	22	169.0852	$C_9H_{13}O_3$	169.0865	- 1.3 mDa	- $C_{48}H_{88}O_{21}$	- 1H	2.0	2

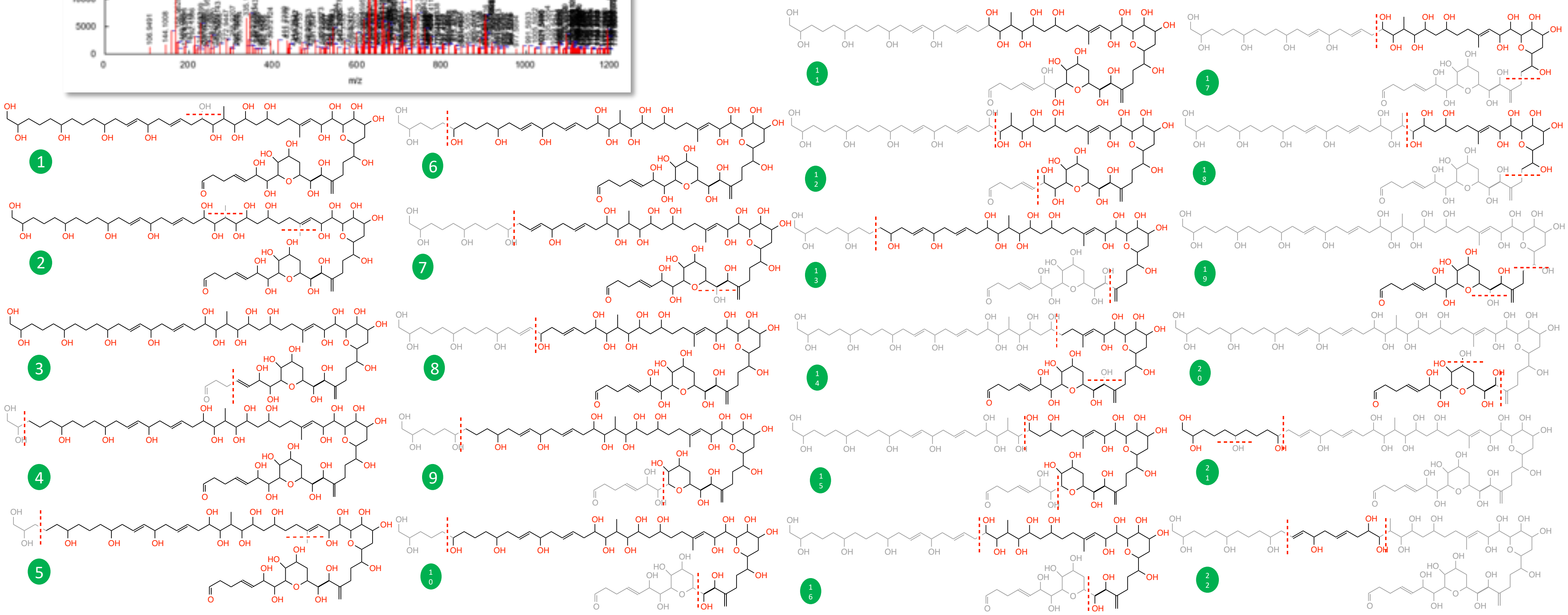

Figure S36. ^{1}H-NMR and $HSQC_{ed}$ spectra (600 MHz, CD_3OD) for luteophanol D.

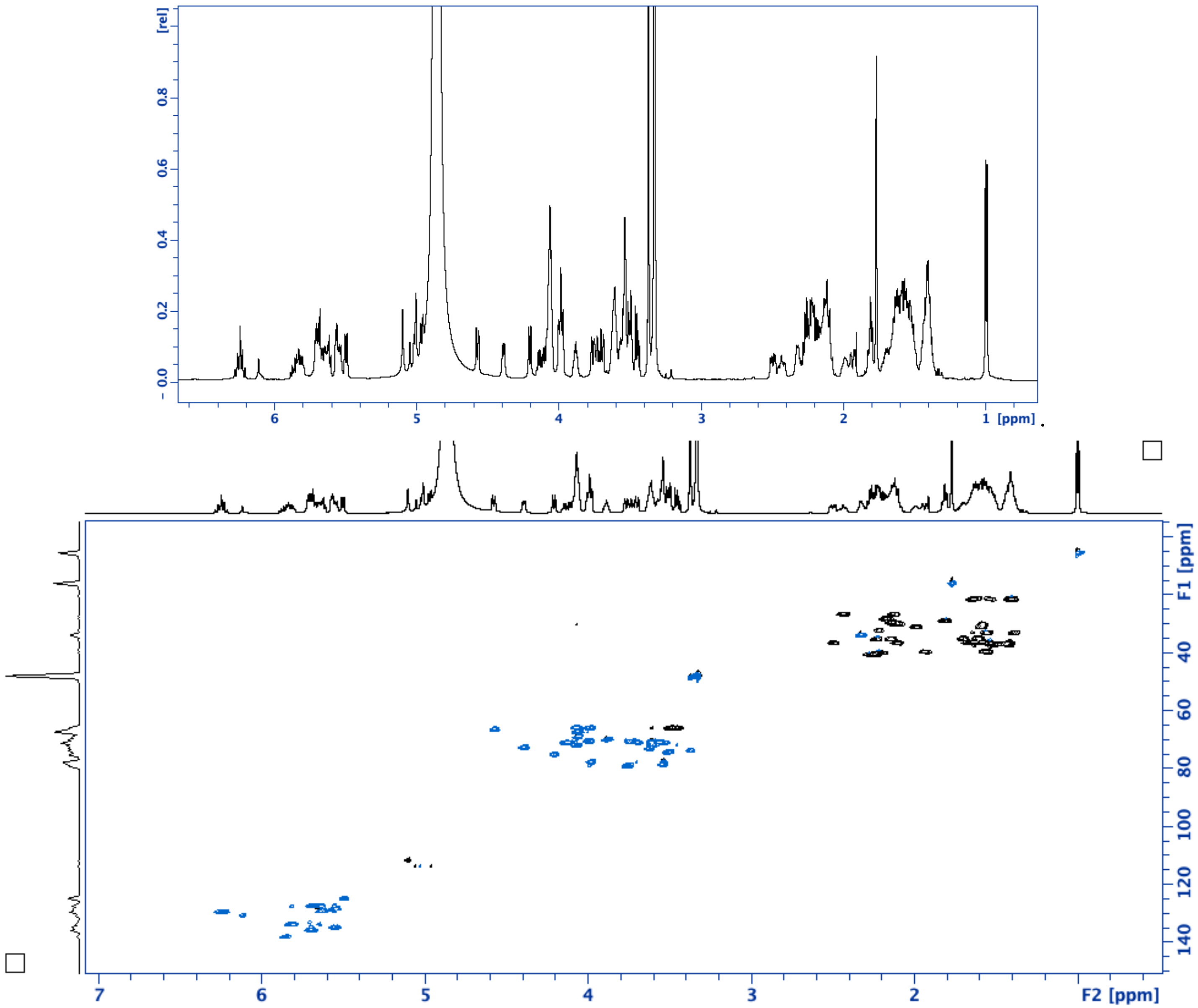

Figure S37. HRESIMS spectrum for luteophanol D.

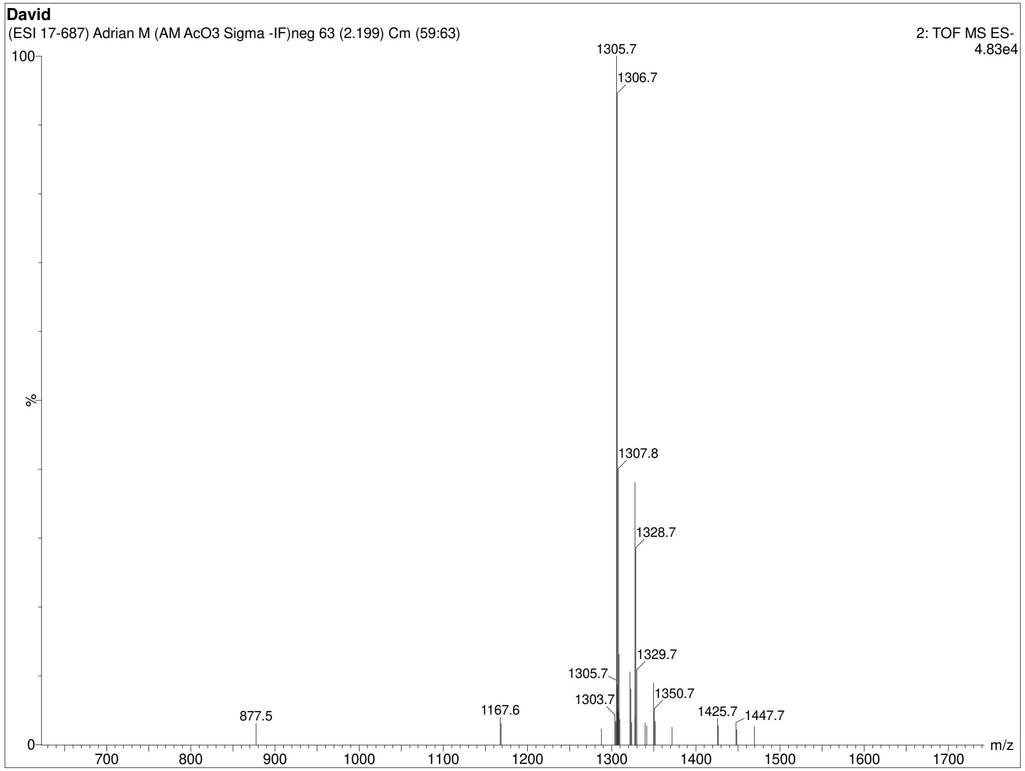

Elemental Composition Report **Page 1**

Multiple Mass Analysis: 3 mass(es) processed
Tolerance = 5.0 PPM / DBE: min = -1.5, max = 100.0
Element prediction: Off
Number of isotope peaks used for i-FIT = 3

Monoisotopic Mass, Even Electron Ions
906 formula(e) evaluated with 9 results within limits (up to 50 best isotopic matches for each mass)
Elements Used:
C: 0-80 H: 0-120 O: 0-30 Na: 0-1

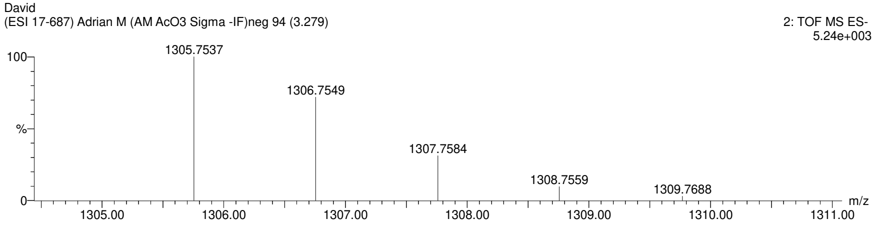

Minimum:	20.00				-1.5			
Maximum:	100.00		5.0	5.0	100.0			
Mass	RA	Calc. Mass	mDa	PPM	DBE	i-FIT	i-FIT (Norm)	Formula
1305.7537	100.00	1305.7547	-1.0	-0.8	7.5	10.8	0.0	C64 H114 O25 Na
		1305.7571	-3.4	-2.6	10.5	14.0	3.3	C66 H113 O25
		1305.7512	2.5	1.9	19.5	16.6	5.9	C73 H109 O20
		1305.7488	4.9	3.8	16.5	16.7	5.9	C71 H110 O20 Na
1306.7549	71.82	---						
1307.7584	31.12	1307.7575	0.9	0.7	5.5	20.6	1.0	C62 H115 O28
		1307.7551	3.3	2.5	2.5	20.7	1.0	C60 H116 O28 Na
		1307.7586	-0.2	-0.2	24.5	21.5	1.8	C78 H108 O15 Na
		1307.7610	-2.6	-2.0	27.5	22.2	2.5	C80 H107 O15
		1307.7645	-6.1	-4.7	15.5	23.2	3.5	C71 H112 O20 Na

Figure S38. ^{1}H-NMR and HSQC$_{ed}$ spectra (600 MHz, CD_3OD) for amphidinol 20B.

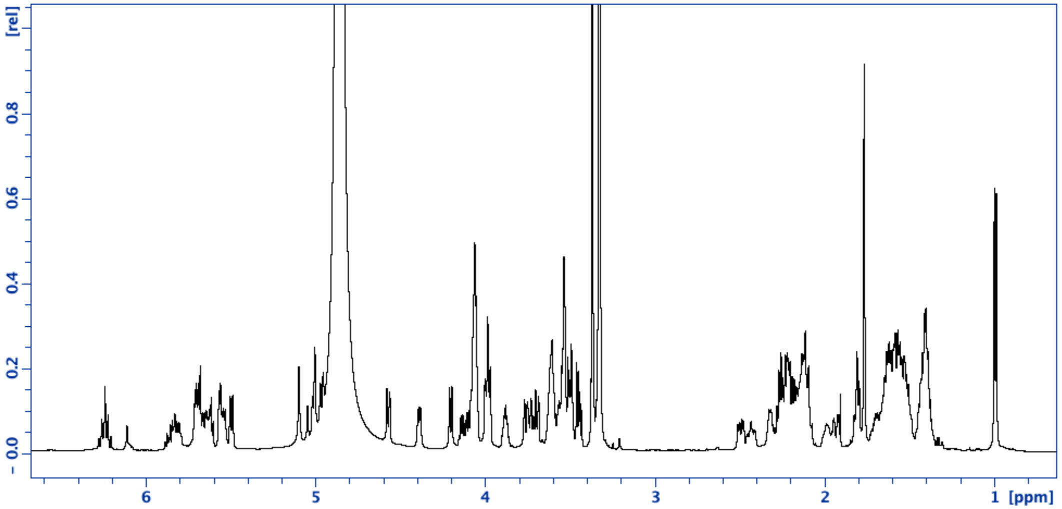

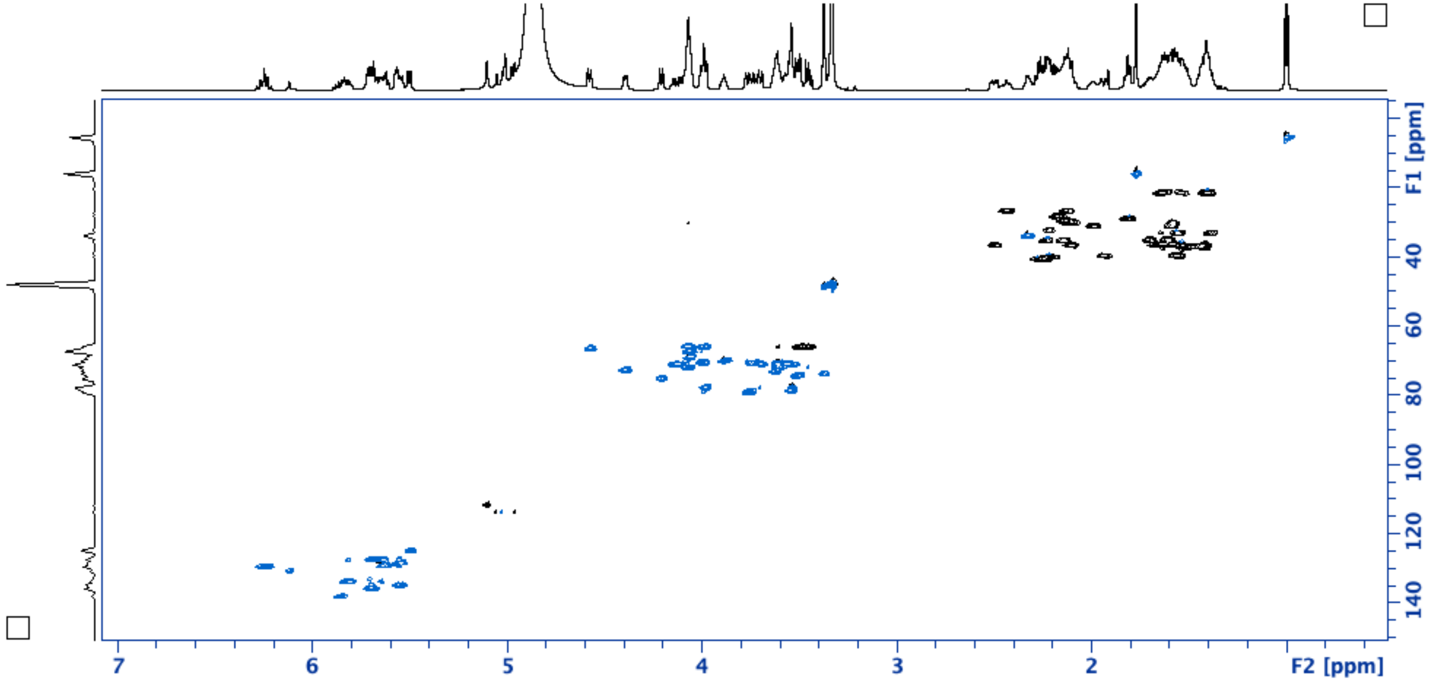

Figure S39. HRESIMS spectrum for amphidinol 20B.

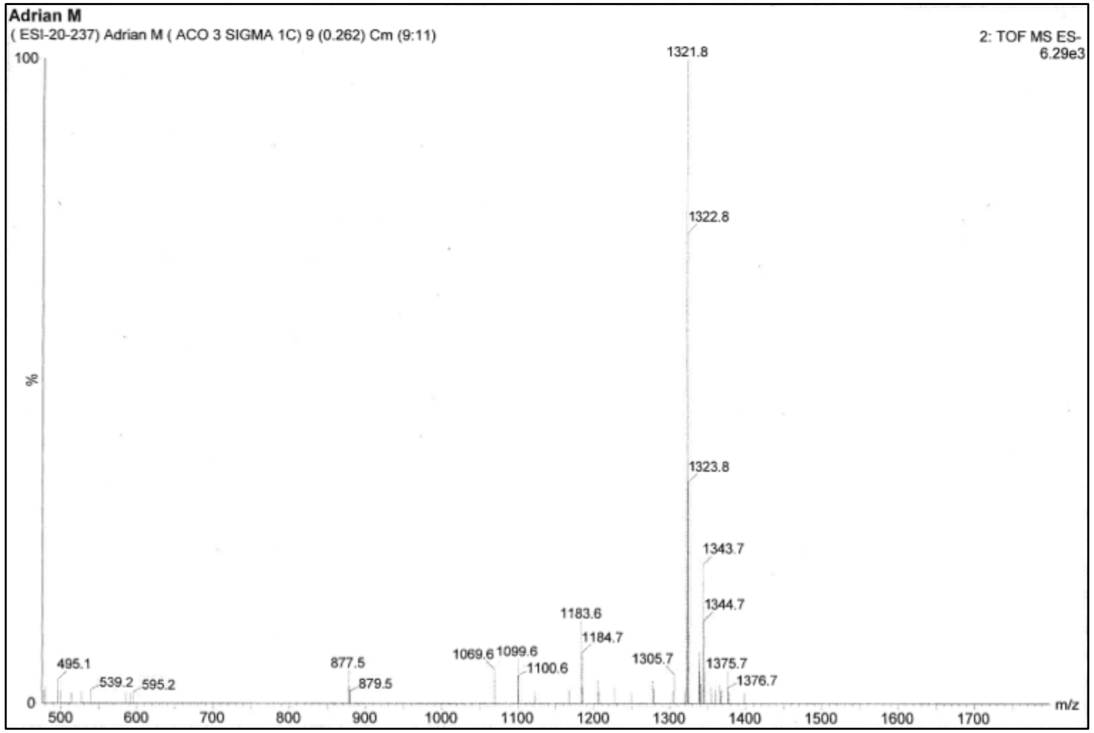

Elemental Composition Report

Page 1

Multiple Mass Analysis: 3 mass(es) processed
Tolerance = 5.0 PPM / DBE: min = -1.5, max = 100.0
Element prediction: Off
Number of isotope peaks used for i-FIT = 3

Monoisotopic Mass, Even Electron Ions
847 formula(e) evaluated with 8 results within limits (up to 50 best isotopic matches for each mass)
Elements Used:
C: 0-80 H: 0-120 O: 0-30 Na: 0-1
Adrian M
(ESI-20-237) Adrian M (ACO 3 SIGMA 1C) 34 (1.462)

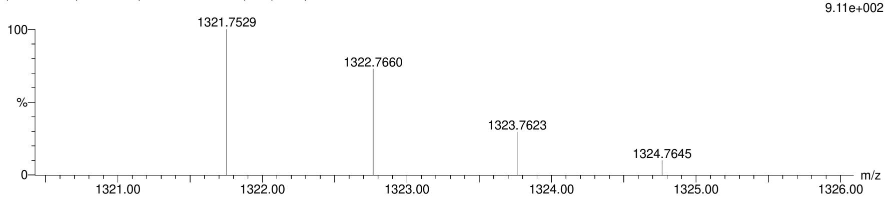

Mass	RA	Calc. Mass	mDa	PPM	DBE	i-FIT	i-FIT (Norm)	Formula
Minimum:	20.00				-1.5			
Maximum:	100.00		5.0	5.0	100.0			
1321.7529	100.00	1321.7496	3.3	2.5	7.5	13.1	0.7	C64 H114 O26 Na
		1321.7520	0.9	0.7	10.5	13.2	0.8	C66 H113 O26
		1321.7590	-6.1	-4.6	20.5	15.8	3.4	C75 H110 O18 Na
1322.7660	72.80	---						
1323.7623	29.39	1323.7653	-3.0	-2.3	6.5	22.9	1.5	C64 H116 O26 Na
		1323.7618	0.5	0.4	18.5	23.0	1.5	C73 H111 O21
		1323.7594	2.9	2.2	15.5	23.0	1.6	C71 H112 O21 Na
		1323.7677	-5.4	-4.1	9.5	23.1	1.6	C66 H115 O26
		1323.7559	6.4	4.8	27.5	23.3	1.9	C80 H107 O16
